# An integrative and comprehensive analysis of blood transcriptomes combined with machine learning models reveals key signatures for tuberculosis diagnosis and risk stratification

**DOI:** 10.3389/fmicb.2025.1546770

**Published:** 2025-05-26

**Authors:** Maryam Omrani, Arash Ghodousi, Daniela Maria Cirillo

**Affiliations:** ^1^Emerging Bacterial Pathogens Unit, IRCCS San Raffaele Scientific Institute, Milan, Italy; ^2^Università Vita Salute San Raffaele, Milan, Italy

**Keywords:** *Mycobacterium tuberculosis*, biomarkers, blood, RNA-seq, machine-learning

## Abstract

Tuberculosis (TB) remains a major global health challenge, contributing substantially to morbidity and mortality worldwide. The progression from *Mycobacterium tuberculosis* (Mtb) infection to active disease involves a complex interplay between host immune responses and Mtb’s ability to evade them. However, current diagnostic tools, such as interferon-gamma release assays (IGRAs) and tuberculin skin tests (TSTs), have limited ability to distinguish between different stages of TB or to predict the progression from infection to active disease. In this study, we performed an integrative analysis of 324 previously acquired blood transcriptome samples from TB patients, TB contacts, and controls across diverse geographical regions. Differential gene expression analysis revealed distinct transcriptomic signatures in TB patients, highlighting dysregulated pathways related to immune responses, antimicrobial peptides, and extracellular matrix organization. Using machine learning, we identified a 99-transcript signature that accurately distinguished TB patients from controls, demonstrated strong predictive performance across different cohorts, and identified potential progressors or subclinical cases. Validation in an independent dataset comprising 90 TB patients and 20 healthy controls confirmed the robustness of the 10-gene signature (BATF2, FAM20A, FBLN2, AK5, VAMP5, MMP8, KLHDC8B, LINC00402, DEFA3, and GBP6), achieving high area under the curve (AUC) values in both receiver operating characteristic (ROC) and precision–recall analyses. This 10-gene signature offers promising candidates for further validation and the development of diagnostic and prognostic tools, supporting global efforts to improve TB detection and risk stratification.

## 1 Introduction

Tuberculosis (TB), caused by *Mycobacterium tuberculosis* (Mtb), remains a global health crisis, causing over one million deaths annually despite advancements in diagnostic tools and treatment strategies (World Health Organization, 2023a). Historically, TB has been dichotomized into latent TB infection (TBI), a clinically asymptomatic stage without microbiological evidence of active disease, and active TB (ATB), characterized by overt clinical symptoms and microbial detection (Cobelens et al., 2017; Kiazyk and Ball, 2017; Behr et al., 2024). However, this dichotomic classification of TB pathogenesis is currently being reconsidered as emerging evidence highlighting the importance of dynamic interactions between host and Mtb. This spectrum includes people who have cleared TB, individuals still harboring live bacteria or those with subclinical or incipient TB. In this context, TBI shows a persistent immune response to Mtb antigens without clinical evidence of active disease while maintaining its viability with the potential to replicate and cause symptomatic disease (Behr et al., 2024; Lee, 2016; Singhania et al., 2018a; Drain et al., 2018; Larsson et al., 2024).

Approximately 5–10% of individuals infected with Mtb progress to ATB within months to 2 years of initial infection (Behr et al., 2024; Lee, 2016; Singhania et al., 2018a). Therefore, TBI is increasingly recognized as a critical component of the global programmatic TB control efforts. The World Health Organization’s (WHO) aim to achieve the “End TB Strategy” targets recommends the early diagnosis and treatment of people with TBI who are at high risk of progression as a critical step to eliminate TB (Agbota et al., 2023). However, given the cost of intervention, potential toxicity, and adverse effects of treatment, identifying individuals at high risk of TB progression using non-invasive approaches would increase the benefits of preventive therapy (PT).

Current diagnostic tools for TBI, including interferon-gamma release assay (IGRA) and tuberculin skin test (TST), cannot differentiate between ATB and TB exposure with persistent infection (TBI), nor can they predict progression from TBI to ATB (Behr et al., 2024; Lee, 2016; Singhania et al., 2018a; Drain et al., 2018). In addition, multiple studies have shown that patients who have undergone PT retain immunoreactivity to the inactivated Mtb protein fraction used in the TST and to specific Mtb antigens used in IGRA (Behr et al., 2024). On the other hand, the early secreted antigenic target of 6 kDa (ESAT-6) has been shown to inhibit the release of IFN-*γ* by human T cells (Wang et al., 2009), potentially reducing the sensitivity of these diagnostic tools. Therefore, the development of simple and scalable methods to identify individuals at high risk of TB progression is essential for optimizing the impact and cost-effectiveness of PT.

Gene expression profiling of blood transcriptomes offers a powerful approach to investigate the immune system alterations in TB (Maertzdorf et al., 2011; Estévez et al., 2020; Kwan et al., 2020; Van Doorn et al., 2022; Thompson et al., 2017). However, the mechanisms that determine the potential outcome of TB infection are not thoroughly understood. Furthermore, gene expression data are characterized by high dimensionality, and only a limited number of studies have utilized gene expression profiles alongside data mining techniques to reduce the dimensionality, identify discriminative genes, learn diagnostic patterns, and predict high-risk TBI cases likely to progress to active disease. In addition, these studies often rely on single-source datasets for both model training and prediction, limiting their generalizability (Estévez et al., 2020). To develop robust and globally applicable predictive models, integrated transcriptomic data from diverse populations and settings—accounting for biological and technical variability—must maintain high predictive performance and generalizability across contexts.

In this study, we leveraged a comprehensive dataset comprising 324 blood transcriptome samples collected from five countries with distinct TB epidemiological profiles: South Africa, Mozambique, Spain, Singapore, and Indonesia. South Africa and Mozambique are among the countries with the highest TB incidence globally, largely driven by high rates of HIV co-infection and limited healthcare resources (World Health Organization, 2023b). Indonesia, currently ranked third in global TB burden, faces significant challenges in detection and treatment (United States Agency for International Development (USAID), 2021). Singapore, classified as a medium TB burden country, reported that migrants accounted for 49% of all notified active TB cases in 2017 (Chee et al., 2018; Lim et al., 2021). Spain, representing a low-burden country, has recorded TB cases primarily among immigrant populations from higher burden regions (Abascal et al., 2019). This dataset encompassed a broad array of subgroups, including TB patients, contacts, and controls with and without immunoreactivity, offering a robust platform for cross-population transcriptome analysis.

By combining next-generation sequencing with advanced data analysis techniques, our approach aims to elucidate the dynamic transitions from TB infection to active disease. To achieve this, we first prioritized TB- and control-distinguishing signatures using differential gene expression analysis. This was followed by binary classification and feature importance analysis to retain the most relevant transcriptomic markers. Using this refined feature set, we identified IGRA/TST + contacts whose blood transcriptomic profiles closely resembled those of TB patients. We further leveraged this reduced feature set to study distinct TB subgroups in greater detail. Finally, the top 10 ranked features were validated on an independent dataset to confirm their robustness.

## 2 Methods

### 2.1 Data

Whole-blood mRNA sequencing data were obtained from four publicly available studies—PRJEB31975, PRJNA595691, PRJNA798683, and PRJNA352062—through the NCBI Gene Expression Omnibus (GEO).^1^

- PRJEB31975 includes 65 active tuberculosis (ATB) cases, 43 IGRA/TST + contacts, and 50 controls (TB contacts with negative immunoreactivity).
- PRJNA595691 includes 14 ATB cases, 26 contacts, and 5 controls (volunteers with no recent TB exposure, with or without positive immunoreactivity).
- PRJNA798683 includes 11 ATB baseline samples suitable for inclusion in this study.

The integrated dataset comprised 90 ATB cases, 43 IGRA/TST + contacts, 26 contacts, and 55 controls (Table 1).

**Table 1:** Demographic composition of the cohorts.

Data sources	Active TB	IGRA/TST + contacts	Contacts	Controls	Country of samples
PRJEB31975					
Total (female: male)	65 (17:48)	43 (20:23)	0	50 (27:23)	Mozambique, Spain
Age mean (range)	35.3 (13–72)	42.0 (8–71)		38.5 (9–80)	
PRJNA798683					
PRJNA798683 (information of age and sex is not available)	11	0	0	0	Indonesia, South Africa
PRJNA595691					
Total (female: male)	14 (4:10)	0	26 (14:12)	5 (0:5)	Singapore
Age mean (range)	50.1 (27–69)		41.6 (22–66)	29.6 (24–38)	
PRJNA352062					
PRJNA352062 (information of age and sex is not available)	90			20	South Africa

In this study, TB contacts from the PRJEB31975 dataset who tested positive for immunoreactivity via IGRA and/or TST are referred to as “IGRA/TST + contacts.” However, “contacts” refers to TB-exposed individuals from the PRJNA595691 dataset, of which approximately 50% were IGRA-positive according to the original study (Estévez et al., 2020; Kwan et al., 2020). Due to the unavailability of individual-level IGRA and TST results, the two contact groups were analyzed separately.

PRJNA352062 was used as an independent validation set, focusing on baseline samples from ATB subjects and controls. This dataset included 20 healthy controls and 90 ATB cases.

In all studies, ATB individuals were diagnosed with TB shortly before blood sampling. Table 2 summarizes the inclusion and exclusion criteria applied in the original studies.

**Table 2:** Summary of inclusion and exclusion criteria applied in the original studies for each experimental group.

Group	Inclusion criteria	Exclusion criteria
Active tuberculosis (ATB) (PRJEB31975, PRJNA595691, PRJNA798683, and PRJNA352062)	Clinically diagnosed and microbiologically confirmed TB	Age < 18, pregnant women, diabetes, requiring chemotherapy, co-infected with HIV, having received anti-TB treatment recently, and previous TB diagnosis
IGRA/TST + contacts (PRJEB31975)	Healthy people exposed to a pulmonary microbiologically confirmed TB index case	Age < 18, pregnant women diabetes, requiring chemotherapy, co-infected with HIV, previous TB diagnosis, previous positive TST/IGRA documented, previous old healed lesion on chest radiography, recent (<3 months) vaccination with live-attenuated strains, any other active infection during the previous month, IGRA result indeterminate and having received anti-TB treatment before
Contacts (PRJNA595691)	Close household contact of patients with smear positive pulmonary TB	Age < 18, pregnant women, diabetes, requiring chemotherapy, co-infected with HIV, having received anti-TB treatment recently, having no evidence of active clinical TB, IGRA result indeterminate, and past history of TB
Controls (PRJEB31975,PRJNA595691,PRJNA352062)		Age < 18, pregnant women, diabetes, requiring chemotherapy, co-infected with HIV, sign of TB and other active infections, having received anti-TB treatment, and past history of TB

The datasets included in this study were selected based on the following criteria:

- Sample type: Only datasets driven from human whole-blood samples were included. Studies involving non-human sources or other sample types (e.g., peripheral blood mononuclear cells [PBMCs]) were excluded.
- Medical condition: Datasets containing samples from individuals co-infected with HIV or other additional diagnoses were excluded.
- Inclusion of both sexes: Each experimental group was required to include both male and female participants.
- Geographic diversity: Datasets were selected from multiple countries to capture population-level variability and enhance generalizability.
- Data type: Only RNA sequencing (RNA-seq) datasets were considered. Studies utilizing microarray platforms were excluded.

### 2.2 Gene expression quantification and downstream analysis

All raw reads are aligned with the human reference genome (hg38/GRCh38) using STAR (v2.5.3a).

Post-alignment QC including quantification of mapped reads on unique regions and coding sequences was conducted using the MultiQC (v1.9.0) tool. Mapping reads were counted from BAM files with featureCounts (v. 1.6.4), and Ensemble basic annotation was used to quantify expression levels. Differentially expressed (DE) genes between groups were identified using Limma-Voom functions from the edgeR package (v.38.4) following normalization by DESeq2. Batch correction was performed for different conditions (ATB, IGRA/TST + contacts, contacts, and controls) and the source of the data (Spain, Mozambique, South Africa, Indonesia, and Singapore): design = ~ condition + country. Genes with an adjusted *p*-value (p.adj) < 0.05 and an absolute log2 (fold-change) > 1 were considered significant in terms of differential expression. While a stricter threshold (e.g., log2 (fold-change) > 1.5) is sometimes used, we selected this cutoff to ensure the inclusion of biologically meaningful genes, which is consistent with the aims of our integrative analysis. This approach has also been applied in previous studies using comparable cohorts and methodologies (Estévez et al., 2020; Kwan et al., 2020). The list of DE genes from each comparison was analyzed for pathway enrichment using EnrichR (Reactome), available at: https://maayanlab.cloud/Enrichr/. The results were then visualized using the ggplot2 package in R. To assess global transcriptional differences across the four experimental groups, we performed an ANOVA-style analysis using the topTableF() function from the limma package, following the voom transformation for RNA-seq count data. The design matrix included the experimental group as the main variable of interest and country (design = ~ condition + country). Normalization and variance modeling were conducted using voom(), after which linear models were fit with lmFit() and moderated with eBayes(). The topTableF() function was then used to compute F-statistics and false discovery rates (FDRs) for each gene. Genes with FDR < 0.01 were considered significantly differentially expressed across the groups. These genes were subsequently subjected to pathway enrichment analysis.

### 2.3 Preprocessing of dataset for classification task

To prepare the transcriptome dataset for classification, transcripts were retained if expressed in at least 26 subjects (the number of subjects in the smallest group) according to the R package edgeR (v3.26.8). For outlier detection, count per million (CPM) was calculated for the retained transcripts. Next, the similarity among all subjects was calculated using the Pearson correlation coefficient, and Z scores were calculated for the correlation matrix (Omrani et al., 2024). Subjects with a Z score less than −2 were identified as outliers (ERR3258186 and ERR3258088; an ATB subject and an IGRA/TST + contact from the Mozambique cohort, respectively) and excluded from the analysis.

### 2.4 Binary classification workflow

**Figure 1:**
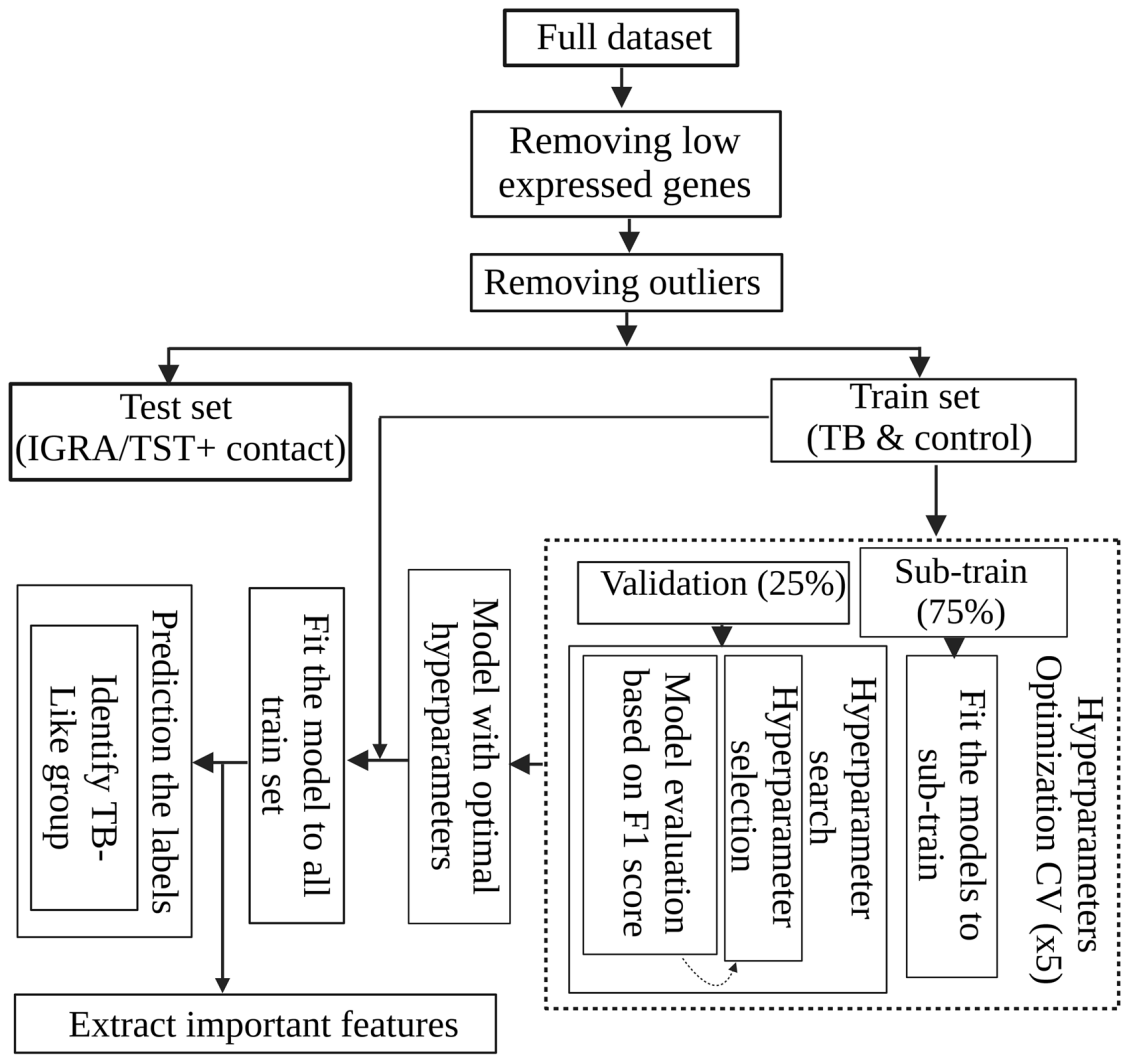
Machine learning workflow for TB classification. After standardizing the dataset and removing non-informative transcripts, the data were split into sub-train and validation sets. Decision tree-based algorithms, including random forests (RF), adaptive boosting (ADAboost), and XGBoost (XGB), were employed for binary classification to distinguish active TB and control cases based on their differentiating DEGs. The best algorithm and hyperparameters were found using a grid search with 5-fold cross-validation to maximize F-measure. The optimal model was retrained on the entire dataset and identified informative features for classification. Subsequently, the trained model was applied to IGRA/TST + contacts labeled as active TB to access the labels and identify IGRA/TST + contacts such as active TB cases.

To develop a global TB-specific signature, we conducted binary classification to discern active TB and controls using 269 differentially expressed genes. We used decision tree-based algorithms, emphasizing their robust classification capabilities and efficient parallel processing. Specifically, we used the widely adopted random forests (RFs) due to their effectiveness in handling complex datasets. We also applied adaptive boosting (ADAboost) and XGBoost (XGB) due to their proven ability to enhance classification performance through boosting techniques (Omrani et al., 2024; Freund and Schapire, 2005; Chen and Guestrin, 2016). For unbiased comparison of distinct learning algorithms and hyperparameters, we developed a grid search with a 5-fold cross-validation (CV) workflow. We first standardized the dataset by removing the mean and scaling it to unit variance using the StandardScaler function from the scikit-learn (Python v 3.10.6). This step ensured that the contribution of each transcript in the analysis is not affected by sequence depth. The data were randomly split into training (75%) and validation (25%) sets. Fine-tuning of the hyperparameters of each algorithm was conducted through an exhaustive search in a cross-validation loop, which was used for the proper evaluation of the predictive model. The combination of hyperparameters that maximized the F1 score [balanced evaluation of false positives (FP) and false negatives (FN)] was identified as optimal and subsequently applied to the algorithm. The preprocessed data, purged of non-informative transcripts and outliers, were introduced into the machine learning workflow (Figure 1). We implemented all three classification algorithms using the scikit-learn Python package. For RF, all the hyperparameters were set to default except for the n_estimators hyperparameter (the number of trees in the forest) and Max_depth (the maximum depth of each tree in the forest). Exhaustive searches for n_estimators hyperparameter were performed among 5, 10, 30, 50, 100, and 200 and for Max_depth among default, 10, 20, and 50.

For ADABoost and XGB, all the hyperparameters were set to default except for the n_estimators hyperparameter (the maximum number of weak learners to be combined in ADABoost and is the number of boosting round in XGB) and learning_rate (the contribution of each weak learner to the final combined model). We searched for the best n_estimators hyperparameter among 5, 10, 30, 50, 100, and 200 and learning_rate among default, 0.01, 0.1, 0.5, 1, and 10.

The best model, along with its optimal hyperparameters (XGB, see Table 3), was retrained on the entire dataset, encompassing all active TB and control subjects. This model was then employed on 42 IGRA/TST + contacts that were labeled as active TB to identify the most similar subjects to active TB cases.

**Table 3:** F1 score for each algorithm using the best combination of hyperparameters found by the grid search method when the models were tested with validation data.

Algorithms	Best hyperparameters	F1 score for validation set
Adaptive boosting (ADAboost)	learning_rate: 0.1, n_estimators: 200	88%
Random forest (RF)	max_depth: 10, n_estimators: 200	93%
XGBoost (XGB)	learning_rate: 0.1, n_estimators:200	95%

## 3 Results

### 3.1 Differential expression analysis to compare TB subgroups

**Figure 2:**
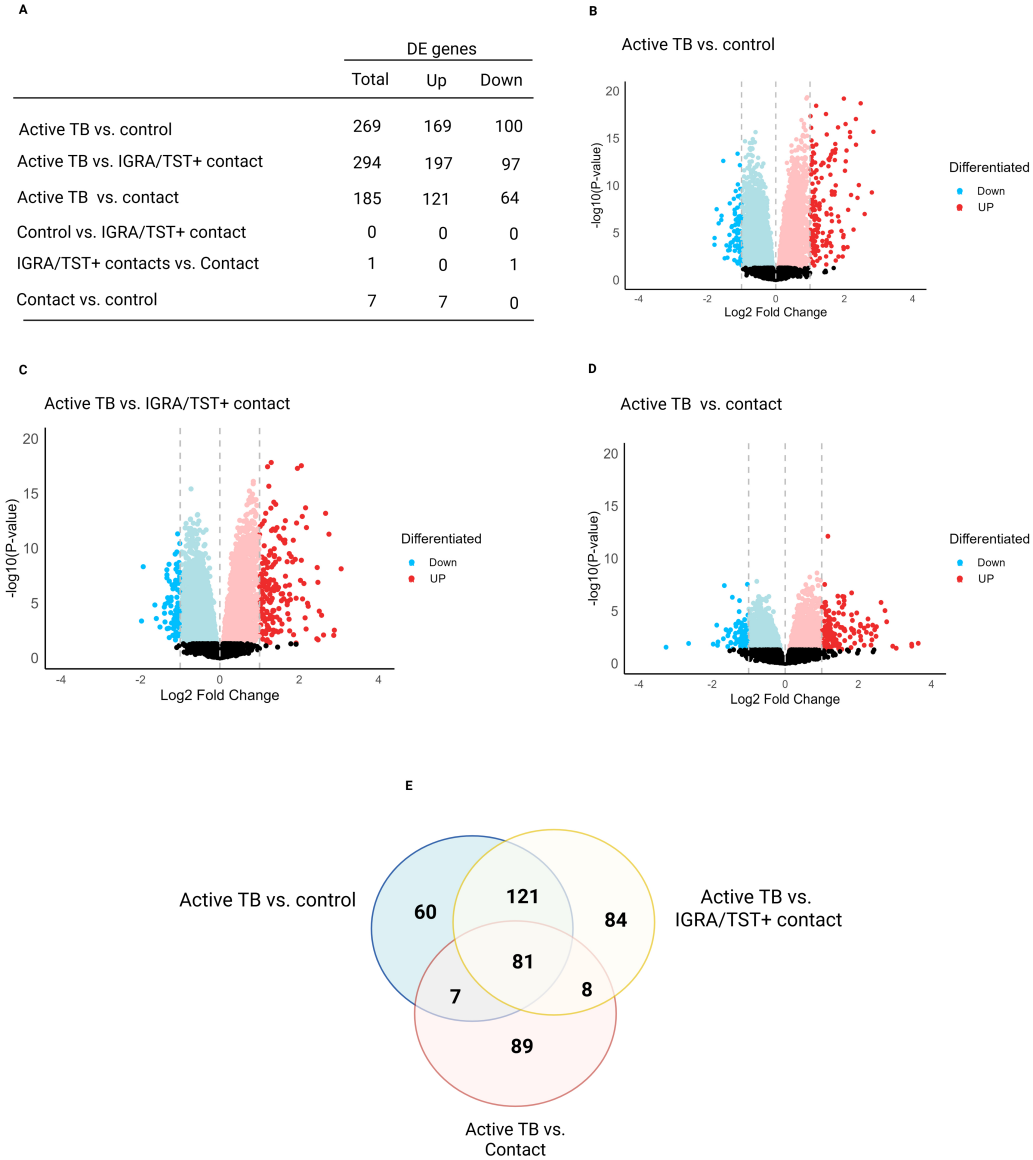
Summary of differentially expressed genes (DEGs) in tuberculosis stages. **(A)** The number of DEGs, both up- and downregulated, related to four pairwise comparisons. Volcano plots highlight the most significant genes dysregulated between **(B)** active TB and control group, **(C)** active TB and IGRA/TST + contacts, and **(D)** active TB and contacts. **(E)** A Venn diagram illustrates the overlapping TB signatures among the different comparisons.

A differential gene expression analysis was performed using pairwise comparisons between experimental groups and found 269 DE genes between ATB and control groups (Figures 2A,B; Supplementary Table S1), 294 genes between ATB and IGRA/TST + contacts (Figures 2A,C; Supplementary Table S2), 185 DE genes between ATB patients and contacts (Figures 2A,D; Supplementary Table S3), 0 DE gene between IGRA/TST + contact and controls (Figure 2A), 1 DE gene between IGRA/TST + contacts and contacts (Figure 2A; Supplementary Table S4), and 7 DE genes between contacts and control samples (Figure 2A; Supplementary Table S5).

As shown in the Venn diagram (Figure 2E), TB signatures derived from a comparison between ATB, controls, IGRA/TST + contacts, and contact subjects have 81 genes in common (Supplementary Table S6).

Since all contact populations belong to the Singaporean cohort, to obtain a comprehensive list of dysregulated genes between contacts and control individuals and mitigate potential batch effects, a differential expression gene analysis was conducted exclusively on individuals from this country. Supplementary Table S7 shows 126 DE genes obtained where only contacts and control individuals from Singapore were compared. The global ANOVA-style comparison across all experimental groups identified genes with significant differences in expression (FDR < 0.01), supporting the presence of extensive transcriptional variation across conditions. To prioritize high-confidence findings, we applied additional filters: F-statistic > 30,000 and AveExpr > 5. This resulted in a focused set of 367 genes, which are reported in Supplementary Table S8.

Pathway enrichment analysis of the 367 genes revealed enrichment in multiple immune- and signaling-related pathways. Notably, several pathways related to interleukin signaling (e.g., IL-12, TGF-beta, and interleukins), MHC antigen presentation, MAPK signaling cascades, JAK–STAT signaling, WNT and RAF kinase pathways, and viral and parasite infections (e.g., HIV, SARS-CoV, and Leishmania) were significantly represented. These results highlight that the observed transcriptional variation across experimental groups is associated with key immune regulatory and host–pathogen interaction pathways, further supporting the biological relevance of the group differences. A list of top enriched pathways is provided in Supplementary Table S9.

### 3.2 Biological processes involved in TB

**Figure 3:**
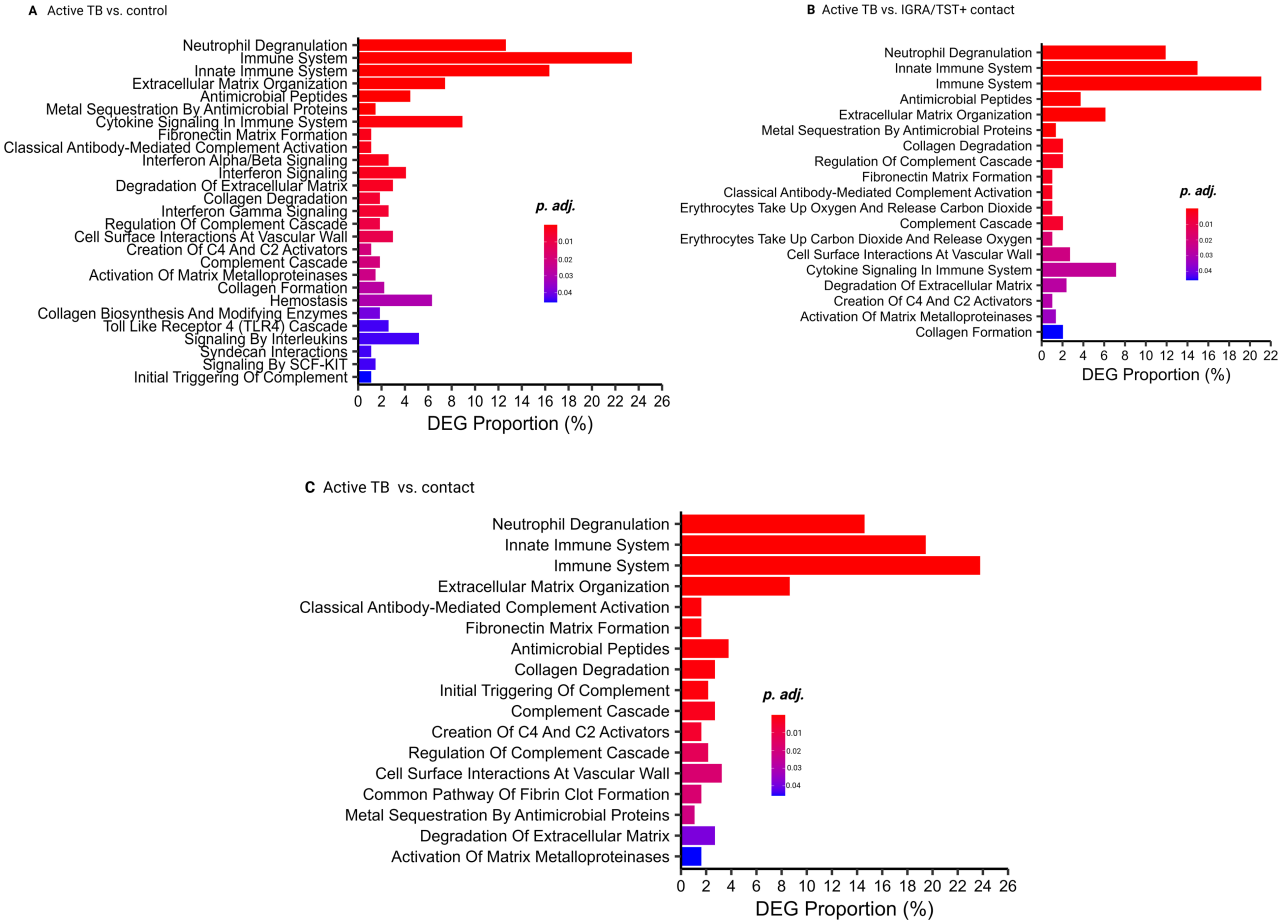
Pathway analysis. **(A)** Pathway analysis comparing active TB and control groups. **(B)** Pathway analysis comparing active TB and IGRA/TST + contacts. **(C)** Pathway analysis comparing active TB and contacts.

Pathway analyses revealed significant differences in biological processes between ATB and controls, IGRA/TST + contacts, and contacts. These pathways include neutrophil degranulation, immune system, antimicrobial peptides, complement cascade, extracellular matrix organization, and other biological processes related to host response against TB infection (Figures 3A–C) (Supplementary Tables S10–S12).

Neutrophil degranulation, immune system, and antimicrobial peptide-related genes are also significantly enriched among 81 TB common signatures (Supplementary Table S12).

Supplementary Table S13 shows exclusively biological processes involved in contacts for Singaporean participants.

### 3.3 Binary classification correctly distinguished TB patients from control subjects from different countries and reduced TB-specific signatures

To investigate whether the blood transcriptome harbors sufficient information to discern TB patients from control cases, we conducted a binary classification workflow (Figure 1). As shown in Figure 1, after preprocessing data (discarding non-informative transcripts and outlier subjects), the data containing TB patients and controls from diverse geographical regions entered an evaluation loop. During 5-fold cross-validation, the dataset was partitioned into sub-training and validation sets. Each fold involved training a model with a specific set of hyperparameters on the sub-training data and subsequently evaluating its performance using the validation data. All three algorithms with perfect performance discerned two groups. Table 3 shows the F1 score for each algorithm using the best combination of hyperparameters found by the grid search method when the models were tested with validation data. The model with the highest F1 score was chosen as the best model. This XGB-based model was then retrained on the complete training set. This approach resulted in reducing the initial set of features from 269 DE genes to 99 important features, which were then used to access the label of the IGRA/TST + contacts. The final model identified 11 of 42 (26%) of IGRA/TST + contacts (4 of 15 subjects from Mozambique and 7 of 27 subjects from Spain) with similar transcriptome profiles of TB patients (TB-like) and 31 of 42 (74%) of IGRA/TST + contacts (11 of 15 subjects from Mozambique and 20 of 27 subjects from Spain) with similar expression profiles to controls (No TB-like).

The 99 features ranked based on the prediction power using the feature_importances attribute from the sklearn package (Supplementary Table S14). Supplementary Table S15 shows pathway enrichment for 99 features.

Clustering analysis based on the 99-gene signature provided additional insights into TB subjects, the TB-like group, and contacts (see Supplementary data).

### 3.4 Gene set enrichment analysis (GSEA) discerns IGRA/TST+ contacts resembling TB patients (TB-like group)

To explore the gene enrichment patterns in the IGRA/TST+ contacts that showed similar blood profiles to TB patients (TB-like) versus those classified as No TB-like, we conducted GSEA using the H collection from the Molecular Signatures Database (MSigDB) (consisting of 50 gene sets). The results indicated that 16 gene sets were significantly upregulated in the TB-like group, with a false discovery rate (FDR) of less than 25% and a nominal *p*-value of less than 5%. The FDR threshold was selected in accordance with the GSEA user guide (Subramanian et al., 2005), which recommends this cutoff for exploratory analyses involving phenotype permutations. This level balances the risk of false positives and maintains adequate sensitivity to identify biologically relevant gene sets that merit further investigation. The significantly upregulated gene sets include those that are involved in host immune responses such as IL6_JAK_STAT3_SIGNALING, INTERFERON_GAMMA_RESPONSE, TNFA_SIGNALING_VIA_NFKB, INTERFERON_ALPHA_RESPONSE, INFLAMMATORY_RESPONSE, and COMPLEMENT (Supplementary Table S16, see gene names along with their corresponding identifiers for each gene set in Supplementary Tables S17–S22). In contrast, no gene sets were significantly enriched in the No TB-like group.

**Figure 4:**
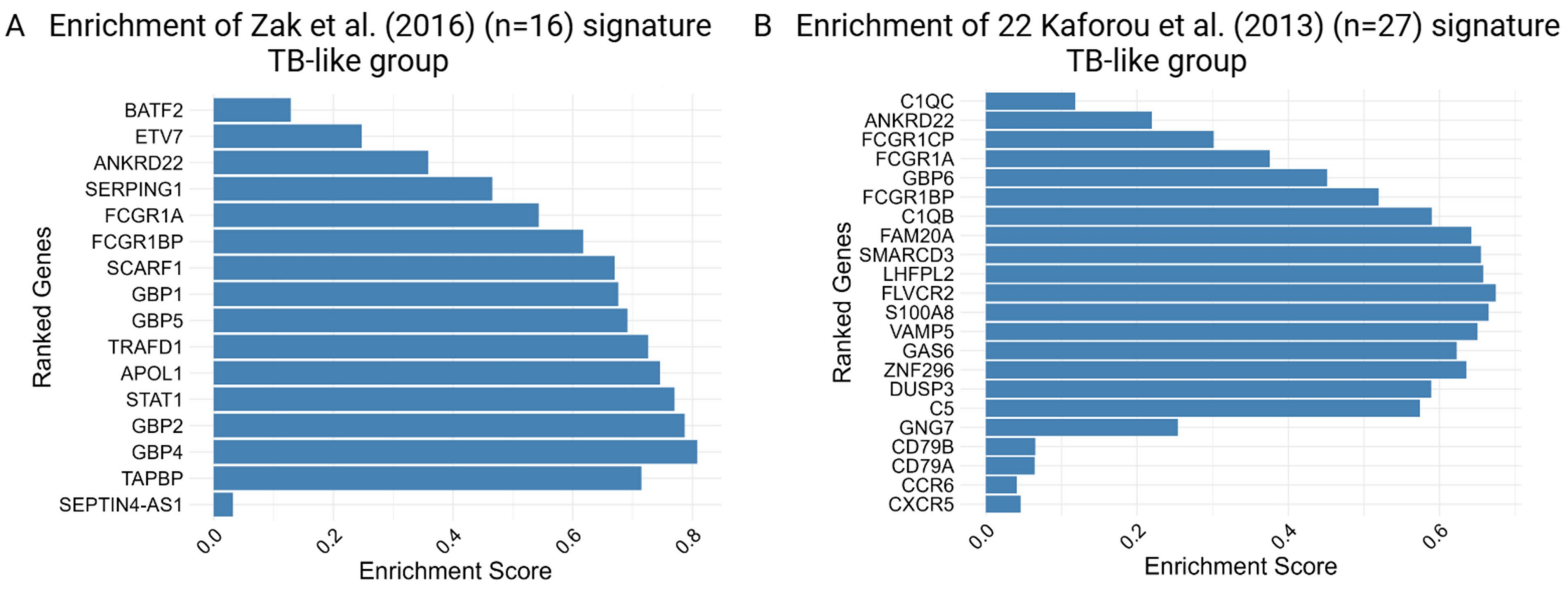
Enrichment score barplot for TB-like group obtained by gene set enrichment analysis (GSEA) using **(A)** 16 Zak et al. and **(B)** 22 out of 27 Kaforou et al. TB gene signatures. The genes are ranked from top to bottom based on their fold changes, reflecting the degree of differential expression associated with the phenotype.

Furthermore, applying the previously published 16-gene signature for progression to TB by Zak et al. (2016) and the 22-signature (out of 27) discriminating against TBI from TB by Kaforou et al. (2013) shows high enrichment for TB-like with respect to the No TB-like group, as shown in Figure 4, indicating different profiles in the two groups of IGRA/TST + contacts (TB-like vs. No TB-like). These gene sets did not show significant enrichment when contacts were compared to the controls. However, as shown in Supplementary Table S23, some genes among the 16-Zak signature were slightly upregulated in contacts with respect to controls.

### 3.5 Validation of the model and 10 top features using an independent dataset

A total of 90 TB subjects and 20 healthy controls from the publicly available dataset PRJNA352062 were subjected to differential gene expression analysis (see Methods). The analysis identified 450 differentially expressed genes, including 336 upregulated and 114 downregulated genes (Supplementary Table S24).

The dataset was preprocessed following the methodology applied to the training set to remove non-informative genes and outlier subjects. During this process, five subjects (two healthy controls and three TB patients) were identified as outliers and excluded from the study. The XGBoost algorithm was then retrained on the complete training dataset using the top 10 features (BATF2, FAM20A, FBLN2, AK5, VAMP5, MMP8, KLHDC8B, LINC00402, DEFA3, and GBP6) to reduce the number of predictive features for clinical application. The model was subsequently evaluated on the independent validation dataset.

**Figure 5:**
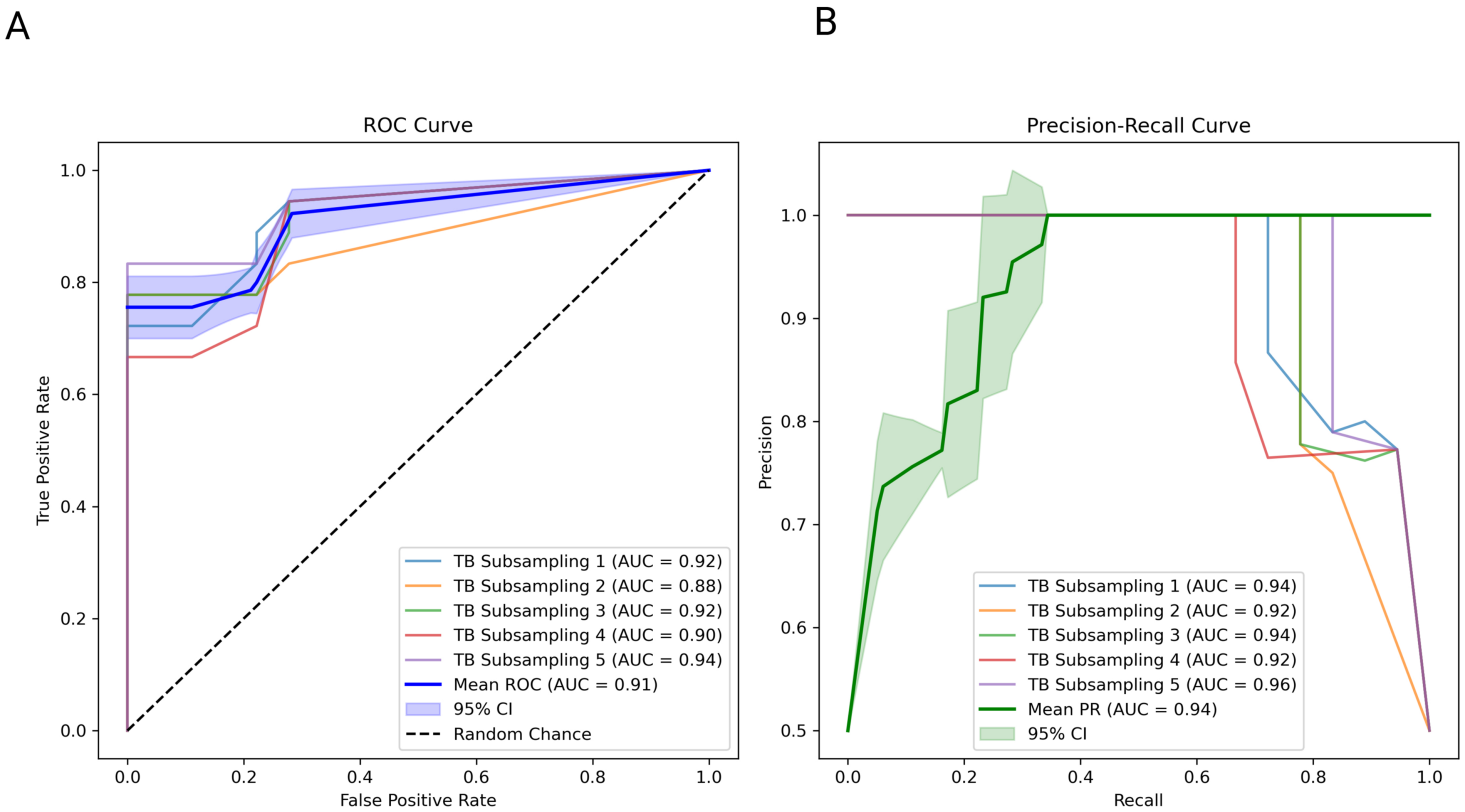
Validation of the model and top 10 features with an independent dataset. **(A)** ROC curve with confidence interval and individual iterations: The receiver operating characteristic (ROC) curves illustrate the performance of the classification model across five iterations of TB subsampling. Each curve corresponds to a subsampling, displaying the true-positive rate (TPR) against the false-positive rate (FPR). The dashed black diagonal line represents the random chance baseline. The blue curve represents the mean ROC across iterations, with the shaded blue region denoting the 95% confidence interval (CI) for the mean ROC. The area under the curve (AUC) for each iteration and the mean AUC value with 95% CI are annotated in the legend. **(B)** Precision–recall curve with confidence interval and individual iterations: The precision–recall (PR) curves evaluate the model’s precision (positive predictive value) against recall (sensitivity) for five TB subsampling iterations. Each curve represents an individual subsampling, with the green curve depicting the mean PR curve. The shaded green region highlights the 95% confidence interval (CI) for the mean PR curve. The area under the curve (AUC) for precision–recall for each iteration and the mean PR AUC with 95% CI are annotated in the legend.

To ensure robust model performance, testing was conducted across five iterations. Each iteration included all healthy control subjects and a subset of TB subjects. The results, including the ROC and precision–recall curves, along with the individual AUCs from each iteration, mean values, and 95% confidence intervals, are presented in Figure 5.

## 4 Discussion

Tuberculosis (TB) remains a global health challenge, and there is increasing interest in the accuracy and scalability of transcriptomic signatures to diagnose TB across diverse settings (Mulenga et al., 2020; Blankley et al., 2014; Singhania et al., 2018b). In this study, we utilized diverse public datasets from several countries including Spain, Mozambique, Singapore, Indonesia, and South Africa to improve the discrimination between active TB and control subjects and identify contacts with positive immunoreactivity who have TB-like blood profiles. Our initial comparisons involved TB patients, IGRA/TST + contacts, contacts, and control groups using RNA-seq analysis.

We used the term IGRA/TST + instead of the routine nomenclature “latent TB” to emphasize that a positive immune response, such as from IGRA or TST, does not necessarily indicate a TB infection. This change in terminology reflects the evolving understanding of TB pathogenesis in which immunoreactivity may indicate past exposure or infection clearance, rather than necessarily ongoing latent infection. Our gene expression and pathway analysis further confirmed this new classification. Comparing active TB patients with controls and IGRA/TST+ contacts showed that controls and contacts displayed remarkably similar blood profiles. This suggests that many of the IGRA/TST + individuals either never had an active infection or had successfully cleared it. This was further re-confirmed by labeling assessment using a trained model, which showed that only 26% of IGRA/TST + subjects were identified to have similar blood profiles to active TB (TB-like group).

Our integrative analysis of differentially expressed genes identified a 269-gene signature that distinguishes active TB from controls. This signature includes key genes such as BATF2, ANKRD22, GBP1, GBP5, FCGR1A, FCGR1BP, SEPTIN4, SERPING1, ETV7, SCARF1, GBP2, and APOL1, which align with previously reported TB-related signatures (Zak et al., 2016; Kaforou et al., 2013; Mulenga et al., 2020).

In our TB cohort, we also observed an upregulation of inflammatory markers such as S100A12, S100A8, S100A9, and RETN, which are associated with myeloid cell accumulation and inflammatory monocyte activity in TB (Blankley et al., 2014; Singhania et al., 2018b; Darboe et al., 2018; Roe et al., 2016). Furthermore, we identified an upregulation of TCN1 and TCN2, involved in cobalamin (vitamin B12) transport, suggesting that MTB may enhance its survival by increasing vitamin B12 uptake (Estévez et al., 2020). In addition, syndecans (SDC1 and SDC3), which promote bacterial internalization, were also upregulated in the TB cohort, supporting previous studies (Roe et al., 2020).

While comparisons between contacts and all controls revealed few dysregulated genes, a greater number of differentially expressed genes (ADM, IFITM2, and IFITM3) were identified when Singaporean contacts were compared with controls from the same country, likely due to the elimination of potential batch effects (Roe et al., 2016; Roe et al., 2020; Zimmermann et al., 2016; Xu et al., 2022; Pan et al., 2017).

Our results highlighted that neutrophil degranulation is a key pathway in TB patients, followed by other immune responses such as interferon signaling and cytokine signaling, which is consistent with previous studies (Estévez et al., 2020; Ranjbar et al., 2015). However, pathways involved in bacterial killing, such as neutrophil degranulation and antimicrobial peptides, were absent when comparing contacts and control subjects from Singapore. This suggests that contacts may have initiated immune responses aimed at controlling the infection but have not progressed to the later stages associated with the active elimination of replicating mycobacteria.

It has been shown that massive long non-coding RNAs (lncRNA) play several critical roles in Mtb-induced apoptosis, autophagy of macrophages, and the pathogenesis of TB. The Meg3, a significant LncRNA, exhibits downregulation when TB patients were compared to controls and IGRA/TST + contacts in the current study. The decreased expression of this lncRNA has previously been linked to key immune responses such as increased cell proliferation, reduced apoptosis, and enhanced autophagy in macrophages. Furthermore, it has been shown that the knockdown of MEG3 in macrophages resulted in the induction of autophagy and enhanced eradication of intracellular *M. bovis* BCG (Berry et al., 2010; Behar et al., 2011; Kim et al., 2019; Pawar et al., 2016; Almatroudi, 2022).

To identify more specific discriminative signatures, we performed binary classification, prioritizing differentially expressed genes between all TB patients and controls. Unlike previous studies (Estévez et al., 2020), we utilized integrated datasets, enhancing data diversity for training, validation, and label prediction. This approach aligns with real-world scenarios and supports the development of a robust, globally applicable model. Assigning feature importance in our study suggested that 99 transcripts of 269 (37%) can discriminate TB cases from controls.

The significant enrichment of gene sets involved in key immune response mechanisms, such as interferon and inflammatory responses in TB-like with respect to No TB-like, supports the hypothesis that these individuals could be at high risk of progressing to active TB. GSEA further validated the relevance of the previously identified gene signatures (by Zak and Kaforou et al.) in the TB-like group.

The observed heterogeneity within TB patients and the TB-like group, as revealed through hierarchical clustering using 99 selected transcriptomic features, offers deeper insights into the complex and diverse nature of TB progression. Interestingly, clustering analysis, which performed exclusively on TB patients, showed that some individuals exhibited an elevated expression of genes related to immune responses and antibacterial activity. These findings suggest the potential for personalized treatment strategies and deserve further investigation. In addition, MEG3 emerged as one of the most variably expressed genes across the cohort, highlighting its potential as a biomarker for monitoring TB outcomes—an area that warrants future research. Our top 10 ranked features based on their prediction power have a remarkable overlap with published TB signatures. These features were further evaluated using an independent dataset and showed robust prediction performance. This includes BATF2, which has the highest predictive score in our analysis to distinguish TB from controls (Supplementary Table S15) and is also a component of the 11-gene signature reported by Darboe et al. (2018) and the 16-gene signature by Zak et al. (2016). Moreover, BATF2 was highlighted as a potential single gene for discriminating TB from TBI in the study by Roe et al. based on its diagnostic value among different settings (Roe et al., 2016; Roe et al., 2020). This transcription factor plays a key role in TB immunopathology and is upregulated in response to interferon signaling, particularly through IFN-*γ* and interactions with IRF1, which mediate macrophage activation and inflammatory responses in TB. Its strong predictive power, highlighted by its top ranking in our analysis and inclusion in multiple published TB gene signatures, suggests that BATF2 could be a key biomarker for tracking disease progression and immune responses in TB.

FAM20A, previously reported in 25 Kaforou signatures (Mulenga et al., 2020), was identified among the top features. In contrast, FBLN2, AK5, and KLHDC8B, although not previously associated with TB, also emerged as high-scoring features. These genes have demonstrated prognostic value in other diseases, particularly various cancers, and may deserve further investigation in the context of TB (Lawrie et al., 2018). VAMP5 is also present in 25 Kaforou signatures (Kaforou et al., 2013), and MMP8 showed altered expression in the cohort with TB in an integrated dataset (Singh et al., 2024). Next is LINC00402, a long non-coding RNA that emerged as one of the top features, suggesting a potential role in the TB-related immune response. DEFA3 represents a promising biomarker in the fight against tuberculosis, offering insights into disease mechanisms and potential new avenues for diagnosis and treatment. Its role in the immune response underscores the importance of innate immunity in controlling TB infection. It provides a foundation for future research and therapeutic development (Rivas-Santiago et al., 2006), and the 10th feature is GBP6, which previously linked to TB in 25 Kaforou signatures and Rajan 5-gene signature (Mulenga et al., 2020; Rajan et al., 2019).

The identified transcriptomic signature has practical applications across multiple stages of TB management. It is valuable for screening high-risk individuals, particularly subclinical patients in endemic regions, where early detection can prevent progression and reduce transmission. These biomarkers also enable treatment monitoring, aiding clinicians in evaluating therapy effectiveness, especially in multidrug-resistant TB (MDR-TB) cases. In addition, the signature could inform prevention strategies in at-risk populations, such as those with TB infection or immunosuppression, enabling targeted interventions and efficient resource allocation.

An advantage of this study lies in the integration of multiple complementary approaches, including differential gene expression analysis, machine learning-based classification, feature selection, and unsupervised clustering. Together, these techniques enabled higher resolution characterization of TB-associated transcriptomic profiles. This integrative framework revealed subtle subgroup differences that may be overlooked using conventional gene expression analyses alone. Moreover, the relatively large and geographically diverse sample size increased statistical power, while the application of batch effect correction ensured consistency and reliability across datasets. However, relying on publicly available datasets limited access to biological samples and complete metadata such as immunoreactivity results, thereby restricting the ability to correlate findings with clinical outcomes and to perform more detailed data stratification. For instance, tracking TB-like individuals with profiles that resemble active TB could provide insights into early disease stages or progression risk. Future studies with complete metadata and longitudinal analyses are necessary to validate these findings and refine their clinical applicability.

In conclusion, our study underscores the potential of integrative blood transcriptome analysis for improving TB diagnosis and risk stratification. The identified gene signatures offer valuable candidates for further validation and development of targeted diagnostic and therapeutic interventions. By enhancing our understanding of TB pathogenesis and progression, this research contributes to the global effort to combat TB and reduce its impact on public health.

## Data Availability

Publicly available datasets were analyzed in this study. This data can be found at: “PRJEB31975” https://www.ebi.ac.uk/ena/browser/view/PRJEB31975, “PRJNA595691” https://www.ebi.ac.uk/ena/browser/view/PRJNA595691, “PRJNA798683”, https://www.ebi.ac.uk/ena/browser/view/PRJNA798683, and “PRJNA352062” https://www.ebi.ac.uk/ena/browser/view/PRJNA352062.

## Footnotes

^1^ https://www.ncbi.nlm.nih.gov/geo/
