# Supplementary material for: An integrative and comprehensive analysis of blood transcriptomes combined with machine learning models reveals key signatures for tuberculosis diagnosis and risk stratification
Source: Front Microbiol. 2025 May 26;16:1546770. doi: 10.3389/fmicb.2025.1546770 (PMC12146336; doi:10.3389/fmicb.2025.1546770)
Supplement: Supplementary file 1 [file Data_Sheet_1.pdf]

Supplementary Material

An integrative and comprehensive analysis of blood transcriptomes, coupled with machine learning models, revealed key signatures for tuberculosis diagnosis and risk stratification

Maryam Omrani[1], Arash Ghodousi[1,2], Daniela Maria Cirillo[1,2*]

[1]Emerging Bacterial Pathogens Unit, IRCCS San Raffaele Scientific Institute, Milan, Italy

[2]Università Vita Salute San Raffaele, Milan, Italy

*** Correspondence:**
Daniela Maria Cirillo, cirillo.daniela@hsr.it

Keywords: Mycobacterium tuberculosis, Biomarkers, blood, RNA-seq, Machine-learning

1 Supplementary Data

Heterogeneity among TB patients and TB-like group

To obtain a detailed transcription profile among different TB subgroups, we performed hierarchical clustering. To do so, raw reads were transformed to transcripts per kilobase million (TPM) to correct for both sequence depth and gene length. The dataset includes 11 TB_like cases, 89 ATB and all contacts. Through clustering, subjects were grouped into 4 distinct clusters (based on the Elbow method for optimal number of the cluster), to explore underlying patterns within the expression profile. Hierarchical clustering using 99 features shows heterogeneity among TB subjects (Supplementary Figure 1). Grouping the most TB-like individuals (9/11) alongside with 60% of TB cases (total 53/89 TB cases in cluster 3 and 4), confirms the similarity between TB_like and most active TB cases and aligns with the label prediction. Furthermore, grouping (cluster 2) a small group of TB subjects (11/89, 12%) and 2 (18%) TB_like cases along with all TB contacts suggests shared gene expression patterns among them and might hint at potential insights into disease progression. The number of subjects within each cluster indicates that the clustering does not reflect any source biases in the data.

To better understand which genes, play a significant role in clustering, we calculated the expression variability for the 99 genes across the four clusters. This analysis highlights genes with high variability, suggesting they may drive the differences between TB subgroups. The most variable genes include MEG3, ZNF727, BCAM, RHOXF1 and HBM indicating their potential influence in defining distinct expression patterns and contributing to the heterogeneity observed within the clusters. Supplementary Figure 2 shows the expression of 5 most variably expressed genes among 126 subjects **(**including 11 TB_like cases, 89 ATB and all contacts**).** Table S.25. shows coefficient of variability for all 99 features.

Furthermore, we also performed the same hierarchical clustering specifically for the active TB cohort, confirming that the cohort displays a heterogeneous profile, particularly in terms of immune responses and antibacterial activity (Supplementary Figure 3). Part of active TB patients characterized by higher expression in different interferon signaling (cluster 1 & cluster 2)

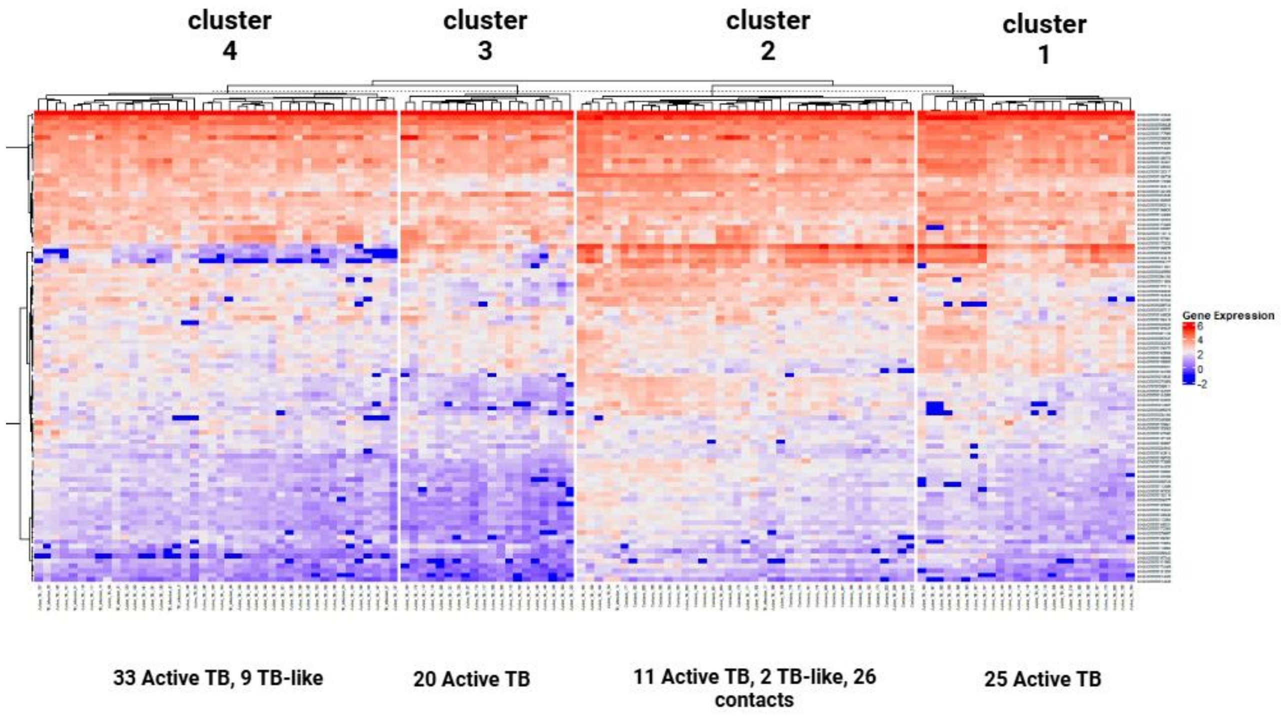

Supplementary Figure1. Hierarchical clustering heatmap of gene expression profiles across different tuberculosis (TB) sample groups. Clustering anlysis identified four distinct clusters based on gene expression patterns of 99 features. Cluster 1 consists of 25 active TB cases, Cluster 2 includes 11 active TB, 2 TB-like, and 26 contacts, Cluster 3 contains 20 active TB cases, and Cluster 4 consists of 33 active TB and 9 TB-like cases. Each row represents a gene, and each column represents a sample, with red indicating higher expression levels and blue indicating lower expression levels.

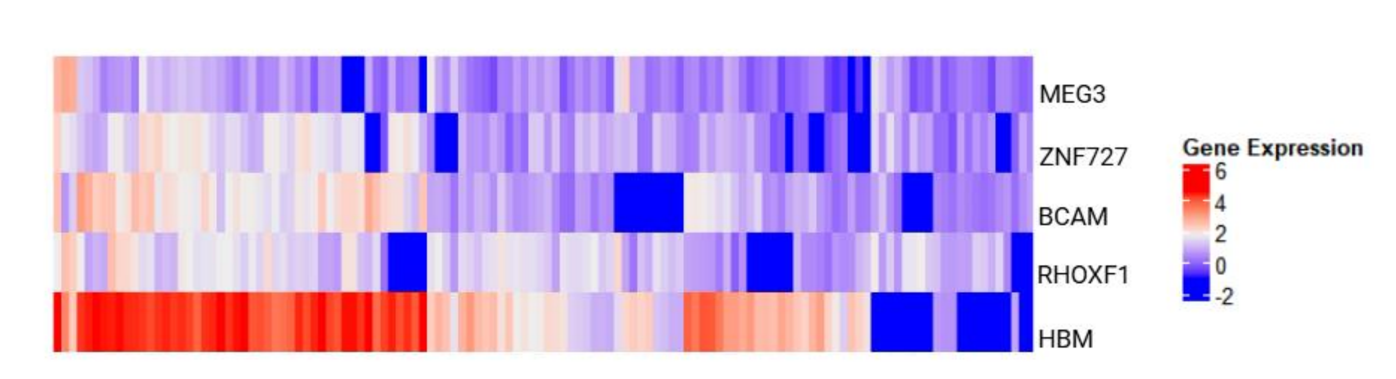

Supplementary Figure2. Heatmap of gene expression profiles for 5 most variably expressed genes among 4 clusters in **Supplementary Figure1.**

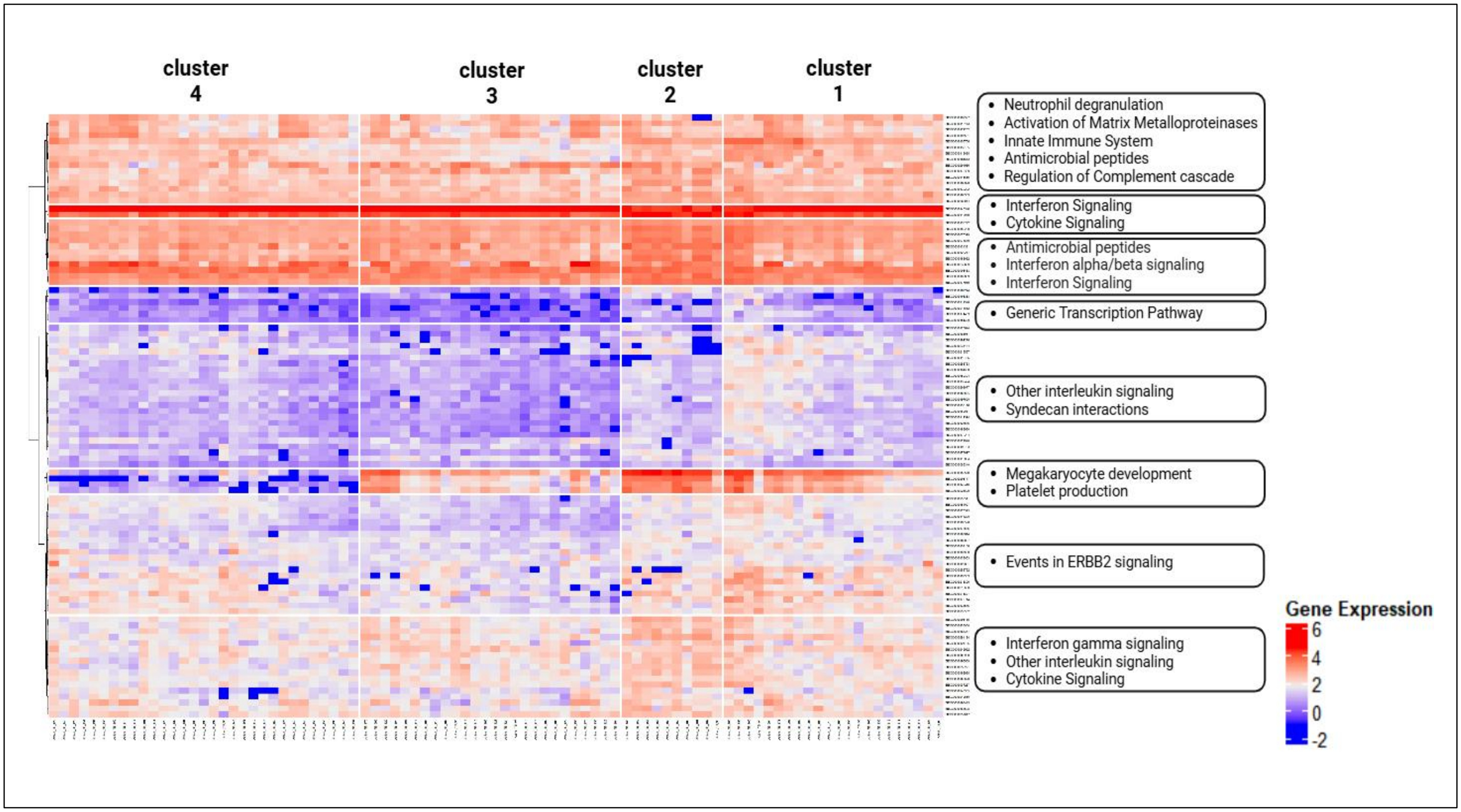

Supplementary Figure3. Hierarchical clustering heatmap of gene expression profiles for active TB patients, using 99 selected features. The heatmap reveals heterogeneity among TB patients, particularly in genes related to immune response and antibacterial activity, suggesting varying levels of immune system engagement and pathogen control mechanisms across individuals.

Table S.1. 269 Differentially Expressed (DE) genes between active TB patients and controls

Ensemble ID	Gene Symbol	Log2FC	adj.p value
ENSG00000108950	FAM20A	1,988145	3,38E-16
ENSG00000168062	BATF2	2,479234	7,98E-16
ENSG00000082014	SMARCD3	1,178452	1,16E-15
ENSG00000074660	SCARF1	1,465833	7,27E-15
ENSG00000162645	GBP2	1,014961	1,04E-14
ENSG00000152766	ANKRD22	2,339423	1,84E-14
ENSG00000150337	FCGR1A	2,038333	4,55E-14
ENSG00000154451	GBP5	1,817699	8,68E-14
ENSG00000075399	VPS9D1	1,032166	8,85E-14
ENSG00000159189	C1QC	2,850926	2,00E-13
ENSG00000197646	PDCD1LG2	2,16739	2,00E-13
ENSG00000291135	FCGR1BP	1,488807	3,34E-13
ENSG00000183347	GBP6	2,169536	4,96E-13
ENSG00000087237	CETP	1,042314	9,70E-13
ENSG00000214872	SMTNL1	1,737351	1,89E-12
ENSG00000177989	ODF3B	1,182806	2,10E-12
ENSG00000173369	C1QB	2,342662	2,15E-12
ENSG00000141574	SECTM1	1,063933	2,55E-12
ENSG00000143546	S100A8	1,059045	2,93E-12
ENSG00000152229	PSTPIP2	1,0872	3,04E-12
ENSG00000233030	LOC124904411	1,747783	4,59E-12
ENSG00000010030	ETV7	2,066042	5,75E-12
ENSG00000233029	LOC100996318	1,660517	6,08E-12
ENSG00000100342	APOL1	1,091997	8,62E-12
ENSG00000120217	CD274	1,729498	8,87E-12
ENSG00000145685	LHFPL2	1,115945	1,09E-11
ENSG00000290735	Novel transcript	2,202264	1,26E-11
ENSG00000183762	KREMEN1	1,618583	1,28E-11
ENSG00000163220	S100A9	1,043814	2,17E-11
ENSG00000117228	GBP1	1,590699	2,32E-11
ENSG00000198959	TGM2	1,541574	3,29E-11
ENSG00000108387	SEPTIN4	1,724384	3,93E-11
ENSG00000139572	GPR84	1,561692	3,93E-11
ENSG00000185909	KLHDC8B	1,062013	4,61E-11
ENSG00000149131	SERPING1	2,043169	5,08E-11
ENSG00000157551	KCNJ15	1,14875	6,35E-11
ENSG00000163568	AIM2	1,369962	6,52E-11

ENSG00000198216	CAC-1E	1,393605	7,05E-11
ENSG00000135636	DYSF	1,044648	9,05E-11
ENSG00000119686	FLVCR2	1,046105	9,95E-11
ENSG00000188820	CALHM6	1,325233	1,02E-10
ENSG00000100985	MMP9	1,53773	1,29E-10
ENSG00000168899	VAMP5	1,020152	2,69E-10
ENSG00000163221	S100A12	1,189157	2,82E-10
ENSG00000265531	FCGR1CP	2,019909	3,11E-10
ENSG00000255221	CARD17P	1,800664	3,88E-10
ENSG00000079385	CEACAM1	1,126322	4,39E-10
ENSG00000116663	FBXO6	1,035365	6,41E-10
ENSG00000163958	ZDHHC19	1,595497	6,83E-10
ENSG00000166278	C2	1,338232	1,32E-09
ENSG00000148926	ADM	1,176397	1,34E-09
ENSG00000112053	SLC26A8	1,242676	3,30E-09
ENSG00000185338	SOCS1	1,070222	4,29E-09
ENSG00000170439	TMT1B	2,42529	5,65E-09
ENSG00000173372	C1QA	1,750604	5,92E-09
ENSG00000225492	GBP1P1	2,193552	6,81E-09
ENSG00000284194	SCO2	1,157717	1,38E-08
ENSG00000079393	DUSP13	1,635953	1,96E-08
ENSG00000106070	GRB10	1,065151	2,13E-08
ENSG00000130202	NECTIN2	1,720877	2,20E-08
ENSG00000185339	TCN2	1,109035	2,24E-08
ENSG00000204936	CD177	2,805604	2,45E-08
ENSG00000100336	APOL4	1,71398	3,74E-08
ENSG00000226004	SIMALR	1,639775	3,74E-08
ENSG00000283646	LINC02009	2,197951	4,02E-08
ENSG00000136231	IGF2BP3	1,121861	5,49E-08
ENSG00000118113	MMP8	2,386803	6,84E-08
ENSG00000223914	LINC02471	1,169808	8,67E-08
ENSG00000267293	LOC100422497	1,057872	1,01E-07
ENSG00000142089	IFITM3	1,391389	1,15E-07
ENSG00000260943	LINC02555	1,190352	1,22E-07
ENSG00000102010	BMX	1,154603	1,47E-07
ENSG00000184557	SOCS3	1,073317	1,87E-07
ENSG00000163814	CDCP1	1,278232	2,06E-07
ENSG00000174944	P2RY14	1,193059	3,00E-07
ENSG00000139832	RAB20	1,040661	3,17E-07
ENSG00000070729	CNGB1	1,280425	3,51E-07
ENSG00000162512	SDC3	1,105594	3,62E-07
ENSG00000249992	TMEM158	1,265504	3,87E-07
ENSG00000289582	Novel transcript	1,470584	4,70E-07
ENSG00000257017	HP	1,602319	5,67E-07
ENSG00000133106	EPSTI1	1,275194	6,25E-07

ENSG00000138772	ANXA3	1,044766	6,45E-07
ENSG00000012223	LTF	2,113173	7,19E-07
ENSG00000183019	MCEMP1	1,137149	7,46E-07
ENSG00000178814	OPLAH	1,072153	7,76E-07
ENSG00000005961	ITGA2B	1,014997	1,18E-06
ENSG00000135424	ITGA7	1,482093	1,35E-06
ENSG00000235321	Novel transcript	1,524628	1,50E-06
ENSG00000288836	Novel transcript	1,472737	1,63E-06
ENSG00000196415	PRTN3	2,595404	1,67E-06
ENSG00000164047	CAMP	1,620803	1,80E-06
ENSG00000129682	FGF13	1,489226	1,83E-06
ENSG00000073737	DHRS9	1,001584	1,87E-06
ENSG00000008438	PGLYRP1	1,126352	2,34E-06
ENSG00000101335	MYL9	1,17004	2,40E-06
ENSG00000148346	LCN2	1,817711	2,66E-06
ENSG00000136514	RTP4	1,051647	2,84E-06
ENSG00000158089	GALNT14	1,218217	4,24E-06
ENSG00000112299	VNN1	1,129933	5,30E-06
ENSG00000186583	SPATC1	1,264954	5,49E-06
ENSG00000115884	SDC1	1,638924	6,85E-06
ENSG00000005381	MPO	1,846774	7,36E-06
ENSG00000167994	RAB3IL1	1,13998	1,42E-05
ENSG00000130222	GADD45G	1,195978	1,45E-05
ENSG00000143416	SELENBP1	1,255523	1,68E-05
ENSG00000004939	SLC4A1	1,226815	2,94E-05
ENSG00000167105	TMEM92	1,209538	3,00E-05
ENSG00000197561	ELANE	2,270617	3,81E-05
ENSG00000255328	Novel transcript	1,162081	3,89E-05
ENSG00000121236	TRIM6	1,014665	3,94E-05
ENSG00000157168	NRG1	1,138366	4,15E-05
ENSG00000124469	CEACAM8	1,917336	4,92E-05
ENSG00000270972	Novel transcript	1,073962	5,65E-05
ENSG00000124785	NRN1	1,628582	5,65E-05
ENSG00000206177	HBM	1,050536	5,96E-05
ENSG00000101425	BPI	1,586134	6,19E-05
ENSG00000223609	HBD	1,234631	6,77E-05
ENSG00000158578	ALAS2	1,372357	7,50E-05
ENSG00000134827	TCN1	1,084308	9,36E-05
ENSG00000088340	FER1L4	1,049425	0,000112
ENSG00000172232	AZU1	1,978815	0,000115
ENSG00000167100	SAMD14	1,156216	0,000131
ENSG00000096006	CRISP3	1,548847	0,000145
ENSG00000119917	IFIT3	1,036391	0,000147
ENSG00000187244	BCAM	1,034016	0,000158

ENSG00000250138	LOC728488	1,188441	0,000164
ENSG00000288853	Novel transcript	1,492339	0,000171
ENSG00000086548	CEACAM6	1,969959	0,000176
ENSG00000133742	CA1	1,081794	0,000211
ENSG00000168528	SERINC2	1,023298	0,000214
ENSG00000069535	MAOB	1,486132	0,000322
ENSG00000157554	ERG	1,360313	0,000384
ENSG00000183856	IQGAP3	1,144597	0,000399
ENSG00000129173	E2F8	1,024646	0,000426
ENSG00000274173	LINC02967	1,216398	0,000501
ENSG00000173391	OLR1	1,581457	0,000593
ENSG00000248385	TARM1	1,475783	0,000625
ENSG00000064270	ATP2C2	1,06042	0,000633
ENSG00000198753	PLXNB3	1,053537	0,000932
ENSG00000224940	PRRT4	1,371238	0,000979
ENSG00000242550	SERPINB10	1,138989	0,000993
ENSG00000065618	COL17A1	1,471689	0,000998
ENSG00000179869	ABCA13	1,625981	0,001099
ENSG00000156113	KCNMA1	1,206865	0,001216
ENSG00000133063	CHIT1	1,186179	0,001307
ENSG00000164821	DEFA4	1,975106	0,001345
ENSG00000087116	ADAMTS2	2,095363	0,001361
ENSG00000102837	OLFM4	1,982576	0,001997
ENSG00000287576	Novel transcript	1,030452	0,002064
ENSG00000118520	ARG1	1,098324	0,002094
ENSG00000185897	FFAR3	1,012332	0,002354
ENSG00000167680	SEMA6B	1,240784	0,002883
ENSG00000134321	RSAD2	1,168225	0,003203
ENSG00000163710	PCOLCE2	1,606273	0,003676
ENSG00000122861	PLAU	1,085313	0,004091
ENSG00000166033	HTRA1	1,031447	0,004893
ENSG00000213275	IFITM9P	1,158545	0,006998
ENSG00000170801	HTRA3	1,469531	0,009219
ENSG00000100448	CTSG	1,952675	0,009433
ENSG00000239839	DEFA3	1,830739	0,009542
ENSG00000165949	IFI27	1,286841	0,013067
ENSG00000162949	CAPN13	1,009992	0,014543
ENSG00000104918	RETN	1,01918	0,016706
ENSG00000231535	LINC00278	1,751114	0,021497
ENSG00000122641	INHBA	1,211512	0,022916
ENSG00000129538	RNASE1	1,086673	0,027762
ENSG00000204710	SPDYC	1,066943	0,0377
ENSG00000169397	RNASE3	1,474813	0,048945
ENSG00000128536	CDHR3	-1,11617	1,11E-11
ENSG00000275395	FCGBP	-1,5341	4,61E-11

ENSG00000153064	BANK1	-1,04811	1,03E-10
ENSG00000156738	MS4A1	-1,10808	4,85E-09
ENSG00000184613	NELL2	-1,03769	1,20E-08
ENSG00000135318	NT5E	-1,17026	1,84E-08
ENSG00000144645	OSBPL10	-1,04868	4,55E-08
ENSG00000132185	FCRLA	-1,06227	5,09E-08
ENSG00000196092	PAX5	-1,10641	5,09E-08
ENSG00000103528	SYT17	-1,03115	6,55E-08
ENSG00000176928	GCNT4	-1,01348	7,46E-08
ENSG00000278897	Novel transcript	-1,15783	7,91E-08
ENSG00000164330	EBF1	-1,22558	8,27E-08
ENSG00000197385	ZNF860	-1,34414	2,04E-07
ENSG00000111728	ST8SIA1	-1,0231	2,47E-07
ENSG00000133424	LARGE1	-1,00017	2,59E-07
ENSG00000196172	ZNF681	-1,03065	3,74E-07
ENSG00000152894	PTPRK	-1,12514	4,72E-07
ENSG00000182379	NXPH4	-1,72745	6,34E-07
ENSG00000173585	CCR9	-1,20308	7,11E-07
ENSG00000113088	GZMK	-1,19324	1,28E-06
ENSG00000126838	PZP	-1,19649	1,70E-06
ENSG00000172264	MACROD2	-1,58407	2,34E-06
ENSG00000235621	LINC00494	-1,03259	2,47E-06
ENSG00000186231	KLHL32	-1,0808	2,70E-06
ENSG00000026559	KCNG1	-1,01442	2,70E-06
ENSG00000260400	Novel transcript	-1,09492	3,14E-06
ENSG00000185567	AHNAK2	-1,12417	4,17E-06
ENSG00000005471	ABCB4	-1,06073	4,20E-06
ENSG00000265590	CFAP298-TCP10L	-1,00252	4,22E-06
ENSG00000163520	FBLN2	-1,16812	5,25E-06
ENSG00000120057	SFRP5	-1,59219	5,46E-06
ENSG00000235532	LINC00402	-1,04181	6,38E-06
ENSG00000249667	LINC01259	-1,11016	1,04E-05
ENSG00000174469	CNT-P2	-1,6687	1,08E-05
ENSG00000163629	PTPN13	-1,01352	1,17E-05
ENSG00000151320	AKAP6	-1,0679	1,32E-05
ENSG00000082293	COL19A1	-1,17447	1,32E-05
ENSG00000112394	SLC16A10	-1,08666	1,37E-05
ENSG00000154027	AK5	-1,0067	1,46E-05
ENSG00000183691	NOG	-1,19049	1,55E-05
ENSG00000183960	KCNH8	-1,2778	1,89E-05
ENSG00000002745	WNT16	-1,29005	2,29E-05
ENSG00000211725	TRBV5-5	-1,11958	4,02E-05
ENSG00000286330	LOC105370259	-1,03184	4,96E-05

ENSG00000153253	SCN3A	-1,21738	5,26E-05
ENSG00000197520	FAM177B	-1,09308	5,30E-05
ENSG00000162630	B3GALT2	-1,13682	6,83E-05
ENSG00000100095	SEZ6L	-1,09768	7,74E-05
ENSG00000286010	LOC105375754	-1,13082	7,93E-05
ENSG00000255733	IFNG-AS1	-1,35397	0,000111
ENSG00000198046	ZNF667	-1,15071	0,000188
ENSG00000211821	TRDV2	-1,78747	0,000209
ENSG00000155970	MICU3	-1,11025	0,000242
ENSG00000258511	LINC02295	-1,08236	0,000244
ENSG00000123454	DBH	-1,02507	0,000247
ENSG00000211806	TRAV25	-1,05272	0,000251
ENSG00000184226	PCDH9	-1,11318	0,000267
ENSG00000144290	SLC4A10	-1,21507	0,0003
ENSG00000169031	COL4A3	-1,12845	0,000304
ENSG00000214652	ZNF727	-1,12136	0,00034
ENSG00000257275	Novel Transcript	-1,08318	0,000384
ENSG00000213557	RPL31P43	-1,26432	0,00048
ENSG00000275772	Novel transcript	-1,29065	0,000482
ENSG00000290592	LOC107985211	-1,01373	0,000501
ENSG00000163687	DNASE1L3	-1,30823	0,000531
ENSG00000173114	LRRN3	-1,31943	0,000593
ENSG00000134545	KLRC1	-1,0125	0,000727
ENSG00000091129	NRCAM	-1,79067	0,000833
ENSG00000215630	GUSBP9	-1,03249	0,000934
ENSG00000112486	CCR6	-1,12758	0,000944
ENSG00000135116	HRK	-1,43934	0,000947
ENSG00000206077	ZDHHC11B	-1,19474	0,001043
ENSG00000175894	TSPEAR	-1,33902	0,001252
ENSG00000101883	RHOXF1	-1,00557	0,001453
ENSG00000182389	CACNB4	-1,10955	0,001556
ENSG00000143184	XCL1	-1,02127	0,001866
ENSG00000167601	AXL	-1,05811	0,002118
ENSG00000289976	Novel transcript	-1,14878	0,002178
ENSG00000289278	Novel transcript	-1,02238	0,002217
ENSG00000166342	NETO1	-1,01442	0,002334
ENSG00000175746	LINC02915	-1,3472	0,002472
ENSG00000106341	PPP1R17	-1,39069	0,002681
ENSG00000233392	UICLM	-1,19778	0,002862
ENSG00000289545	LINC03063	-1,02485	0,003558
ENSG00000211713	TRBV6-4	-1,05913	0,003574
ENSG00000258732	Novel transcript	-1,09898	0,004963
ENSG00000288933	Novel transcript	-1,1351	0,00528
ENSG00000275158	TRBV12-5	-1,00579	0,008487
ENSG00000152672	CLEC4F	-1,41286	0,011877

ENSG00000234184	LINC01781	-1,03059	0,012031
ENSG00000214548	MEG3	-1,49281	0,013559
ENSG00000197353	LYPD2	-1,03138	0,016813
ENSG00000160307	S100B	-1,21122	0,01967
ENSG00000054179	ENTPD2	-1,04762	0,022841
ENSG00000175445	LPL	-1,13871	0,027428
ENSG00000226321	CROCC2	-1,03605	0,029039
ENSG00000276070	CCL4L2	-1,02665	0,037843
ENSG00000186081	KRT5	-1,11038	0,039658
ENSG00000288861	Novel transcript	-1,0837	0,0421

Table S.2. 294 Differentially Expressed (DE) genes between active TB patients and IGRA/TST+ contacts

Ensemble ID	Gene Symbol	Log2FC	adj.p value
ENSG00000143546	S100A8	1,293888	1,92E-14
ENSG00000139572	GPR84	2,05376	1,92E-14
ENSG00000082014	SMARCD3	1,202185	1,92E-14
ENSG00000108950	FAM20A	1,954561	2,04E-14
ENSG00000163220	S100A9	1,233867	4,95E-13
ENSG00000074660	SCARF1	1,359904	5,49E-12
ENSG00000163221	S100A12	1,407469	7,77E-12
ENSG00000152766	ANKRD22	2,159655	1,40E-11
ENSG00000157551	KCNJ15	1,285533	1,43E-11
ENSG00000185909	KLHDC8B	1,155405	3,53E-11
ENSG00000159189	C1QC	2,664419	3,53E-11
ENSG00000168062	BATF2	2,076398	5,48E-11
ENSG00000183762	KREMEN1	1,650125	1,16E-10
ENSG00000135636	DYSF	1,119261	1,16E-10
ENSG00000197646	PDCD1LG2	1,932954	1,61E-10
ENSG00000180061	TMEM150B	1,067141	1,76E-10
ENSG00000141574	SECTM1	1,015546	2,68E-10
ENSG00000173369	C1QB	2,182195	3,22E-10
ENSG00000198216	CAC-1E	1,439309	3,22E-10
ENSG00000100985	MMP9	1,609403	3,31E-10
ENSG00000111199	TRPV4	1,339856	3,71E-10
ENSG00000163568	AIM2	1,396038	4,49E-10
ENSG00000148926	ADM	1,300265	4,58E-10
ENSG00000255221	CARD17P	1,914124	4,98E-10
ENSG00000170439	TMT1B	2,749501	8,18E-10
ENSG00000268500	SIGLEC5	1,001662	8,76E-10
ENSG00000198959	TGM2	1,488099	1,16E-09
ENSG00000154451	GBP5	1,518465	1,54E-09

ENSG00000108387	SEPTIN4	1,638649	2,13E-09
ENSG00000183347	GBP6	1,85017	2,16E-09
ENSG00000120217	CD274	1,597233	2,21E-09
ENSG00000150337	FCGR1A	1,635124	3,32E-09
ENSG00000079385	CEACAM1	1,117566	5,34E-09
ENSG00000102010	BMX	1,369714	8,60E-09
ENSG00000112053	SLC26A8	1,273896	1,26E-08
ENSG00000290735	Novel transcript	1,918882	1,48E-08
ENSG00000225492	GBP1P1	2,286264	1,53E-08
ENSG00000284194	SCO2	1,279496	1,68E-08
ENSG00000010030	ETV7	1,749474	1,85E-08
ENSG00000134755	DSC2	1,242304	2,38E-08
ENSG00000183019	MCEMP1	1,378906	2,53E-08
ENSG00000149131	SERPING1	1,797308	3,02E-08
ENSG00000117228	GBP1	1,37248	3,03E-08
ENSG00000197272	IL27	1,063569	3,28E-08
ENSG00000121797	CCRL2	1,124431	4,37E-08
ENSG00000163958	ZDHHC19	1,492291	4,41E-08
ENSG00000291135	FCGR1BP	1,139421	4,60E-08
ENSG00000257017	HP	1,900929	4,66E-08
ENSG00000158578	ALAS2	2,053735	5,45E-08
ENSG00000173372	C1QA	1,695295	7,08E-08
ENSG00000004939	SLC4A1	1,833553	7,36E-08
ENSG00000233030	LOC124904411	1,401036	7,65E-08
ENSG00000138772	ANXA3	1,209657	7,77E-08
ENSG00000139832	RAB20	1,15799	8,98E-08
ENSG00000106070	GRB10	1,070701	9,17E-08
ENSG00000233029	LOC100996318	1,351393	1,11E-07
ENSG00000223914	LINC02471	1,237934	1,19E-07
ENSG00000260943	LINC02555	1,289909	1,35E-07
ENSG00000214872	SMTNL1	1,307003	1,66E-07
ENSG00000118113	MMP8	2,453055	1,83E-07
ENSG00000166278	C2	1,202754	1,85E-07
ENSG00000196415	PRTN3	3,055958	2,22E-07
ENSG00000188820	CALHM6	1,099783	2,64E-07
ENSG00000178814	OPLAH	1,158285	5,18E-07
ENSG00000185339	TCN2	1,041135	5,42E-07
ENSG00000167434	CA4	1,054884	6,81E-07
ENSG00000223552	CCR5AS	1,021573	7,38E-07
ENSG00000129682	FGF13	1,652925	7,78E-07
ENSG00000005961	ITGA2B	1,076293	1,29E-06
ENSG00000101335	MYL9	1,274171	1,39E-06
ENSG00000079215	SLC1A3	1,111646	1,42E-06
ENSG00000158089	GALNT14	1,353693	1,50E-06
ENSG00000283646	LINC02009	2,031001	1,56E-06

ENSG00000229644	NAMPTP1	1,169388	1,76E-06
ENSG00000204936	CD177	2,470672	2,17E-06
ENSG00000255328	Novel transcript	1,477373	2,18E-06
ENSG00000070729	CNGB1	1,240817	2,25E-06
ENSG00000249992	TMEM158	1,235275	2,25E-06
ENSG00000123610	TNFAIP6	1,11567	2,41E-06
ENSG00000073737	DHRS9	1,049452	2,55E-06
ENSG00000012223	LTF	2,117976	2,63E-06
ENSG00000112299	VNN1	1,23534	2,80E-06
ENSG00000079393	DUSP13	1,411819	2,96E-06
ENSG00000223609	HBD	1,754309	3,40E-06
ENSG00000184792	OSBP2	1,096301	3,46E-06
ENSG00000280832	GSEC	1,00672	3,50E-06
ENSG00000143416	SELENBP1	1,652499	4,08E-06
ENSG00000162551	ALPL	1,005157	4,75E-06
ENSG00000174944	P2RY14	1,122612	4,77E-06
ENSG00000186583	SPATC1	1,36327	4,81E-06
ENSG00000121236	TRIM6	1,214816	5,01E-06
ENSG00000163814	CDCP1	1,169518	5,23E-06
ENSG00000100336	APOL4	1,478359	5,28E-06
ENSG00000157168	NRG1	1,342181	6,00E-06
ENSG00000265531	FCGR1CP	1,455687	7,44E-06
ENSG00000230257	NFE4	1,181857	7,95E-06
ENSG00000130222	GADD45G	1,300915	8,58E-06
ENSG00000226004	SIMALR	1,368107	8,99E-06
ENSG00000123342	MMP19	1,054942	1,00E-05
ENSG00000169877	AHSP	1,581391	1,53E-05
ENSG00000164047	CAMP	1,54492	1,59E-05
ENSG00000008438	PGLYRP1	1,084526	1,64E-05
ENSG00000148346	LCN2	1,756925	1,69E-05
ENSG00000161640	SIGLEC11	1,261431	1,72E-05
ENSG00000270972	Novel transcript	1,247627	1,79E-05
ENSG00000136514	RTP4	1,020787	1,84E-05
ENSG00000288836	Novel transcript	1,365685	2,38E-05
ENSG00000133106	EPSTI1	1,134907	2,55E-05
ENSG00000234436	Novel transcript	1,249076	2,64E-05
ENSG00000289927	Novel transcript	1,10567	3,19E-05
ENSG00000248385	TARM1	1,921794	3,33E-05
ENSG00000187244	BCAM	1,319181	3,37E-05
ENSG00000167994	RAB3IL1	1,185953	3,53E-05
ENSG00000288853	Novel transcript	1,734566	3,61E-05
ENSG00000133742	CA1	1,608533	3,61E-05
ENSG00000124469	CEACAM8	2,083661	3,73E-05
ENSG00000132170	PPARG	1,301073	4,02E-05

ENSG00000231233	CFAP58-DT	1,048121	4,20E-05
ENSG00000005381	MPO	1,778634	4,22E-05
ENSG00000135424	ITGA7	1,296187	4,92E-05
ENSG00000115884	SDC1	1,550977	5,12E-05
ENSG00000101425	BPI	1,694561	5,62E-05
ENSG00000167105	TMEM92	1,240311	5,79E-05
ENSG00000130202	NECTIN2	1,266324	6,21E-05
ENSG00000124785	NRN1	1,704358	6,56E-05
ENSG00000142089	IFITM3	1,095219	6,57E-05
ENSG00000289582	Novel transcript	1,207998	6,79E-05
ENSG00000167100	SAMD14	1,269988	6,94E-05
ENSG00000137757	CASP5	1,014153	7,05E-05
ENSG00000086548	CEACAM6	2,209165	7,96E-05
ENSG00000069535	MAOB	1,695987	0,000102
ENSG00000174705	SH3PXD2B	1,021296	0,000102
ENSG00000197561	ELANE	2,226607	0,000132
ENSG00000274173	LINC02967	1,402378	0,000152
ENSG00000122861	PLAU	1,487407	0,000196
ENSG00000162433	AK4	1,005889	0,000207
ENSG00000134827	TCN1	1,086883	0,000213
ENSG00000119917	IFIT3	1,073126	0,000214
ENSG00000106565	TMEM176B	1,337126	0,000269
ENSG00000206177	HBM	1,36132	0,000273
ENSG00000087116	ADAMTS2	2,505406	0,00031
ENSG00000242550	SERPINB10	1,33479	0,00032
ENSG00000004809	SLC22A16	1,184358	0,000326
ENSG00000155659	VSIG4	1,033284	0,000368
ENSG00000198753	PLXNB3	1,189913	0,000398
ENSG00000158352	SHROOM4	1,026123	0,000474
ENSG00000173391	OLR1	1,711003	0,000489
ENSG00000189127	ANKRD34B	1,051603	0,000541
ENSG00000158163	DZIP1L	1,093686	0,000576
ENSG00000239839	DEFA3	2,563352	0,000639
ENSG00000157856	DRC1	1,275522	0,000649
ENSG00000235321	Novel transcript	1,117817	0,000657
ENSG00000163898	LIPH	1,068964	0,000731
ENSG00000096006	CRISP3	1,436632	0,000876
ENSG00000002933	TMEM176A	1,339845	0,000884
ENSG00000164821	DEFA4	2,14737	0,001003
ENSG00000188487	INSC	1,12644	0,001037
ENSG00000166947	EPB42	1,028604	0,001098
ENSG00000118520	ARG1	1,225641	0,001161
ENSG00000143595	AQP10	1,017771	0,001175
ENSG00000157554	ERG	1,309749	0,00122
ENSG00000172232	AZU1	1,744152	0,001266

ENSG00000065618	COL17A1	1,507494	0,001417
ENSG00000254789	Novel transcript	1,472246	0,001685
ENSG00000204010	IFIT1B	1,132806	0,001779
ENSG00000167680	SEMA6B	1,33977	0,002294
ENSG00000179869	ABCA13	1,565801	0,002904
ENSG00000099725	PRKY	2,546785	0,003438
ENSG00000185897	FFAR3	1,030621	0,003447
ENSG00000287576	Novel transcript	1,025603	0,003636
ENSG00000243273	LOC124909446	1,193606	0,003675
ENSG00000277856	Novel transcript	1,107686	0,004173
ENSG00000224940	PRRT4	1,223762	0,0054
ENSG00000103723	AP3B2	1,206107	0,006043
ENSG00000231535	LINC00278	2,172553	0,007032
ENSG00000123838	C4BPA	1,250173	0,007581
ENSG00000129538	RNASE1	1,35154	0,00861
ENSG00000183878	UTY	2,884952	0,008688
ENSG00000213275	IFITM9P	1,173995	0,009189
ENSG00000170801	HTRA3	1,538016	0,009781
ENSG00000102837	OLFM4	1,75945	0,009838
ENSG00000165949	IFI27	1,412773	0,01008
ENSG00000134321	RSAD2	1,075753	0,010689
ENSG00000163710	PCOLCE2	1,471589	0,011875
ENSG00000002726	AOC1	1,357119	0,012003
ENSG00000130176	CNN1	1,024463	0,01249
ENSG00000123689	G0S2	1,157738	0,015862
ENSG00000104918	RETN	1,069121	0,017302
ENSG00000100448	CTSG	1,857166	0,019243
ENSG00000122641	INHBA	1,309754	0,019336
ENSG00000012817	KDM5D	2,612509	0,02016
ENSG00000198692	EIF1AY	2,869503	0,021414
ENSG00000249173	LINC01093	1,001047	0,022963
ENSG00000129824	RPS4Y1	2,443555	0,039327
ENSG00000289548	LOC105376995	1,082982	0,041883
ENSG00000173262	SLC2A14	1,193929	0,04648
ENSG00000067048	DDX3Y	2,474838	0,047266
ENSG00000128536	CDHR3	-1,06412	7,97E-10
ENSG00000153064	BANK1	-1,00991	3,61E-09
ENSG00000159958	TNFRSF13C	-1,08392	1,45E-08
ENSG00000156738	MS4A1	-1,12661	1,98E-08
ENSG00000184613	NELL2	-1,02916	8,07E-08
ENSG00000132185	FCRLA	-1,09718	1,04E-07
ENSG00000275395	FCGBP	-1,28148	1,06E-07
ENSG00000182379	NXPH4	-1,93148	1,60E-07
ENSG00000144645	OSBPL10	-1,06036	1,61E-07

ENSG00000196092	PAX5	-1,1068	2,37E-07
ENSG00000185567	AHNAK2	-1,33571	2,62E-07
ENSG00000111728	ST8SIA1	-1,0636	3,75E-07
ENSG00000103528	SYT17	-1,01662	3,85E-07
ENSG00000249667	LINC01259	-1,33343	6,16E-07
ENSG00000132704	FCRL2	-1,01355	7,88E-07
ENSG00000197385	ZNF860	-1,33426	1,02E-06
ENSG00000128218	VPREB3	-1,01438	1,15E-06
ENSG00000235621	LINC00494	-1,11444	1,55E-06
ENSG00000171798	KNDC1	-1,27403	1,77E-06
ENSG00000164330	EBF1	-1,12676	2,53E-06
ENSG00000288598	LOC105370174	-1,03291	2,55E-06
ENSG00000113088	GZMK	-1,20511	3,35E-06
ENSG00000227388	Novel transcript	-1,00914	3,45E-06
ENSG00000235532	LINC00402	-1,12785	3,64E-06
ENSG00000278897	Novel transcript	-1,0326	4,36E-06
ENSG00000126838	PZP	-1,18246	6,62E-06
ENSG00000170899	GSTA4	-1,06668	7,77E-06
ENSG00000226945	RPL13AP8	-1,00365	7,98E-06
ENSG00000227354	RBM26-AS1	-1,00476	1,24E-05
ENSG00000289278	Novel transcript	-1,52662	1,61E-05
ENSG00000205056	LINC02397	-1,03593	2,11E-05
ENSG00000151320	AKAP6	-1,08401	2,84E-05
ENSG00000100095	SEZ6L	-1,22711	2,88E-05
ENSG00000183960	KCNH8	-1,31549	3,05E-05
ENSG00000233387	IATPR	-1,05751	3,36E-05
ENSG00000286010	LOC105375754	-1,22798	5,03E-05
ENSG00000253364	COPDA1	-1,22519	6,67E-05
ENSG00000169031	COL4A3	-1,3115	7,07E-05
ENSG00000211710	TRBV4-1	-1,05564	7,59E-05
ENSG00000154764	WNT7A	-1,03906	9,18E-05
ENSG00000172264	MACROD2	-1,37794	9,34E-05
ENSG00000233392	UICLM	-1,63809	0,000106
ENSG00000002745	WNT16	-1,23354	0,000123
ENSG00000286330	LOC105370259	-1,02055	0,000141
ENSG00000082293	COL19A1	-1,07645	0,000147
ENSG00000255733	IFNG-AS1	-1,40054	0,000152
ENSG00000260306	Novel transcript	-1,01438	0,000196
ENSG00000143869	GDF7	-1,1137	0,000269
ENSG00000258732	Novel transcript	-1,48992	0,000277
ENSG00000123454	DBH	-1,0729	0,00028
ENSG00000255569	TRAV1-1	-1,02479	0,000298
ENSG00000114948	ADAM23	-1,23805	0,000405
ENSG00000197520	FAM177B	-1,00632	0,00044
ENSG00000163687	DNASE1L3	-1,39567	0,000449

ENSG00000101883	RHOXF1	-1,13425	0,000626
ENSG00000280119	LOC285097	-1,01562	0,000626
ENSG00000228495	LINC01013	-1,0583	0,000649
ENSG00000275772	Novel transcript	-1,31329	0,000738
ENSG00000288782	Novel transcript	-1,19943	0,00104
ENSG00000226321	CROCC2	-1,60128	0,001129
ENSG00000184226	PCDH9	-1,04875	0,001134
ENSG00000112486	CCR6	-1,15738	0,001246
ENSG00000155970	MICU3	-1,02307	0,001382
ENSG00000214548	MEG3	-1,9746	0,001725
ENSG00000103647	CORO2B	-1,00923	0,001879
ENSG00000144290	SLC4A10	-1,10168	0,001911
ENSG00000197353	LYPD2	-1,38291	0,002037
ENSG00000133661	SFTPD	-1,06615	0,002081
ENSG00000213557	RPL31P43	-1,16965	0,002226
ENSG00000167601	AXL	-1,10909	0,002229
ENSG00000074527	NTN4	-1,11621	0,002304
ENSG00000174469	CNT-P2	-1,21845	0,002454
ENSG00000168913	ENHO	-1,02012	0,003093
ENSG00000206077	ZDHHC11B	-1,12637	0,003342
ENSG00000261655	Novel transcript	-1,04789	0,003342
ENSG00000275158	TRBV12-5	-1,14528	0,004193
ENSG00000289263	Novel transcript	-1,14703	0,004611
ENSG00000175445	LPL	-1,50894	0,00496
ENSG00000211816	TRAV38-1	-1,14222	0,004988
ENSG00000182389	CACNB4	-1,02233	0,005779
ENSG00000175746	LINC02915	-1,28759	0,006149
ENSG00000234184	LINC01781	-1,17586	0,006317
ENSG00000106341	PPP1R17	-1,32695	0,006653
ENSG00000254275	LINC00824	-1,28301	0,007509
ENSG00000289976	Novel transcript	-1,05699	0,007541
ENSG00000250321	Novel transcript	-1,12168	0,008685
ENSG00000227242	Novel transcript	-1,66972	0,011808
ENSG00000165810	BTNL9	-1,10661	0,011811
ENSG00000225544	Novel transcript	-1,13679	0,014832
ENSG00000135116	HRK	-1,1018	0,016954
ENSG00000134765	DSC1	-1,02566	0,017966
ENSG00000169435	RASSF6	-1,01153	0,020564
ENSG00000211821	TRDV2	-1,18462	0,021297
ENSG00000158748	HTR6	-1,15161	0,023257
ENSG00000249307	LINC01088	-1,0548	0,023261
ENSG00000152672	CLEC4F	-1,27517	0,031992
ENSG00000186081	KRT5	-1,11847	0,04808

Table S.3. 185 Differentially Expressed (DE) genes between active TB patients and contacts

Ensemble ID	Gene Symbol	Log2FC	adj.p value
ENSG00000038427	VCAN	1,167557	1,21E-08
ENSG00000198734	F5	1,080837	5,48E-05
ENSG00000290735	Novel transcript	2,298109	6,29E-05
ENSG00000108950	FAM20A	1,816997	0,000137
ENSG00000291135	FCGR1BP	1,438762	0,000213
ENSG00000233029	LOC100996318	1,569402	0,000213
ENSG00000102010	BMX	1,608438	0,000216
ENSG00000139572	GPR84	1,613825	0,000319
ENSG00000145685	LHFPL2	1,10398	0,000381
ENSG00000283646	LINC02009	2,621382	0,000389
ENSG00000119686	FLVCR2	1,081939	0,000391
ENSG00000285938	LOC122526776	1,467429	0,000391
ENSG00000289331	Novel transcript	1,342067	0,00045
ENSG00000100985	MMP9	1,606384	0,000508
ENSG00000185499	MUC1	1,110315	0,000577
ENSG00000159339	PADI4	1,015405	0,000736
ENSG00000233030	LOC124904411	1,558538	0,000736
ENSG00000290021	Novel transcript	1,206135	0,000944
ENSG00000274290	H2BC6	1,07501	0,001038
ENSG00000164124	TMEM144	1,033286	0,001084
ENSG00000118113	MMP8	2,72935	0,001106
ENSG00000163221	S100A12	1,194037	0,001112
ENSG00000183762	KREMEN1	1,463372	0,001581
ENSG00000257017	HP	1,957379	0,001609
ENSG00000198216	CACNA1E	1,329203	0,00166
ENSG00000079385	CEACAM1	1,113756	0,001676
ENSG00000150337	FCGR1A	1,591349	0,001853
ENSG00000152766	ANKRD22	1,812801	0,001991
ENSG00000258227	CLEC5A	1,122699	0,002054
ENSG00000163568	AIM2	1,203194	0,002903
ENSG00000287576	Novel transcript	2,039096	0,002956
ENSG00000096006	CRISP3	2,264417	0,003517
ENSG00000165626	BEND7	1,261082	0,003746
ENSG00000150760	DOCK1	1,339561	0,003751
ENSG00000273295	Novel transcript	2,593756	0,003803
ENSG00000265531	FCGR1CP	1,839061	0,004218
ENSG00000079215	SLC1A3	1,171272	0,004218
ENSG00000112053	SLC26A8	1,190359	0,004441
ENSG00000138119	MYOF	1,07191	0,004669
ENSG00000196415	PRTN3	2,775703	0,004985

ENSG00000282988	Novel transcript	1,087133	0,006155
ENSG00000158089	GALNT14	1,434755	0,006344
ENSG00000273812	LINC02970	1,170886	0,006483
ENSG00000154451	GBP5	1,241024	0,006913
ENSG00000101425	BPI	2,05739	0,006913
ENSG00000267293	LOC100422497	1,182615	0,007036
ENSG00000255221	CARD17P	1,545377	0,007446
ENSG00000257743	MGAM2	1,158852	0,007449
ENSG00000124469	CEACAM8	2,393087	0,007586
ENSG00000198829	SUCNR1	1,183494	0,00775
ENSG00000173391	OLR1	2,262279	0,008152
ENSG00000005381	MPO	2,061119	0,008266
ENSG00000108387	SEPTIN4	1,378141	0,008321
ENSG00000138772	ANXA3	1,094211	0,008808
ENSG00000204936	CD177	2,496859	0,009058
ENSG00000183347	GBP6	1,509618	0,009635
ENSG00000129682	FGF13	1,558994	0,009736
ENSG00000012223	LTF	2,161144	0,010461
ENSG00000120217	CD274	1,234557	0,010795
ENSG00000183019	MCEMP1	1,121991	0,010859
ENSG00000157554	ERG	1,8103	0,010981
ENSG00000242550	SERPINB10	1,617828	0,011614
ENSG00000254554	ADM-DT	1,215382	0,01269
ENSG00000086548	CEACAM6	2,447248	0,0127
ENSG00000112299	VNN1	1,2139	0,01297
ENSG00000164047	CAMP	1,607516	0,013116
ENSG00000143545	RAB13	1,04979	0,013212
ENSG00000159189	C1QC	1,829436	0,013332
ENSG00000148346	LCN2	1,870345	0,013332
ENSG00000115884	SDC1	1,634415	0,014562
ENSG00000163958	ZDHHC19	1,167354	0,014659
ENSG00000166278	C2	1,002265	0,015059
ENSG00000164850	GPER1	1,12794	0,015267
ENSG00000288836	Novel transcript	1,478877	0,01549
ENSG00000123342	MMP19	1,049334	0,015507
ENSG00000277157	H4C4	1,14697	0,016127
ENSG00000065618	COL17A1	2,015613	0,016225
ENSG00000134827	TCN1	1,257007	0,016932
ENSG00000179869	ABCA13	2,221218	0,017454
ENSG00000186583	SPATC1	1,27542	0,01831
ENSG00000088340	FER1L4	1,247426	0,019106
ENSG00000211662	IGLV3-21	1,276854	0,01943
ENSG00000117228	GBP1	1,069724	0,01943
ENSG00000170439	TMT1B	1,881548	0,019559

ENSG00000134247	PTGFRN	1,034003	0,020536
ENSG00000197646	PDCD1LG2	1,31646	0,020591
ENSG00000008438	PGLYRP1	1,088149	0,020786
ENSG00000197561	ELANE	2,35101	0,021197
ENSG00000010030	ETV7	1,357391	0,022248
ENSG00000289927	Novel transcript	1,086778	0,022316
ENSG00000211947	IGHV3-21	1,053221	0,023038
ENSG00000211952	IGHV4-28	1,188608	0,023308
ENSG00000253214	LOC105375924	1,231226	0,02483
ENSG00000173369	C1QB	1,485721	0,025703
ENSG00000211895	IGHA1	1,178399	0,027007
ENSG00000234389	Novel transcript	1,554611	0,027094
ENSG00000163814	CDCP1	1,065773	0,027927
ENSG00000172232	AZU1	2,06172	0,02969
ENSG00000164821	DEFA4	2,484093	0,029797
ENSG00000168062	BATF2	1,232732	0,031746
ENSG00000124785	NRN1	1,77664	0,032696
ENSG00000174705	SH3PXD2B	1,006795	0,033383
ENSG00000145555	MYO10	1,172344	0,033386
ENSG00000012817	KDM5D	4,309644	0,033634
ENSG00000100336	APOL4	1,279351	0,034272
ENSG00000282651	Novel transcript	3,04345	0,035715
ENSG00000196747	H2AC13	1,33173	0,037915
ENSG00000105048	TNNT1	1,235127	0,039146
ENSG00000173372	C1QA	1,195737	0,041752
ENSG00000235321	Novel transcript	1,365271	0,041956
ENSG00000211649	IGLV7-46	1,14838	0,043412
ENSG00000274173	LINC02967	1,391317	0,043455
ENSG00000217275	RPS10P1	1,017299	0,043489
ENSG00000158874	APOA2	1,214952	0,044644
ENSG00000070729	CNGB1	1,155962	0,045345
ENSG00000253998	IGKV2-29	1,30669	0,045716
ENSG00000135424	ITGA7	1,181456	0,046687
ENSG00000229314	ORM1	1,393524	0,047169
ENSG00000116299	ELAPOR1	1,145441	0,04718
ENSG00000102837	OLFM4	2,37276	0,04718
ENSG00000163710	PCOLCE2	2,069559	0,048337
ENSG00000197540	GZMM	-1,03512	5,48E-05
ENSG00000103067	ESRP2	-1,66503	5,51E-05
ENSG00000290124	Novel transcript	-2,90082	6,29E-05
ENSG00000287218	Novel transcript	-2,17112	8,79E-05
ENSG00000288598	LOC105370174	-1,44617	0,000231
ENSG00000198711	SSBP3-AS1	-1,25034	0,000319
ENSG00000262312	Novel transcript	-1,0937	0,000596
ENSG00000228655	Novel transcript	-1,63128	0,000935

ENSG00000240291	Novel transcript	-1,06761	0,001006
ENSG00000183837	PNMA3	-1,26038	0,001297
ENSG00000003249	DBNDD1	-1,02047	0,002065
ENSG00000290003	Novel transcript	-1,4933	0,002616
ENSG00000282980	Novel transcript	-1,54315	0,002929
ENSG00000242258	LINC00996	-1,11084	0,003829
ENSG00000218713	Novel transcript	-1,78422	0,004521
ENSG00000264577	Novel transcript	-1,04814	0,004669
ENSG00000239961	LILRA4	-1,27883	0,004697
ENSG00000241666	Novel transcript	-1,12479	0,005479
ENSG00000279801	LOC112268198	-1,28848	0,005648
ENSG00000160408	ST6GAL-C6	-1,19827	0,005747
ENSG00000287920	Novel transcript	-1,0884	0,005747
ENSG00000130300	PLVAP	-1,18104	0,006657
ENSG00000126838	PZP	-1,3536	0,006674
ENSG00000174469	CNTNAP2	-1,87269	0,007563
ENSG00000237248	LINC00987	-1,0915	0,007586
ENSG00000233609	RPL10P19	-1,03052	0,007827
ENSG00000286061	RCAN3AS	-1,15654	0,008321
ENSG00000142233	NTN5	-1,18717	0,008546
ENSG00000243544	RN7SL172P	-1,34554	0,008581
ENSG00000179841	AKAP5	-1,05853	0,010086
ENSG00000249667	LINC01259	-1,22405	0,011161
ENSG00000214279	SCART1	-1,01408	0,011326
ENSG00000120057	SFRP5	-1,61991	0,01269
ENSG00000291300	PRSS30P	-1,49574	0,012886
ENSG00000162366	PDZK1IP1	-1,01584	0,013614
ENSG00000227218	LOC124902280	-1,09438	0,014359
ENSG00000070915	SLC12A3	-1,20564	0,014598
ENSG00000121753	ADGRB2	-1,02196	0,015038
ENSG00000225756	DBH-AS1	-1,08242	0,016038
ENSG00000158050	DUSP2	-1,23074	0,01688
ENSG00000286010	LOC105375754	-1,26592	0,0169
ENSG00000285994	Novel transcript	-1,29461	0,017277
ENSG00000170128	GPR25	-1,03868	0,017528
ENSG00000223519	KIF28P	-1,26679	0,018171
ENSG00000283361	CFAP97D2	-1,54458	0,018187
ENSG00000232273	FTH1P1	-1,29513	0,019658
ENSG00000214402	LCNL1	-1,359	0,019793
ENSG00000233493	TMEM238	-1,17461	0,020536
ENSG00000287855	Novel transcript	-1,20202	0,024898
ENSG00000183691	NOG	-1,11154	0,027724
ENSG00000163687	DNASE1L3	-1,56802	0,027903
ENSG00000288782	Novel transcript	-1,46072	0,028006

ENSG00000168350	DEGS2	-1,01745	0,028006
ENSG00000123454	DBH	-1,18629	0,029774
ENSG00000074527	NTN4	-1,38413	0,032628
ENSG00000163520	FBLN2	-1,02122	0,033516
ENSG00000287195	Novel transcript	-1,22366	0,036566
ENSG00000260992	DOCK9-DT	-1,04588	0,040468
ENSG00000197353	LYPD2	-1,69178	0,041246
ENSG00000111249	CUX2	-1,23883	0,043455
ENSG00000291038	Novel transcript	-1,14364	0,04388
ENSG00000002745	WNT16	-1,13701	0,045142
ENSG00000198756	COLGALT2	-1,04203	0,045208
ENSG00000142627	EPHA2	-1,32623	0,045963

Table S.4. 1 Differentially Expressed (DE) genes between IGRA/TST+ contacts and contacts

Ensemble ID	Gene Symbol	Log2FC	adj.p value
ENSG00000206177	HBM	-1,97890	0,015

Table S.5. 7 Differentially Expressed (DE) genes between contacts and control

Ensemble ID	symbol	logFC	Adj.P.Val
ENSG00000206177	HBM	1,668120	0,002376473
ENSG00000198892	SHISA4	1,588714	0,008095215
ENSG00000198336	MYL4	1,549660	0,008095215
ENSG00000290124	Novel transcript	2,51769199	0,008095215
ENSG00000218713	Novel transcript	1,97522	0,019607175
ENSG00000290003	Novel transcript	1,47092	0,033299276
ENSG00000260592	LOC124903659	1,158578	0,04741881

Table S.6. 81 Common TB signatures

Ensemble ID	Gene Symbol
ENSG00000108950	FAM20A
ENSG00000168062	BATF2
ENSG00000152766	ANKRD22
ENSG00000150337	FCGR1A
ENSG00000154451	GBP5
ENSG00000159189	C1QC
ENSG00000197646	PDCD1LG2
ENSG00000291135	FCGR1BP
ENSG00000183347	GBP6
ENSG00000173369	C1QB
ENSG00000233030	LOC124904411
ENSG00000010030	ETV7
ENSG00000233029	LOC100996318
ENSG00000120217	CD274
ENSG00000290735	Novel transcript
ENSG00000183762	KREMEN1
ENSG00000117228	GBP1
ENSG00000108387	SEPTIN4
ENSG00000139572	GPR84
ENSG00000163568	AIM2
ENSG00000198216	CAC-1E
ENSG00000100985	MMP9
ENSG00000163221	S100A12
ENSG00000265531	FCGR1CP
ENSG00000255221	CARD17P
ENSG00000079385	CEACAM1
ENSG00000163958	ZDHHC19
ENSG00000166278	C2
ENSG00000112053	SLC26A8
ENSG00000170439	TMT1B
ENSG00000173372	C1QA
ENSG00000204936	CD177
ENSG00000100336	APOL4
ENSG00000283646	LINC02009
ENSG00000118113	MMP8
ENSG00000102010	BMX
ENSG00000163814	CDCP1
ENSG00000070729	CNGB1
ENSG00000257017	HP
ENSG00000138772	ANXA3
ENSG00000012223	LTF

ENSG00000183019	MCEMP1
ENSG00000135424	ITGA7
ENSG00000235321	Novel transcript
ENSG00000288836	Novel transcript
ENSG00000196415	PRTN3
ENSG00000164047	CAMP
ENSG00000129682	FGF13
ENSG00000008438	PGLYRP1
ENSG00000148346	LCN2
ENSG00000158089	GALNT14
ENSG00000112299	VNN1
ENSG00000186583	SPATC1
ENSG00000115884	SDC1
ENSG00000005381	MPO
ENSG00000197561	ELANE
ENSG00000124469	CEACAM8
ENSG00000124785	NRN1
ENSG00000101425	BPI
ENSG00000134827	TCN1
ENSG00000172232	AZU1
ENSG00000096006	CRISP3
ENSG00000086548	CEACAM6
ENSG00000157554	ERG
ENSG00000274173	LINC02967
ENSG00000173391	OLR1
ENSG00000242550	SERPINB10
ENSG00000065618	COL17A1
ENSG00000179869	ABCA13
ENSG00000164821	DEFA4
ENSG00000102837	OLFM4
ENSG00000287576	Novel transcript
ENSG00000163710	PCOLCE2
ENSG00000126838	PZP
ENSG00000249667	LINC01259
ENSG00000174469	CNT-P2
ENSG00000002745	WNT16
ENSG00000286010	LOC105375754
ENSG00000123454	DBH
ENSG00000163687	DNASE1L3
ENSG00000197353	LYPD2

Table S.7: 126 Differentially Expressed (DE) genes between contacts and control from Singapore

Ensemble ID	Gene Symbol	Log2FC	adj,p value
ENSG00000185201	IFITM2	1,315963	0,009918
ENSG00000172243	CLEC7A	1,040638	0,011184
ENSG00000170956	CEACAM3	1,130902	0,012745
ENSG00000173110	HSPA6	1,15896	0,012745
ENSG00000182782	HCAR2	1,198444	0,012988
ENSG00000128383	APOBEC3A	1,239007	0,013079
ENSG00000069399	BCL3	1,026242	0,013351
ENSG00000157551	KCNJ15	1,222651	0,013426
ENSG00000171049	FPR2	1,108152	0,015081
ENSG00000171051	FPR1	1,027122	0,015081
ENSG00000235111	Novel transcript	1,435766	0,015295
ENSG00000275437	Novel transcript	1,701007	0,015625
ENSG00000163464	CXCR1	1,000412	0,015764
ENSG00000167578	RAB4B	1,138061	0,015764
ENSG00000167968	DNASE1L2	1,718157	0,015861
ENSG00000290124	Novel transcript	3,72521	0,016565
ENSG00000142089	IFITM3	1,65308	0,016801
ENSG00000171236	LRG1	1,306472	0,016985
ENSG00000182885	ADGRG3	1,14006	0,017211
ENSG00000159588	CCDC17	1,229365	0,017697
ENSG00000105711	SCN1B	1,331058	0,017697
ENSG00000139890	REM2	1,090236	0,01852
ENSG00000225528	TMA7B	1,119542	0,019864
ENSG00000204584	Novel transcript	1,179351	0,020876
ENSG00000105835	NAMPT	1,00394	0,02208
ENSG00000162747	FCGR3B	1,1712	0,022588
ENSG00000279801	LOC112268198	1,586021	0,02279
ENSG00000175003	SLC22A1	1,482477	0,022929
ENSG00000204420	MPIG6B	1,111171	0,024562
ENSG00000176788	BASP1	1,002301	0,024798
ENSG00000126262	FFAR2	1,005929	0,024988
ENSG00000113140	SPARC	1,033333	0,024988
ENSG00000101335	MYL9	1,890374	0,024993
ENSG00000269246	Novel transcript	1,724514	0,025341
ENSG00000236200	Novel transcript	1,431953	0,025543
ENSG00000272037	Novel transcript	1,001136	0,025543
ENSG00000223551	TMSB4XP4	1,250878	0,02565
ENSG00000005961	ITGA2B	1,200853	0,027069

ENSG00000279861	Novel transcript	1,103661	0,027407
ENSG00000093134	VNN3P	1,073148	0,027454
ENSG00000136689	IL1RN	1,081532	0,02753
ENSG00000287631	Novel transcript	1,279575	0,028301
ENSG00000286159	Novel transcript	1,435522	0,028301
ENSG00000120885	CLU	1,028656	0,028648
ENSG00000151726	ACSL1	1,031123	0,028842
ENSG00000123610	TNFAIP6	1,578652	0,031017
ENSG00000283403	LOC100420587	2,212157	0,031054
ENSG00000115590	IL1R2	1,076168	0,032523
ENSG00000268938	Novel transcript	1,529907	0,033005
ENSG00000226891	LINC01359	1,085995	0,033736
ENSG00000167676	PLIN4	1,249135	0,034498
ENSG00000101460	MAP1LC3A	1,042556	0,035317
ENSG00000163354	DCST2	1,025959	0,035317
ENSG00000225217	HSPA7	1,227015	0,035739
ENSG00000275371	Novel transcript	1,012722	0,035967
ENSG00000196358	NTNG2	1,136716	0,035967
ENSG00000135114	OASL	1,445147	0,036699
ENSG00000279884	Novel transcript	1,181899	0,039148
ENSG00000185507	IRF7	1,010925	0,039183
ENSG00000290003	Novel transcript	1,614667	0,039629
ENSG00000290958	Novel transcript	1,220772	0,039748
ENSG00000148926	ADM	1,51153	0,040473
ENSG00000169704	GP9	1,234233	0,040506
ENSG00000236409	Novel transcript	1,263872	0,041215
ENSG00000163736	PPBP	1,430206	0,041233
ENSG00000161911	TREML1	1,222375	0,041269
ENSG00000233791	BTG2-DT	1,525863	0,041337
ENSG00000167549	CORO6	1,104944	0,042422
ENSG00000108244	KRT23	1,252476	0,042553
ENSG00000214456	PLIN5	1,323625	0,042666
ENSG00000206172	HBA1	1,78898	0,043893
ENSG00000233429	HOTAIRM1	1,495009	0,04532
ENSG00000119922	IFIT2	1,213361	0,045902
ENSG00000140932	CMTM2	1,039572	0,045902
ENSG00000266236	Novel transcript	1,26194	0,046431
ENSG00000237772	LINC02631	1,069087	0,04686
ENSG00000268903	Novel transcript	1,017496	0,047245
ENSG00000163737	PF4	1,377697	0,047431
ENSG00000163430	FSTL1	1,792222	0,04821
ENSG00000106976	DNM1	1,170138	0,049339
ENSG00000206052	DOK6	1,21618	0,049431
ENSG00000180712	LINC02363	1,248682	0,049602
ENSG00000213937	CLDN9	1,167116	0,049616

ENSG00000182310	SPACA6	1,30568	0,049901
ENSG00000134765	DSC1	-1,86156	0,002693
ENSG00000091129	NRCAM	-1,87259	0,008739
ENSG00000180543	TSPYL5	-1,10085	0,011184
ENSG00000163492	CCDC141	-1,20217	0,011184
ENSG00000164930	FZD6	-1,27405	0,013464
ENSG00000275395	FCGBP	-1,11613	0,015764
ENSG00000134247	PTGFRN	-1,39082	0,015764
ENSG00000204792	LINC01291	-4,11036	0,016583
ENSG00000278318	ZNF229	-1,13563	0,016583
ENSG00000067646	ZFY	-5,53546	0,016583
ENSG00000114374	USP9Y	-5,92937	0,01722
ENSG00000115414	FN1	-1,96757	0,017462
ENSG00000134193	REG4	-1,79399	0,017666
ENSG00000227051	C14orf132	-1,47644	0,017697
ENSG00000289707	LOC105377225	-5,43881	0,019272
ENSG00000134531	EMP1	-1,44122	0,020876
ENSG00000129824	RPS4Y1	-6,15516	0,022427
ENSG00000184613	NELL2	-1,12394	0,022492
ENSG00000183878	UTY	-6,02852	0,022492
ENSG00000291033	TXLNGY	-5,44778	0,022929
ENSG00000099725	PRKY	-4,97748	0,023086
ENSG00000291031	BCORP1	-3,91296	0,024322
ENSG00000012817	KDM5D	-6,20264	0,024798
ENSG00000107249	GLIS3	-1,58876	0,025541
ENSG00000175445	LPL	-1,33991	0,025898
ENSG00000151320	AKAP6	-1,0653	0,0266
ENSG00000260197	Novel transcript	-4,02314	0,027069
ENSG00000097096	SYDE2	-1,01399	0,027701
ENSG00000130413	STK33	-1,06655	0,028377
ENSG00000067048	DDX3Y	-6,34823	0,029215
ENSG00000277117	LOC102723996	-2,74177	0,030722
ENSG00000198692	EIF1AY	-5,58933	0,032313
ENSG00000231535	LINC00278	-3,8569	0,032313
ENSG00000150760	DOCK1	-1,1451	0,032476
ENSG00000185915	KLHL34	-1,06714	0,036831
ENSG00000156395	SORCS3	-1,09706	0,03802
ENSG00000120645	IQSEC3	-2,13182	0,039292
ENSG00000165521	EML5	-1,09139	0,039748
ENSG00000176728	TTTY14	-3,18946	0,043423
ENSG00000227242	Novel transcript	-2,23465	0,048847
ENSG00000152672	CLEC4F	-2,05423	0,049837
ENSG00000101230	ISM1	-1,19597	0,049877

Table S.8. Top 367 genes showing significant variance across all groups (F-statistic > 30,000, FDR < 0.01 and AveExpr > 5)

	symbol	F	adj.P.Val
ENSG00000197622	CDC42SE1	60771,12	0
ENSG00000147140	NONO	71630,6	0
ENSG00000070756	PABPC1	57327,51	0
ENSG00000187239	FNBP1	59909,94	0
ENSG00000110321	EIF4G2	61049,11	0
ENSG00000048740	CELF2	60232,05	0
ENSG00000139644	TMBIM6	57961,03	0
ENSG00000089053	ANAPC5	57072,52	0
ENSG00000108669	CYTH1	66414,64	0
ENSG00000182944	EWSR1	73442,45	0
ENSG00000185811	IKZF1	56441,03	7,04537610969618e-321
ENSG00000078304	PPP2R5C	56178,87	1,29148759822902e-320
ENSG00000234745	HLA-B	55450,08	4,17287844477517e-320
ENSG00000265972	TXNIP	53760,02	1,01856079480986e-318
ENSG00000163466	ARPC2	53484,42	1,65331657396086e-318
ENSG00000009307	CSDE1	53362,49	1,98106984209902e-318
ENSG00000182934	SRPRA	53301,94	1,9934511271838e-318
ENSG00000165119	HNRNPK	53300,7	1,9934511271838e-318
ENSG00000111348	ARHGDIB	53145,63	2,58194259925823e-318
ENSG00000065883	CDK13	53119,01	2,58846920643979e-318
ENSG00000116478	HDAC1	52443,01	9,78334463991107e-318
ENSG00000129675	ARHGEF6	51363,79	8,74718670899329e-317
ENSG00000159658	EFCAB14	51081,73	1,51308641576738e-316
ENSG00000166710	B2M	50496,01	5,01471082171662e-316
ENSG00000157538	VPS26C	50377,31	6,20120196040656e-316
ENSG00000166501	PRKCB	50341,03	6,44327826736154e-316
ENSG00000136279	DBNL	49953,63	1,42452599360259e-315
ENSG00000160714	UBE2Q1	49690,34	2,42547111990214e-315
ENSG00000127483	HP1BP3	49206,92	6,70410562050294e-315
ENSG00000152291	TGOLN2	49085,87	8,4470107079497e-315
ENSG00000160058	BSDC1	49025,72	9,32720366078954e-315
ENSG00000005844	ITGAL	48733,09	1,72062712548577e-314
ENSG00000257103	LSM14A	48524,19	2,64871525459759e-314
ENSG00000107862	GBF1	48430,71	3,08218275788073e-314
ENSG00000198663	C6orf89	48429,38	3,08218275788073e-314
ENSG00000087274	ADD1	48109	6,119888576138e-314
ENSG00000075415	SLC25A3	47997,91	7,63579184246254e-314
ENSG00000092199	HNRNPC	47851,27	1,03331253631081e-313
ENSG00000100811	YY1	47477,76	2,33923894518668e-313
ENSG00000150867	PIP4K2A	47121,53	5,12809799169617e-313
ENSG00000153187	HNRNPU	47086,7	5,41721016186136e-313

ENSG00000187742	SECISBP2	47073,29	5,45286542212688e-313
ENSG00000160679	CHTOP	47042,62	5,71285740606049e-313
ENSG00000078369	GNB1	46489,74	1,99168112593557e-312
ENSG00000138668	HNRNPD	46388,16	2,46407949960301e-312
ENSG00000075624	ACTB	46129,89	4,39496222560023e-312
ENSG00000206503	HLA-A	46023,36	5,51609599085266e-312
ENSG00000147065	MSN	45952,53	6,37443965550654e-312
ENSG00000143119	CD53	45827,43	8,37249309896291e-312
ENSG00000173020	GRK2	45783	9,10742035627535e-312
ENSG00000100813	ACIN1	45774,42	9,11058095699766e-312
ENSG00000182149	IST1	45716,24	1,02456152392899e-311
ENSG00000171720	HDAC3	45705,74	1,03037558483726e-311
ENSG00000175224	ATG13	45564,65	1,41031243851913e-311
ENSG00000143393	PI4KB	45221,62	3,08578476658165e-311
ENSG00000119689	DLST	45218,92	3,08578476658165e-311
ENSG00000040933	INPP4A	45189,58	3,2509283940261e-311
ENSG00000108654	DDX5	44905,78	6,29182445460454e-311
ENSG00000074356	NCBP3	44809,71	7,78768084150754e-311
ENSG00000158604	TMED4	44667,94	1,07683474696078e-310
ENSG00000144021	CIAO1	44648,58	1,10974130950086e-310
ENSG00000159322	ADPGK	44615,18	1,18339310479158e-310
ENSG00000157916	RER1	44342,94	2,24968737194559e-310
ENSG00000115935	WIPF1	43862,76	7,14385960471619e-310
ENSG00000159202	UBE2Z	43834,11	7,54621795579033e-310
ENSG00000163636	PSMD6	43762,96	8,85083529823833e-310
ENSG00000149091	DGKZ	43552,94	1,46280529796141e-309
ENSG00000122566	HNRNPA2B1	43528,98	1,52920233719084e-309
ENSG00000170471	RALGAPB	43397,46	2,08683162996884e-309
ENSG00000149182	ARFGAP2	43190,51	3,4399280541549e-309
ENSG00000101109	STK4	43167,23	3,59396830307693e-309
ENSG00000123338	NCKAP1L	43105,76	4,13119558822837e-309
ENSG00000204256	BRD2	42835,69	8,01119303068273e-309
ENSG00000116698	SMG7	42774,65	9,21298309363105e-309
ENSG00000048828	FAM120A	42737,9	9,97061479130496e-309
ENSG00000170445	HARS1	42363,37	2,53609630984227e-308
ENSG00000095787	WAC	42306,47	2,89250661000391e-308
ENSG00000144567	RETREG2	42248,19	3,31185172259926e-308
ENSG00000183283	DAZAP2	42228,88	3,43471889837194e-308
ENSG00000266094	RASSF5	42035,28	5,56007179420934e-308
ENSG00000141644	MBD1	41983,64	6,26781518170278e-308
ENSG00000058262	SEC61A1	41855,37	8,60453034019334e-308
ENSG00000197111	PCBP2	41657,3	1,41595626816258e-307
ENSG00000158195	WASF2	41616,64	1,55404603385781e-307
ENSG00000170248	PDCD6IP	41506,58	2,04190900753719e-307

ENSG00000168066	SF1	41404,79	2,62817063548119e-307
ENSG00000116584	ARHGEF2	41388,43	2,71081030093482e-307
ENSG00000010244	ZNF207	41347,2	2,9832408974622e-307
ENSG00000186517	ARHGAP30	41332,98	3,06088154610005e-307
ENSG00000166913	YWHAB	41192,36	4,36706596087166e-307
ENSG00000113141	IK	41166,46	4,62136211386867e-307
ENSG00000111252	SH2B3	41143,01	4,86006869225456e-307
ENSG00000136167	LCP1	41068,29	5,84606614547891e-307
ENSG00000089280	FUS	40958,21	7,65537034678736e-307
ENSG00000028528	SNX1	40957,35	7,65537034678736e-307
ENSG00000198728	LDB1	40953,38	7,65537034678736e-307
ENSG00000184007	PTP4A2	40836,3	1,03085693892368e-306
ENSG00000075413	MARK3	40812,7	1,08580575094198e-306
ENSG00000167323	STIM1	40701,95	1,43972093620984e-306
ENSG00000197217	ENTPD4	40690,37	1,46959309787898e-306
ENSG00000130254	SAFB2	40593,24	1,88157357572561e-306
ENSG00000165678	GHITM	40574,32	1,9589206622066e-306
ENSG00000122122	SASH3	40483,22	2,47049077289189e-306
ENSG00000168918	INPP5D	40466,02	2,56113385120363e-306
ENSG00000133872	SARAF	40435,44	2,75165588295728e-306
ENSG00000166887	VPS39	40369,51	3,24867690062837e-306
ENSG00000187555	USP7	40307,16	3,80032398735346e-306
ENSG00000168385	SEPTIN2	40241,42	4,48777176254645e-306
ENSG00000136490	LIMD2	40135,57	5,90279692095545e-306
ENSG00000197448	GSTK1	40044,1	7,47606108751048e-306
ENSG00000148730	EIF4EBP2	40023,1	7,83868438565231e-306
ENSG00000106948	AKNA	39932,96	9,90081468095738e-306
ENSG00000159692	CTBP1	39903,26	1,0630839278223e-305
ENSG00000138029	HADHB	39854,8	1,20088312508436e-305
ENSG00000108604	SMARCD2	39835,95	1,25258479495015e-305
ENSG00000131051	RBM39	39829,95	1,26207358811721e-305
ENSG00000110075	PPP6R3	39759,45	1,51395905431426e-305
ENSG00000153827	TRIP12	39695,62	1,78427646010377e-305
ENSG00000172757	CFL1	39667,75	1,9081155812365e-305
ENSG00000135829	DHX9	39619,37	2,15765321193013e-305
ENSG00000092847	AGO1	39472,87	3,18726713995761e-305
ENSG00000196704	AMZ2	39275,84	5,41475751572327e-305
ENSG00000110955	ATP5F1B	39272,36	5,42218882568373e-305
ENSG00000052841	TTC17	39233,15	5,98843808364395e-305
ENSG00000159592	GPBP1L1	39172,49	7,0163743723761e-305
ENSG00000121210	TMEM131L	39163,24	7,13957844319725e-305
ENSG00000131504	DIAPH1	39138,64	7,57877266528924e-305
ENSG00000100201	DDX17	39126,37	7,77134774332521e-305
ENSG00000143569	UBAP2L	39123,82	7,77134774332521e-305
ENSG00000163513	TGFBR2	39093,15	8,39036806884913e-305

ENSG00000076928	ARHGEF1	38970,63	1,16700700003643e-304
ENSG00000103479	RBL2	38928,09	1,30253649016774e-304
ENSG00000225733	FGD5-AS1	38838,69	1,65537915929909e-304
ENSG00000169499	PLEKHA2	38803,72	1,81018167673287e-304
ENSG00000101191	DIDO1	38797,09	1,8301005028969e-304
ENSG00000108219	TSPAN14	38686	2,47293499965504e-304
ENSG00000102879	CORO1A	38663,34	2,61455648962319e-304
ENSG00000139990	DCAF5	38593,84	3,14970837948316e-304
ENSG00000182446	NPLOC4	38572,01	3,32328741743009e-304
ENSG00000198668	CALM1	38566,1	3,35440693109708e-304
ENSG00000081237	PTPRC	38513,5	3,85716665069841e-304
ENSG00000143549	TPM3	38367,52	5,76216472817825e-304
ENSG00000205531	NAP1L4	38362,06	5,81012756911303e-304
ENSG00000072786	STK10	38326,06	6,38272897191192e-304
ENSG00000060237	WNK1	38307,77	6,67272979471644e-304
ENSG00000118816	CCNI	38274,78	7,27043521655353e-304
ENSG00000184787	UBE2G2	38242,97	7,89634698382575e-304
ENSG00000067560	RHOA	38234,12	8,04066259924532e-304
ENSG00000125875	TBC1D20	38209,93	8,5493691395271e-304
ENSG00000069424	KCNAB2	38024,12	1,43453782364774e-303
ENSG00000130227	XPO7	37879,17	2,14894489034568e-303
ENSG00000164924	YWHAZ	37762,63	2,97357976479347e-303
ENSG00000141002	TCF25	37668,12	3,86787870742038e-303
ENSG00000156599	ZDHHC5	37532,06	5,6710225206583e-303
ENSG00000172943	PHF8	37426,12	7,63629905300862e-303
ENSG00000007168	PAFAH1B1	37395,95	8,22208816027536e-303
ENSG00000125107	CNOT1	37395,95	8,22208816027536e-303
ENSG00000105698	USF2	37370,42	8,79268223489199e-303
ENSG00000110934	BIN2	37336,67	9,62895519516211e-303
ENSG00000130414	NDUFA10	37294,14	1,08166916883119e-302
ENSG00000005483	KMT2E	37291,09	1,0844400402117e-302
ENSG00000184009	ACTG1	37219,8	1,32398849367384e-302
ENSG00000198231	DDX42	37033,01	2,26049291199404e-302
ENSG00000213676	ATF6B	36980,4	2,6179267811059e-302
ENSG00000026508	CD44	36947,75	2,86130424284851e-302
ENSG00000166986	MARS1	36940,33	2,90616642996521e-302
ENSG00000071127	WDR1	36775,85	4,66813988857152e-302
ENSG00000103653	CSK	36732,63	5,26584688746235e-302
ENSG00000068308	OTUD5	36679,47	6,11695899496952e-302
ENSG00000136758	YME1L1	36664,23	6,35890449687056e-302
ENSG00000140632	GLYR1	36451,78	1,18100857221717e-301
ENSG00000105323	HNRNPUL1	36379,59	1,45316275434804e-301
ENSG00000026025	VIM	36368,51	1,49288405064179e-301
ENSG00000100242	SUN2	36320,62	1,71024846444495e-301

ENSG00000136709	WDR33	36318,36	1,71190530440252e-301
ENSG00000116350	SRSF4	36038,08	3,91595779194491e-301
ENSG00000151502	VPS26B	35957,52	4,95340360392453e-301
ENSG00000122218	COPA	35875,91	6,28883076211919e-301
ENSG00000144579	CTDSP1	35827,33	7,23473957159274e-301
ENSG00000204592	HLA-E	35823,27	7,28287137204052e-301
ENSG00000121774	KHDRBS1	35813,6	7,4560743601225e-301
ENSG00000172775	PSME3IP1	35799,1	7,7450273124411e-301
ENSG00000078808	SDF4	35793,49	7,83365117176503e-301
ENSG00000136754	ABI1	35788,41	7,91079707529548e-301
ENSG00000165527	ARF6	35736,26	9,20415160421348e-301
ENSG00000183020	AP2A2	35697,05	1,03016601983407e-300
ENSG00000075568	TMEM131	35585,6	1,43424817836576e-300
ENSG00000175387	SMAD2	35543,46	1,62046455187702e-300
ENSG00000103275	UBE2I	35514,35	1,76038761906963e-300
ENSG00000091527	CDV3	35496,14	1,85041539337492e-300
ENSG00000148248	SURF4	35425,16	2,28283450737091e-300
ENSG00000185591	SP1	35395,87	2,48220622191879e-300
ENSG00000181090	EHMT1	35285,5	3,45493101024651e-300
ENSG00000172046	USP19	35273,35	3,56681737806056e-300
ENSG00000132670	PTPRA	35261,01	3,68457048463703e-300
ENSG00000065413	ANKRD44	35255,95	3,72280727006284e-300
ENSG00000054118	THRAP3	35183,65	4,61881401011014e-300
ENSG00000113648	MACROH2A1	35120,65	5,5721662939171e-300
ENSG00000104133	SPG11	35078,3	6,3122899492792e-300
ENSG00000163131	CTSS	34982,59	8,42576387933585e-300
ENSG00000167522	ANKRD11	34980,02	8,45043183374117e-300
ENSG00000116731	PRDM2	34974,16	8,56152361613793e-300
ENSG00000178950	GAK	34968,92	8,6576883962517e-300
ENSG00000128340	RAC2	34942,3	9,35044083484372e-300
ENSG00000148358	GPR107	34929,66	9,62871453517236e-300
ENSG00000177479	ARIH2	34929,6	9,62871453517236e-300
ENSG00000151923	TIAL1	34898,15	1,05568822420964e-299
ENSG00000196821	ILRUN	34890,82	1,07461103103711e-299
ENSG00000136451	VEZF1	34884,86	1,08932468594152e-299
ENSG00000168488	ATXN2L	34846,67	1,21963554795191e-299
ENSG00000147526	TACC1	34765,22	1,56121426989184e-299
ENSG00000148700	ADD3	34719,81	1,78833788156864e-299
ENSG00000205250	E2F4	34706,43	1,85523849574963e-299
ENSG00000162434	JAK1	34697,87	1,89623752024471e-299
ENSG00000107929	LARP4B	34634,87	2,29479883052258e-299
ENSG00000198815	FOXJ3	34632,39	2,3017920646239e-299
ENSG00000144028	SNRNP200	34602,95	2,51057398348843e-299
ENSG00000105221	AKT2	34584,26	2,64858493074157e-299
ENSG00000115524	SF3B1	34579,87	2,67271720803065e-299

ENSG00000083799	CYLD	34553,49	2,88809923941698e-299
ENSG00000102910	LONP2	34545,67	2,94591879664003e-299
ENSG00000161203	AP2M1	34537,23	3,01071305776224e-299
ENSG00000134748	PRPF38A	34515,73	3,20481684227713e-299
ENSG00000066117	SMARCD1	34498,35	3,36802180449158e-299
ENSG00000177885	GRB2	34480,34	3,54672631983362e-299
ENSG00000090060	PAPOLA	34323,53	5,76475357989016e-299
ENSG00000103187	COTL1	34317,77	5,8438805783619e-299
ENSG00000167258	CDK12	34207,65	8,22019158486437e-299
ENSG00000079246	XRCC5	34200,06	8,38189095296074e-299
ENSG00000163902	RPN1	34144,53	9,93906341496609e-299
ENSG00000007202	BLTP2	33957,11	1,78854039153626e-298
ENSG00000171681	ATF7IP	33869,4	2,35184748943349e-298
ENSG00000138107	ACTR1A	33847,21	2,5127520884509e-298
ENSG00000110324	IL10RA	33822,12	2,70967274683338e-298
ENSG00000092330	TINF2	33819,19	2,7234440525311e-298
ENSG00000164091	WDR82	33817,71	2,72466744094252e-298
ENSG00000134453	RBM17	33660,24	4,48165625774967e-298
ENSG00000072818	ACAP1	33647,39	4,64987688668444e-298
ENSG00000131236	CAP1	33617,19	5,09987983333872e-298
ENSG00000129351	ILF3	33512,21	7,10887506387543e-298
ENSG00000081791	DELE1	33485,41	7,71536606197546e-298
ENSG00000109332	UBE2D3	33361,48	1,1447617307722e-297
ENSG00000275066	SYNRG	33307,34	1,35758247201576e-297
ENSG00000019582	CD74	33305,57	1,35979490267927e-297
ENSG00000203879	GDI1	33295,93	1,39701803584831e-297
ENSG00000204463	BAG6	33280,97	1,46022861428506e-297
ENSG00000101266	CSNK2A1	33275,79	1,47776716357605e-297
ENSG00000105401	CDC37	33274,77	1,47776716357605e-297
ENSG00000160310	PRMT2	33244,02	1,62568588682198e-297
ENSG00000149480	MTA2	33239,39	1,64362957773629e-297
ENSG00000071626	DAZAP1	33212,53	1,78573620360288e-297
ENSG00000135930	EIF4E2	33211,11	1,78687709425496e-297
ENSG00000143384	MCL1	33160,3	2,09827681979103e-297
ENSG00000117713	ARID1A	33134,68	2,27117331507238e-297
ENSG00000107099	DOCK8	33127,69	2,31414745013291e-297
ENSG00000169926	KLF13	33119,01	2,37102563741183e-297
ENSG00000157954	WIPI2	33116,48	2,38123435906723e-297
ENSG00000185627	PSMD13	33113,04	2,39868689391046e-297
ENSG00000198624	CCDC69	32995,1	3,5068601501014e-297
ENSG00000143442	POGZ	32881,95	5,05426548881447e-297
ENSG00000157191	NECAP2	32873,85	5,17002508054394e-297
ENSG00000166295	ANAPC16	32861,92	5,35525625139484e-297
ENSG00000184640	SEPTIN9	32823,54	6,04925560954443e-297

ENSG00000101193	GID8	32812,98	6,23852843628074e-297
ENSG00000105063	PPP6R1	32810,5	6,26559722373957e-297
ENSG00000140943	MBTPS1	32774,6	7,02193695431258e-297
ENSG00000188938	FAM120AOS	32729,11	8,12231427752673e-297
ENSG00000166888	STAT6	32723,96	8,23026631835638e-297
ENSG00000006125	AP2B1	32691,05	9,13632206925793e-297
ENSG00000055163	CYFIP2	32671,14	9,71898154052682e-297
ENSG00000008513	ST3GAL1	32658,77	1,00853527278187e-296
ENSG00000105568	PPP2R1A	32651,89	1,02787223468664e-296
ENSG00000138279	ANXA7	32625,2	1,11821846222994e-296
ENSG00000167658	EEF2	32570,64	1,33382595697104e-296
ENSG00000173120	KDM2A	32564,85	1,35461006401965e-296
ENSG00000100991	TRPC4AP	32504,65	1,64680570095725e-296
ENSG00000011304	PTBP1	32476,08	1,80360998261717e-296
ENSG00000135926	TMBIM1	32469,5	1,83669725112524e-296
ENSG00000161904	LEMD2	32464,98	1,85770588857784e-296
ENSG00000090863	GLG1	32427,98	2,09251831821059e-296
ENSG00000204469	PRRC2A	32384,23	2,41082730883273e-296
ENSG00000166783	MARF1	32373,4	2,49022133681137e-296
ENSG00000198001	IRAK4	32357,56	2,61555229081141e-296
ENSG00000104880	ARHGEF18	32242,35	3,82465844435837e-296
ENSG00000112079	STK38	32144,24	5,2889505122418e-296
ENSG00000119844	AFTPH	32016,82	8,07837742761377e-296
ENSG00000197746	PSAP	32009,06	8,2626525590851e-296
ENSG00000126216	TUBGCP3	32006,4	8,30792881094722e-296
ENSG00000138071	ACTR2	31997,69	8,52508800212757e-296
ENSG00000122557	HERPUD2	31945,27	1,01333666099857e-295
ENSG00000131446	MGAT1	31905,79	1,15031280565565e-295
ENSG00000145495	MARCHF6	31905,59	1,15031280565565e-295
ENSG00000079459	FDFT1	31902,72	1,15750110909016e-295
ENSG00000080503	SMARCA2	31857,01	1,34583775912196e-295
ENSG00000149187	CELF1	31851,82	1,36500548292934e-295
ENSG00000151702	FLI1	31804,78	1,59467685399199e-295
ENSG00000166974	MAPRE2	31751,96	1,90029447677307e-295
ENSG00000100796	PPP4R3A	31710,05	2,18291313923064e-295
ENSG00000133226	SRRM1	31690,1	2,32790206523149e-295
ENSG00000198399	ITSN2	31615,77	2,98660755627625e-295
ENSG00000074370	ATP2A3	31512,23	4,23553488328411e-295
ENSG00000004455	AK2	31503,82	4,34438968319472e-295
ENSG00000104365	IKBKB	31491,1	4,52213708616805e-295
ENSG00000115307	AUP1	31473,97	4,77879480986685e-295
ENSG00000143761	ARF1	31472,68	4,78422419386285e-295
ENSG00000077549	CAPZB	31464,02	4,91169461477117e-295
ENSG00000204525	HLA-C	31460,51	4,95482983705962e-295
ENSG00000119638	NEK9	31449,44	5,12916580534262e-295

ENSG00000115091	ACTR3	31411,3	5,82511081635022e-295
ENSG00000104894	CD37	31407,26	5,88717407011171e-295
ENSG00000275023	MLLT6	31395,08	6,11813971555533e-295
ENSG00000174243	DDX23	31373,22	6,57292205154104e-295
ENSG00000205542	TMSB4X	31349,58	7,10487697800283e-295
ENSG00000174231	PRPF8	31335,26	7,43889181475418e-295
ENSG00000125944	HNRNPR	31270,16	9,25599609964544e-295
ENSG00000180448	ARHGAP45	31269,78	9,25599609964544e-295
ENSG00000175203	DCTN2	31266,32	9,33739741225745e-295
ENSG00000198771	RCSD1	31253,6	9,72413299935137e-295
ENSG00000132466	ANKRD17	31231,22	1,04696889802116e-294
ENSG00000147010	SH3KBP1	31230,03	1,04799942757693e-294
ENSG00000061936	SFSWAP	31223,14	1,06981992528594e-294
ENSG00000106609	TMEM248	31214,94	1,09704521436765e-294
ENSG00000155463	OXA1L	31177,5	1,24427494909427e-294
ENSG00000054611	TBC1D22A	31172,13	1,26360438803945e-294
ENSG00000120733	KDM3B	31155,3	1,33503162940815e-294
ENSG00000137776	SLTM	31135,13	1,42691255571573e-294
ENSG00000072609	CHFR	31127,69	1,45959822727606e-294
ENSG00000167978	SRRM2	31018,52	2,12296772359098e-294
ENSG00000185896	LAMP1	30979,94	2,4195436741073e-294
ENSG00000166716	ZNF592	30966,47	2,52768019106748e-294
ENSG00000114353	GNAI2	30960,75	2,57064709039112e-294
ENSG00000125354	SEPTIN6	30907,42	3,0848592134428e-294
ENSG00000163320	CGGBP1	30889,64	3,2718355747489e-294
ENSG00000099995	SF3A1	30871,01	3,48058527374846e-294
ENSG00000197343	ZNF655	30854,39	3,67708893693694e-294
ENSG00000168137	SETD5	30852	3,6968660913878e-294
ENSG00000090686	USP48	30829,36	3,98849502901531e-294
ENSG00000023892	DEF6	30822,1	4,07877169924001e-294
ENSG00000158985	CDC42SE2	30795,38	4,46408639724629e-294
ENSG00000124226	RNF114	30748,21	5,24848923427411e-294
ENSG00000172349	IL16	30722,75	5,72044355695122e-294
ENSG00000121022	COPS5	30687,19	6,46004804893752e-294
ENSG00000099917	MED15	30615,71	8,27672734982707e-294
ENSG00000115216	NRBP1	30609,49	8,43501514946574e-294
ENSG00000108312	UBTF	30596,47	8,80409977328534e-294
ENSG00000010810	FYN	30464,58	1,39682022369126e-293
ENSG00000164574	GALNT10	30430,57	1,57049127539975e-293
ENSG00000088930	XRN2	30424,67	1,59894935295524e-293
ENSG00000125755	SYMPK	30415,58	1,6464571647599e-293
ENSG00000125970	RALY	30393,34	1,77606185637304e-293
ENSG00000213445	SIPA1	30389,34	1,79625782980795e-293
ENSG00000105329	TGFB1	30345,72	2,09021295703799e-293

ENSG00000186298	PPP1CC	30293,47	2,50861832536031e-293
ENSG00000166340	TPP1	30289,03	2,54122931243069e-293
ENSG00000129933	MAU2	30268	2,73054871721952e-293
ENSG00000100422	CERK	30245	2,95476993988027e-293
ENSG00000139613	SMARCC2	30226,97	3,14157863826506e-293
ENSG00000115762	PLEKHB2	30222,14	3,18703781415997e-293
ENSG00000087460	GNAS	30214,48	3,26593961794161e-293
ENSG00000127528	KLF2	30161,51	3,93303330529063e-293
ENSG00000165280	VCP	30142,13	4,20273458004551e-293
ENSG00000141524	TMC6	30117,71	4,57274592872894e-293
ENSG00000066136	NFYC	30095,03	4,94486241321621e-293
ENSG00000149930	TAOK2	30077,39	5,25208452058641e-293
ENSG00000102974	CTCF	30070,68	5,36481569551327e-293
ENSG00000151883	PARP8	30063,77	5,48406314739511e-293
ENSG00000125447	GGA3	30014,43	6,52532633333336e-293

Table S.9. Top Reactome Pathways enriched of globally significant genes across experimental groups

Term	Adjusted P-value	Genes
FLT3 Signaling	0.00649499314046781	AKT2;CSK;FYN;GRB2;SH2B3
RAC1 GTPase Cycle	0.00649499314046781	CYFIP2;ARHGAP30;WIPF1;DOCK8; ARHGDIB;ABI1 ARHGEF18;NCKAP1L;WASF2;DEF6; ARHGEF6
ER to Golgi Anterograde Transport	0.00642099211684526	COPA;ARF1;ACTR1A;TBC1D20;CAPZB;DCTN2; PPP6R1;PPP6R3;GBF1;ARFGAP2
Asparagine N-linked Glycosylation	0.00621496342573964	ARF1;COPA;VCP;DCTN2;RPN1;GBF1;ARFGAP2; ACTR1A;TBC1D20;CAPZB;PPP6R1;PPP6R3;MGAT1; ST3GAL1;MARCHF6
Interleukin-12 Family Signaling	0.0059697386842594	ARF1;CFL1;HNRNPA2B1;MSN;LCP1;JAK1
Gene and Protein Expression by JAK-STAT Signaling After Interleukin-12 Stimulation	0.00596103570045946	ARF1;CFL1;HNRNPA2B1;MSN;LCP1
Developmental Biology	0.00595031305281464	SMARCD1;SMARCD2;HDAC3;LDB1;SH3KBP1;PSMD13; HDAC1;HNRNPU;CTCF;AP2A2;MED15;ACTB;ACTG1; YY1;PSMD6;AKT2;CFL1;FYN;MTA2;AP2M1;CAP1;SMAD2; ACTR3;ACTR2;SMARCC2;TGFB1;CSNK2A1;MARS1;MSN; AP2B1;ARID1A;SMARCA2;FLI1;RHOA;DIAPH1;PTPRC;ARPC2; CNOT1;PTPRA;THRAP3;AGO1;GRB2;PABPC1
Host Interactions of HIV Factors	0.00595031305281464	PSMD6;ARF1;PSMD13;FYN;HLA-A;AP2B1;B2M;AP2A2;AP2M1
MHC Class II Antigen Presentation	0.00595031305281464	CD74;ARF1;ACTR1A;CAPZB;DCTN2;AP2B1;AP2A2;AP2M1;CTSS
CTLA4 Inhibitory Signaling	0.00590467450577589	PPP2R1A;AKT2;FYN;PPP2R5C
Signaling by High-Kinase Activity BRAF Mutants	0.00547593545773775	YWHAB;CSK;MARK3;ACTB;ACTG1
Axon Guidance	0.00540991625480496	CAP1;ACTR3;ACTR2;CSNK2A1;LDB1;SH3KBP1;PSMD13; MSN;AP2B1;AP2A2;ACTB;RHOA;ACTG1;PSMD6;PTPRC; ARPC2;PTPRA;CFL1;GRB2;FYN;PABPC1;AP2M1
Deubiquitination	0.00540991625480496	SMAD2;OTUD5;USP7;VCP;USP48;TGFB1;PSMD13;ACTB; RHOA;USP19;TGFBR2;YY1;CYLD;PSMD6
P75 NTR Receptor-Mediated Signalling	0.00528387835590029	IKBKB;HDAC3;HDAC1;ARHGEF18;ARHGEF1;ARHGEF2;RHOA;ARHGEF6

Transcriptional Regulation by TP53	0.00495035815588377	USP7;CSNK2A1;YWHAB;HDAC1;EHMT1;PPP2R5C; YWHAZ;RBL2;PPP2R1A;CNOT1;AKT2;AGO1;PIP4K2A; CDK12;E2F4;CDK13;MTA2
Transcriptional Regulation by RUNX1	0.00451953605395934	SMARCD1;PSMD6;SMARCD2;SMARCC2;CSNK2A1; LDB1;PRKCB;PSMD13;HDAC1;AGO1;ARID1A;SMARCA2
SARS-CoV-1 Targets Host Intracellular Signalling and Regulatory Pathways	0.0042069328405486	UBE2I;YWHAB;SP1;YWHAZ
Epigenetic Regulation of Gene Expression	0.00418705703540095	ATF7IP;SMARCD1;SMARCD2;SMARCC2;UBE2I;HDAC3; HDAC1;EHMT1;ARID1A;SMARCA2;ACTB;WDR82;UBTF;MTA2;SF3B1
EPH-ephrin Mediated Repulsion of Cells	0.00407721045445314	FYN;AP2B1;AP2A2;ACTB;AP2M1;ACTG1
Nef Mediated CD8 Down-regulation	0.00402745664749563	AP2B1;AP2A2;AP2M1

Table S.10. Reactome Pathway enrichment analysis of the 269-gene signature differentiating from active TB vs. Controls

Term	Adj.p value	Genes
Neutrophil Degranulation R-HSA-6798695	1,06E+03	SERPINB10;CRISP3;GPR84;RETN;ABCA13; MPO;PLAU;S100A12;OLR1;CTSG;PGLYRP1; ELANE;CD177;DEFA4;MCEMP1;R-SE3;AZU1 ;OLFM4;MMP8;R-SE1;MMP9;CHIT1; CEACAM1;VNN1;TCN1;CEACAM6;TARM1; LCN2;PRTN3;BPI;CEACAM8;S100A9;S100A8; LTF
Innate Immune System R-HSA-168249	3,49E+06	C1QB;SERPINB10;C1QA;CRISP3;GPR84;RETN ;ABCA13;MPO;SOCS1;PLAU;S100A12;OLR1; CTSG;CCR6;PGLYRP1;FCGR1A;ELANE;CD177; KCNH8;DEFA4;MCEMP1;R-SE3;AZU1; OLFM4;MMP8;S100B;R-SE1;MMP9;CHIT1; CEACAM1;VNN1;AIM2;TCN1;CEACAM6; TARM1;LCN2;SERPING1;PRTN3;BPI;CEACAM8 ;S100A9;S100A8;C1QC;LTF
Immune System R-HSA-168256	3,49E+06	IFITM3;CRISP3;ABCA13;MPO;IFIT3; CA1;PLAU;GRB10;OLR1;FBXO6; CTSG;CCR6;PGLYRP1;CD177; GBP6;GBP5;RSAD2;KCNH8;MCEMP1; R-SE3;PDCD1LG2;OLFM4;MMP8 ;R-SE1;MMP9;CHIT1;CEACAM1;AIM2;IFI27;CEACAM6; SERPING1;PRTN3;BPI;CEACAM8;S100A9;S100A8;LTF; C1QB;SERPINB10;C1QA;CD274;GPR84;RETN;SOCS3; SOCS1;S100A12;KLRC1;GBP2;FCGR1A;GBP1;ELANE; DEFA4;AZU1;PTPN13;S100B;VNN1;TCN1;TARM1; LCN2;SDC1;FGF13;NECTIN2;C1QC
Extracellular Matrix Organization R-HSA-1474244	1,49E+08	COL17A1;PCOLCE2;SDC3;I TGA2B;HTRA1;MMP8;FBLN2; MMP9;COL19A1;CAPN13; CEACAM1;ADAMTS2;CEACAM6; COL4A3;SDC1;ITGA7;CTSG; CEACAM8;FGF13;ELANE
Antimicrobial Peptides R-HSA-6803157	1,53E+09	DEFA4;LCN2;CTSG;BPI;PRTN3; CCR6;R-SE3;PGLYRP1;S100A9; S100A8;ELANE;LTF
Metal Sequestration By Antimicrobial Proteins R-HSA-6799990	3,51E+10	LCN2;S100A9;S100A8;LTF
Cytokine Sig-ling In Immune System R-HSA-1280215	9,15E+11	IFITM3;GBP6;GBP5;RSAD2; KCNH8;PTPN13;S100B;MMP9; IFIT3;SOCS3;CA1;SOCS1;IFI27;GRB10;LCN2;S100A12; ;CTSG;PRTN3;FGF13;GBP2;FCGR1A;S100A9;GBP1
Interferon Alpha/Beta Sig-ling R-HSA-909733	0,0022	IFITM3;SOCS3;SOCS1;RSAD2;IFI27;GBP2;IFIT3

Classical Antibody-Mediated Complement Activation R-HSA-173623	0,0022	C1QB;C1QA;C1QC
Fibronectin Matrix Formation R-HSA-1566977	0,0022	CEACAM1;CEACAM6;CEACAM8
Interferon Sig-ling R-HSA-913531	0,0026	IFITM3;GBP6;GBP5;SOCS3;SOCS1;RSAD2;IFI27;FCGR1A ;GBP2;GBP1;IFIT3
Degradation Of Extracellular Matrix R-HSA-1474228	0,0035	COL17A1;CAPN13;HTRA1;CTSG; MMP8;MMP9;ELANE;COL19A1
Interferon Gamma Sig-ling R-HSA-877300	0,0058	GBP6;GBP5;SOCS3;SOCS1;FCGR1A;GBP2;GBP1
Collagen Degradation R-HSA-1442490	0,0058	COL17A1;MMP8;MMP9;COL19A1;ELANE
Regulation Of Complement Cascade R-HSA-977606	0,0096	C1QB;C1QA;SERPING1;ELANE; C1QC
Cell Surface Interactions At Vascular Wall R-HSA-202733	0,0112	CEACAM1;CEACAM6;SDC3;SDC1; OLR1;CEACAM8;S100A9;CD177
Complement Cascade R-HSA-166658	0,0208	C1QB;C1QA;SERPING1;ELANE; C1QC
Creation Of C4 And C2 Activators R-HSA-166786	0,0208	C1QB;C1QA;C1QC
Activation Of Matrix Metalloprotei-ses R-HSA-1592389	0,0230	CTSG;MMP8;MMP9;ELANE
Collagen Formation R-HSA-1474290	0,0285	COL17A1;ADAMTS2;PCOLCE2;COL4A3;MMP9; COL19A1
Hemostasis R-HSA-109582	0,0312	SDC3;ITGA2B;R-SE3;HBD;LHFPL2;R-SE1;CEACAM1;CEACAM6;PLAU;KCNMA1;SERPING1;SDC1; OLR1;PRTN3;CEACAM8;S100A9;CD177
Collagen Biosynthesis And Modifying Enzymes R-HSA-1650814	0,0414	COL17A1;ADAMTS2;PCOLCE2; COL4A3;COL19A1
Sig-ling By SCF-KIT R-HSA-1433557	0,0456	SOCS1;GRB10;MMP9;R-SE1

Syndecan Interactions R-HSA-3000170	0,0456	SDC3;SDC1;FGF13
Sig-ling By Interleukins R-HSA-449147	0,0456	KCNH8;PTPN13;S100B;MMP9; SOCS3;CA1;SOCS1;LCN2;S100A12 ;SDC1;CTSG;PRTN3;FGF13; S100A9
Toll Like Receptor 4 (TLR4) Cascade R-HSA-166016	0,0456	SOCS1;KCNH8;S100A12;BPI; S100B;S100A9;S100A8
Initial Triggering Of Complement R-HSA-166663	0,0474	C1QB;C1QA;C1QC

Table S.11. Reactome Pathway enrichment analysis of the 294-gene signature differentiating from active TB vs. IGRA/TST+ contacts

Term	adj, p value	Genes
Neutrophil Degranulation R-HSA-6798695	1,15E+03	SERPINB10;TNFAIP6;CRISP3;GPR84;RETN;ABCA13; MPO;PLAU;S100A12;OLR1;CTSG;PGLYRP1;ELANE; CD177;AOC1;DEFA4;MCEMP1;AZU1;OLFM4; MMP8;RNASE1;MMP9;CEACAM1;VNN1;TCN1; CEACAM6;TARM1;LCN2;PRTN3;BPI;CEACAM8; S100A9;S100A8;SIGLEC5;LTF
Innate Immune System R-HSA-168249	3,74E+06	C1QB;SERPINB10;C1QA;TNFAIP6;CRISP3;C4BPA; GPR84;RETN;ABCA13;MPO;PLAU;S100A12;OLR1; CTSG;CCR6;PGLYRP1;FCGR1A;ELANE;CD177; AOC1;KCNH8;DEFA4;MCEMP1;AZU1;OLFM4; MMP8;RNASE1;MMP9;CEACAM1;VNN1;AIM2;TCN1; CEACAM6;TARM1;LCN2;SERPING1;PRTN3;BPI; CEACAM8;S100A9;S100A8;C1QC;SIGLEC5;LTF
Immune System R-HSA-168256	1,97E+08	IFITM3;TNFAIP6;CRISP3;C4BPA;IL27;TNFRSF13C; ABCA13;MPO;IFIT3;CA1;PLAU;GRB10;OLR1;CTSG; CCR6;PGLYRP1;CD177;GBP6;GBP5;RSAD2;KCNH8; MCEMP1;PDCD1LG2;OLFM4;MMP8;RNASE1; MMP9;CEACAM1;AIM2;IFI27; CEACAM6;SERPING1;PRTN3;BPI; CEACAM8;S100A9;S100A8;LTF; C1QB;SERPINB10;C1QA;CD274; GPR84;RETN;S100A12;FCGR1A; GBP1;ELANE;BTNL9;AOC1; SIGLEC11;DEFA4;AZU1;VNN1; TCN1;TARM1;LCN2;SDC1;FGF13; NECTIN2;C1QC;SIGLEC5
Antimicrobial Peptides R-HSA-6803157	4,55E+09	DEFA4;LCN2;CTSG;BPI;PRTN3; CCR6;PGLYRP1;S100A9;S100A8; ELANE;LTF

Extracellular Matrix Organization R-HSA-1474244	1,11E+11	COL17A1;PCOLCE2;ITGA2B;NTN4; MMP8;MMP9;COL19A1;CEACAM1; ADAMTS2;CEACAM6;COL4A3;SDC1; MMP19;ITGA7;CTSG;CEACAM8;FGF13 ;ELANE
Metal Sequestration By Antimicrobial Proteins R-HSA-6799990	4,43E+10	LCN2;S100A9;S100A8;LTF
Collagen Degradation R-HSA-1442490	0,0011973168579897482	COL17A1;MMP19;MMP8;MMP9; COL19A1;ELANE
Regulation Of Complement Cascade R-HSA-977606	0,00210058879420826	C1QB;C1QA;SERPING1;C4BPA; ELANE;C1QC
Classical Antibody-Mediated Complement Activation R-HSA-173623	0,0026211283288578467	C1QB;C1QA;C1QC
Fibronectin Matrix Formation R-HSA-1566977	0,0026211283288578467	CEACAM1;CEACAM6;CEACAM8
Erythrocytes Take Up Oxygen And Release Carbon Dioxide R-HSA-1247673	0,004128163054702755	CA1;CA4;SLC4A1
Complement Cascade R-HSA-166658	0,004450763032643869	C1QB;C1QA;SERPING1;C4BPA; ELANE;C1QC
Erythrocytes Take Up Carbon Dioxide And Release Oxygen R-HSA-1237044	0,015817574817015567	CA1;CA4;SLC4A1
Cell Surface Interactions At Vascular Wall R-HSA-202733	0,01923786628620083	VPREB3;CEACAM1;CEACAM6; SDC1;OLR1;CEACAM8;S100A9; CD177
Cytokine Signaling In Immune System R-HSA-1280215	0,02266388341661716	IFITM3;GBP6;GBP5;RSAD2;KCNH8; IL27;TNFRSF13C;MMP9;IFIT3;CA1; IFI27;GRB10;LCN2;S100A12;SDC1; CTSG;PRTN3;FGF13;FCGR1A; S100A9;GBP1
Degradation Of Extracellular Matrix R-HSA-1474228	0,024489715246689445	COL17A1;MMP19;CTSG;MMP8; MMP9;ELANE;COL19A1
Creation Of C4 And C2 Activators R-HSA-166786	0,025892172149261056	C1QB;C1QA;C1QC
Activation Of Matrix Metalloproteinases R-HSA-1592389	0,03019893550877477	CTSG;MMP8;MMP9;ELANE

Collagen Formation R-HSA-1474290	0,04070582565500134	COL17A1;ADAMTS2;PCOLCE2; COL4A3;MMP9;COL19A1

Table S.12. Reactome Pathway enrichment analysis of the 185-gene signature differentiating from active TB vs. Contacts

Term	Adjusted P-value	Genes
Neutrophil Degranulation R-HSA-6798695	2,33E+03	SERPINB10;ORM1;CRISP3;GPR84;MPO;ABCA13; CLEC5A;S100A12;OLR1;PGLYRP1;ELANE;CD177; DEFA4;MCEMP1;AZU1;OLFM4;MMP8;MMP9; CEACAM1;VNN1;TCN1;CEACAM6;LCN2;PRTN3; BPI;CEACAM8;LTF
Innate Immune System R-HSA-168249	7,68E+03	SERPINB10;C1QB;C1QA;ORM1;CRISP3;GPR84; MPO;ABCA13;MUC1;CLEC5A;S100A12;OLR1; PGLYRP1;FCGR1A;ELANE;CD177;MYO10;DEFA4; MCEMP1;AZU1;OLFM4;MMP8;MMP9;CEACAM1; GZMM;VNN1;AIM2;TCN1;CEACAM6;LCN2;PRTN3; BPI;CEACAM8;DOCK1;LTF;C1QC
Immune System R-HSA-168256	1,21E+08	SERPINB10;C1QB;CD274;C1QA;ORM1;CRISP3; GPR84;MPO;ABCA13;LILRA4;MUC1;CLEC5A; S100A12;OLR1;PGLYRP1;FCGR1A;GBP1;ELANE; CD177;GBP6;GBP5;MYO10;DEFA4;MCEMP1;AZU1 ;PDCD1LG2;OLFM4;MMP8;MMP9;CEACAM1;GZMM; VNN1;AIM2;TCN1;CEACAM6;LCN2;SDC1;PRTN3;BP I; CEACAM8;FGF13;DOCK1;LTF;C1QC
Extracellular Matrix Organization R-HSA-1474244	1,32E+09	COL17A1;COLGALT2;PCOLCE2;NTN4;MMP8; FBLN2;MMP9;VCAN;CEACAM1;CEACAM6;SDC1; MMP19;ITGA7;CEACAM8;FGF13;ELANE
Antimicrobial Peptides R-HSA-6803157	4,50E+12	DEFA4;LCN2;BPI;PRTN3;PGLYRP1;ELANE;LTF
Fibronectin Matrix Formation R-HSA-1566977	4,50E+12	CEACAM1;CEACAM6;CEACAM8
Classical Antibody-Mediated Complement Activation R-HSA-173623	4,50E+12	C1QB;C1QA;C1QC
Collagen Degradation R-HSA-1442490	6,44E+11	COL17A1;MMP19;MMP8;MMP9;ELANE
Initial Triggering Of Complement R-HSA-166663	7,53E+11	C1QB;C1QA;GZMM;C1QC

Complement Cascade R-HSA-166658	0,002473092629635788	C1QB;C1QA;GZMM;ELANE;C1QC
Creation Of C4 And C2 Activators R-HSA-166786	0,004971298281994649	C1QB;C1QA;C1QC
Regulation Of Complement Cascade R-HSA-977606	0,012102519789224536	C1QB;C1QA;ELANE;C1QC
Common Pathway Of Fibrin Clot Formation R-HSA-140875	0,016808796522291627	PRTN3;F5;CD177
Cell Surface Interactions At Vascular Wall R-HSA-202733	0,016808796522291627	CEACAM1;CEACAM6;SDC1;OLR1;CEACAM8;CD177
Metal Sequestration By Antimicrobial Proteins R-HSA-6799990	0,01986523050810356	LCN2;LTF
Degradation Of Extracellular Matrix R-HSA-1474228	0,03673369165814672	COL17A1;MMP19;MMP8;MMP9;ELANE
Activation Of Matrix Metalloproteinases R-HSA-1592389	0,0431335296967733	MMP8;MMP9;ELANE

Tabel S.13. Reactome Pathway enrichment analysis of the 81 common TB signatures

Term	Adjusted P-value	Genes
Neutrophil Degranulation R-HSA-6798695	1,16E-03	SERPINB10;CRISP3;GPR84;MPO;ABCA13;OLR1;S100A12;PGLYRP1;ELANE;CD177;DEFA4;MCEMP1;AZU1;MMP8;OLFM4;MMP9;CEACAM1;VNN1;CEACAM6;TCN1;LCN2;PRTN3;BPI;CEACAM8;LTF
Innate Immune System R-HSA-168249	6,51E-02	C1QB;SERPINB10;C1QA;CRISP3;GPR84;MPO;ABCA13;OLR1;S100A12;PGLYRP1;FCGR1A;ELANE;CD177;DEFA4;MCEMP1;AZU1;MMP8;OLFM4;MMP9;CEACAM1;AIM2;VNN1;CEACAM6;TCN1;LCN2;PRTN3;BPI;CEACAM8;LTF;C1QC
Immune System R-	8,99E-01	C1QB;SERPINB10;CD274;C1QA;CRISP3;GPR84;MPO;ABCA13;OLR1;S100A12;PGLYRP1;FCGR1A;GBP1;ELANE;CD177;GBP6;GBP5;DEFA4;MCEMP1;PDCD1LG2;AZU1;MMP8;OLFM4;MMP9;

HSA-168256		CEACAM1;AIM2;VNN1;CEACAM6;TCN1;LCN2;SDC1;PRTN3;BPI;CEA:CAM8;FGF13;LTF;C1QC
Extracellular Matrix Organization R-HSA-1474244	9,99E+08	COL17A1;CEACAM1;PCOLCE2;CEACAM6;ITGA7;SDC1;CEACAM8;MMP8;FGF13;MMP9;ELANE
Antimicrobial Peptides R-HSA-6803157	2,65E+10	DEFA4;LCN2;PRTN3;BPI;PGLYRP1;ELANE;LTF
Fibronectin Matrix Formation R-HSA-1566977	3,85E+10	CEACAM1;CEACAM6;CEACAM8
Classical Antibody-Mediated Complement Activation R-HSA-173623	3,85E+10	C1QB;C1QA;C1QC
Cell Surface Interactions At Vascular Wall R-HSA-202733	4,16E+12	CEACAM1;CEACAM6;OLR1;SDC1;CEACAM8;CD177
Collagen Degradation R-HSA-1442490	4,65E+11	COL17A1;MMP8;MMP9;ELANE
Creation Of C4 And C2 Activators R-HSA-166786	4,79E+12	C1QB;C1QA;C1QC
Regulation Of Complement Cascade R-HSA-977606	6,11E+11	C1QB;C1QA;ELANE;C1QC
Complement Cascade R-HSA-166658	0,0012449064411284157	C1QB;C1QA;ELANE;C1QC
Initial Triggering Of	0,001320831915130857	C1QB;C1QA;C1QC

Complement R-HSA-166663		
Metal Sequestration By Antimicrobial Proteins R-HSA-6799990	0,0037902462614644306	LCN2;LTF
Activation Of Matrix Metalloproteinases R-HSA-1592389	0,004540448653509372	MMP8;MMP9;ELANE
Cytokine Signaling In Immune System R-HSA-1280215	0,005691184402038079	GBP6;GBP5;LCN2;S100A12;SDC1;PRTN3;FCGR1A;FGF13;MMP9;GBP1
Interferon Gamma Signaling R-HSA-877300	0,005691184402038079	GBP6;GBP5;FCGR1A;GBP1
Degradation Of Extracellular Matrix R-HSA-1474228	0,011547534204165127	COL17A1;MMP8;MMP9;ELANE
Syndecan Interactions R-HSA-3000170	0,03414545960954582	SDC1;FGF13
PD-1 Signaling R-HSA-389948	0,03576237616367435	CD274;PDCD1LG2
Common Pathway Of Fibrin Clot Formation R-HSA-140875	0,037370951568227055	PRTN3;CD177
Other Interleukin Signaling R-HSA-449836	0,04240700298899722	SDC1;PRTN3

Tabel S.13: Reactome Pathway enrichment analysis of the 126-gene signature differentiating from contact vs. Control from singapoure

Term	Adjusted P-value	Genes
Interferon Alpha/Beta Signaling R-HSA-909733	0,006528396	IFITM3;IFITM2;IRF7;IFIT2;OASL
Platelet Degranulation R-HSA-114608	0,006528396	SPARC;ITGA2B;FN1;PPBP;CLU;PF4
Response To Elevated Platelet Cytosolic Ca2+ R-HSA-76005	0,006528396	SPARC;ITGA2B;FN1;PPBP;CLU;PF4
Platelet Activation, Signaling And Aggregation R-HSA-76002	0,030007965	GP9;SPARC;ITGA2B;FN1;PPBP;CLU;PF4
Formyl Peptide Receptors Bind Formyl Peptides And Many Other Ligands R-HSA-444473	0,036396332	FPR1;FPR2
GPCR Ligand Binding R-HSA-500792	0,036396332	HCAR2;CXCR1;FZD6;FPR1;ADM;FFAR2;FPR2;PPBP;PF4
Platelet Aggregation (Plug Formation) R-HSA-76009	0,036858899	GP9;ITGA2B;FN1
Immune System R-HSA-168256	0,04540071	IFITM3;IL1RN;IFITM2;TNFAIP6;IL1R2;HSPA6;FPR1;FN1;FPR2;PPBP; CLU;DNM1;IFIT2;OASL;ADGRG3;TREML1;LRG1;CXCR1;CLEC7A;IRF7; DOCK1
Interleukin-10 Signaling R-HSA-6783783	0,04540071	IL1RN;IL1R2;FPR1

Axon Guidance R-HSA-422475	0,04540071	NELL2;DOK6;ITGA2B;NRCAM;RPS4Y1;MYL9;DOCK1;SCN1B;DNM1
Class A/1 (Rhodopsin-like Receptors) R-HSA-373076	0,04540071	HCAR2;CXCR1;FPR1;FFAR2;FPR2;PPBP;PF4
L1CAM Interactions R-HSA-373760	0,04540071	ITGA2B;NRCAM;SCN1B;DNM1

Table S.14. 99 important features which discriminated TB patients from controls and are ranked based on prediction power

Rank	Ensemble ID	import ance	symbol
**1**	ENSG00000168062	0,12038589	BATF2
**2**	ENSG00000108950	0,06468799	FAM20A
**3**	ENSG00000163520	0,044113334	FBLN2
**4**	ENSG00000154027	0,03994267	AK5
**5**	ENSG00000168899	0,037877075	VAMP5
**6**	ENSG00000118113	0,03396286	MMP8
**7**	ENSG00000185909	0,031269636	KLHDC8B
**8**	ENSG00000235532	0,030086495	LINC00402
**9**	ENSG00000239839	0,026672747	DEFA3
**10**	ENSG00000183347	0,02400389	GBP6
**11**	ENSG00000255221	0,02276823	Novel transcript
**12**	ENSG00000177989	0,021645801	ODF3B
**13**	ENSG00000275395	0,018501336	FCGBP
**14**	ENSG00000175894	0,016561806	TSPEAR
**15**	ENSG00000082014	0,015522415	SMARCD3
**16**	ENSG00000112394	0,015021088	SLC16A10
**17**	ENSG00000144290	0,014799505	SLC4A10
**18**	ENSG00000152894	0,014391645	PTPRK
**19**	ENSG00000174469	0,014215433	CNTNAP2
**20**	ENSG00000156738	0,014006364	MS4A1
**21**	ENSG00000168528	0,013855372	SERINC2
**22**	ENSG00000188820	0,013704887	CALHM6
**23**	ENSG00000258732	0,013637463	Novel transcript
**24**	ENSG00000133424	0,013465438	LARGE1

**25**	ENSG00000138772	0,01298144	ANXA3
**26**	ENSG00000291135	0,012662154	FCGR1BP
**27**	ENSG00000214652	0,012551758	ZNF727
**28**	ENSG00000074660	0,012307815	SCARF1
**29**	ENSG00000278897	0,011515373	Novel transcript
**30**	ENSG00000184613	0,010823427	NELL2
**31**	ENSG00000206077	0,010216486	ZDHHC11B
**32**	ENSG00000133063	0,010141966	CHIT1
**33**	ENSG00000249992	0,008343413	TMEM158
**34**	ENSG00000289278	0,00834127	Novel transcript
**35**	ENSG00000145685	0,008182268	LHFPL2
**36**	ENSG00000197520	0,007952745	FAM177B
**37**	ENSG00000143416	0,007853908	SELENBP1
**38**	ENSG00000134321	0,007663899	RSAD2
**39**	ENSG00000128536	0,007336232	CDHR3
**40**	ENSG00000169031	0,007331068	COL4A3
**41**	ENSG00000255733	0,00710369	IFNG-AS1
**42**	ENSG00000088340	0,007059258	FER1L4
**43**	ENSG00000112486	0,007015528	CCR6
**44**	ENSG00000106070	0,006843952	GRB10
**45**	ENSG00000124785	0,006291078	NRN1
**46**	ENSG00000100095	0,006066543	SEZ6L
**47**	ENSG00000198959	0,006016463	TGM2
**48**	ENSG00000135116	0,005355277	HRK
**49**	ENSG00000164330	0,005337989	EBF1
**50**	ENSG00000158578	0,004923568	ALAS2
**51**	ENSG00000224940	0,004875258	PRRT4
**52**	ENSG00000132185	0,004425741	FCRLA
**53**	ENSG00000215630	0,004121511	GUSBP9
**54**	ENSG00000187244	0,003913632	BCAM
**55**	ENSG00000173369	0,003883552	C1QB
**56**	ENSG00000211821	0,003863258	TRDV2
**57**	ENSG00000196415	0,003840864	PRTN3
**58**	ENSG00000169397	0,00378118	RNASE3
**59**	ENSG00000214548	0,003624287	MEG3
**60**	ENSG00000152229	0,003545072	PSTPIP2
**61**	ENSG00000258511	0,003519075	Novel transcript
**62**	ENSG00000257017	0,003298582	HP
**63**	ENSG00000198753	0,003241712	PLXNB3
**64**	ENSG00000134545	0,003213379	KLRC1
**65**	ENSG00000075399	0,00307871	VPS9D1
**66**	ENSG00000163814	0,003001559	CDCP1
**67**	ENSG00000248385	0,002914369	TARM1
**68**	ENSG00000130202	0,00282182	NECTIN2

**69**	ENSG00000213557	0,002802352	RPL31P43
**70**	ENSG00000172264	0,002643851	MACROD2
**71**	ENSG00000087237	0,002638522	CETP
**72**	ENSG00000173114	0,00258439	LRRN3
**73**	ENSG00000186081	0,002434945	KRT5
**74**	ENSG00000113088	0,002351718	GZMK
**75**	ENSG00000234184	0,00233618	LINC01781
**76**	ENSG00000143546	0,002016288	S100A8
**77**	ENSG00000157168	0,00186084	NRG1
**78**	ENSG00000151320	0,001845652	AKAP6
**79**	ENSG00000242550	0,00177784	SERPINB10
**80**	ENSG00000167680	0,001773362	SEMA6B
**81**	ENSG00000142089	0,00171878	IFITM3
**82**	ENSG00000163958	0,001543567	ZDHHC19
**83**	ENSG00000115884	0,001465165	SDC1
**84**	ENSG00000197561	0,00111243	ELANE
**85**	ENSG00000211806	0,001017811	TRAV25
**86**	ENSG00000185897	0,000988955	FFAR3
**87**	ENSG00000204936	0,000930304	CD177
**88**	ENSG00000173585	0,000888983	CCR9
**89**	ENSG00000223609	0,000748731	HBD
**90**	ENSG00000206177	0,000703684	HBM
**91**	ENSG00000197353	0,000685512	LYPD2
**92**	ENSG00000233030	0,000600206	LOC124904411
**93**	ENSG00000284194	0,000574073	SCO2
**94**	ENSG00000172232	0,000498183	AZU1
**95**	ENSG00000122861	0,000395337	PLAU
**96**	ENSG00000008438	0,000360496	PGLYRP1
**97**	ENSG00000101883	0,000276182	RHOXF1
**98**	ENSG00000120217	7,90E+01	CD274
**99**	ENSG00000183960	6,52E+01	KCNH8

Tabel S.15: Reactome Pathway enrichment analysis of 99 signature

Term	**Adjusted P-value**	**Genes**
Neutrophil Degranulation R-HSA-6798695	0,0005447	CHIT1;SERPINB10;PLAU;TARM1;PRTN3;RNASE3;AZU1; PGLYRP1;MMP8;S100A8;ELANE;CD177

Antimicrobial Peptides R-HSA-6803157	0,0005447	PRTN3;CCR6;RNASE3;PGLYRP1;S100A8;ELANE
Immune System R-HSA-168256	0,0025981	IFITM3;GBP6;C1QB;SERPINB10;CD274;RSAD2;KCNH8; RNASE3;AZU1;MMP8;CHIT1;PLAU;TARM1;GRB10;SDC1; PRTN3;CCR6;KLRC1;PGLYRP1;S100A8; ELANE;NECTIN2;CD177
Innate Immune System R-HSA-168249	0,0068623	C1QB;SERPINB10;KCNH8;RNASE3;AZU1;MMP8;CHIT1; PLAU;TARM1;PRTN3; CCR6;PGLYRP1;S100A8;ELANE;CD177

Table S.16. 16 gene sets that were significantly enriched in TB-like group

**Gene Set NAME**	**ES**	**NES**	**NOM p-val**	**FDR q-val**
P53_PATHWAY	0,41717315	1,7414215	0,004056795	0,16981064
IL6_JAK_STAT3_SIGNALING	0,5101017	1,696835	0,008179959	0,14531085
INTERFERON_GAMMA_RESPONSE	0,6184512	1,6896951	0,03909465	0,1142068
TNFA_SIGNALING_VIA_NFKB	0,5550211	1,6664759	0,019736841	0,11176852
REACTIVE_OXYGEN_SPECIES_PATHWAY	0,47925183	1,6587615	0,03929273	0,097650416
INTERFERON_ALPHA_RESPONSE	0,73531544	1,630852	0,020876827	0,097962424
INFLAMMATORY_RESPONSE	0,48792702	1,5773038	0,024282562	0,12947103
COMPLEMENT	0,40425995	1,5567251	0,0125	0,13145365
PI3K_AKT_MTOR_SIGNALING	0,2876529	1,5233263	0,01934236	0,14582542
UV_RESPONSE_UP	0,34995183	1,4800696	0,032989692	0,17354906
ESTROGEN_RESPONSE_LATE	0,41273728	1,4688157	0,015765766	0,17135732
COAGULATION	0,44793665	1,4373039	0,016161617	0,19385085
ESTROGEN_RESPONSE_EARLY	0,36938202	1,4059856	0,017582418	0,19912939
HYPOXIA	0,3657321	1,3806304	0,04883227	0,21439889
XENOBIOTIC_METABOLISM	0,3446735	1,3670818	0,022633744	0,21407826
APICAL_JUNCTION	0,36421877	1,3217614	0,04506438	0,2416481

Table S.17. Gene symbols and identifiers included in the IL6_JAK_STAT3_SIGNALING gene set

Gene stable ID	Gene name
**ENSG00000292332**	IL3RA
**ENSG00000292363**	CRLF2
**ENSG00000292357**	CSF2RA
**ENSG00000292373**	IL9R
**ENSG00000175354**	PTPN2
**ENSG00000285048**	IRF9
**ENSG00000243646**	IL10RB
**ENSG00000159128**	IFNGR2
**ENSG00000107447**	DNTT
**ENSG00000142166**	IFNAR1
**ENSG00000100292**	HMOX1
**ENSG00000164400**	CSF2
**ENSG00000196396**	PTPN1
**ENSG00000135218**	CD36
**ENSG00000107968**	MAP3K8
**ENSG00000115386**	REG1A
**ENSG00000100368**	CSF2RB
**ENSG00000146072**	TNFRSF21
**ENSG00000125538**	IL1B
**ENSG00000006327**	TNFRSF12A
**ENSG00000177663**	IL17RA
**ENSG00000028137**	TNFRSF1B
**ENSG00000122756**	CNTFR
**ENSG00000004468**	CD38
**ENSG00000175899**	A2M
**ENSG00000096996**	IL12RB1
**ENSG00000227507**	LTB
**ENSG00000177885**	GRB2
**ENSG00000134470**	IL15RA
**ENSG00000228978**	TNF
**ENSG00000184371**	CSF1
**ENSG00000026103**	FAS
**ENSG00000134460**	IL2RA
**ENSG00000147168**	IL2RG
**ENSG00000125347**	IRF1
**ENSG00000135503**	ACVR1B
**ENSG00000111321**	LTBR
**ENSG00000010278**	CD9

**ENSG00000138755**	CXCL9
**ENSG00000169245**	CXCL10
**ENSG00000169248**	CXCL11
**ENSG00000105329**	TGFB1
**ENSG00000156234**	CXCL13
**ENSG00000115594**	IL1R1
**ENSG00000163739**	CXCL1
**ENSG00000163737**	PF4
**ENSG00000163734**	CXCL3
**ENSG00000116678**	LEPR
**ENSG00000170458**	CD14
**ENSG00000185338**	SOCS1
**ENSG00000131724**	IL13RA1
**ENSG00000141506**	PIK3R5
**ENSG00000136244**	IL6
**ENSG00000134352**	IL6ST
**ENSG00000137193**	PIM1
**ENSG00000108688**	CCL7
**ENSG00000259207**	ITGB3
**ENSG00000027697**	IFNGR1
**ENSG00000184557**	SOCS3
**ENSG00000163823**	CCR1
**ENSG00000179295**	PTPN11
**ENSG00000077238**	IL4R
**ENSG00000115604**	IL18R1
**ENSG00000056736**	IL17RB
**ENSG00000115232**	ITGA4
**ENSG00000105246**	EBI3
**ENSG00000139269**	INHBE
**ENSG00000026508**	CD44
**ENSG00000145623**	OSMR
**ENSG00000170581**	STAT2
**ENSG00000067182**	TNFRSF1A
**ENSG00000115590**	IL1R2
**ENSG00000145431**	PDGFC
**ENSG00000030110**	BAK1
**ENSG00000137462**	TLR2
**ENSG00000139567**	ACVRL1
**ENSG00000110395**	CBL
**ENSG00000115415**	STAT1
**ENSG00000168610**	STAT3
**ENSG00000105397**	TYK2
**ENSG00000115145**	STAM2
**ENSG00000104432**	IL7
**ENSG00000172936**	MYD88

**ENSG00000143575**	HAX1
**ENSG00000188257**	PLA2G2A
**ENSG00000177606**	JUN
**ENSG00000119535**	CSF3R

Table S.18. Gene symbols and identifiers included in the INTERFERON_GAMMA_RESPONSE gene set

Gene stable ID	Gene name
ENSG00000288512	C1R
ENSG00000284916	PSME1
ENSG00000284889	PSME2
ENSG00000285152	RNF31
ENSG00000175354	PTPN2
ENSG00000285048	IRF9
ENSG00000133106	EPSTI1
ENSG00000124151	NCOA3
ENSG00000150457	LATS2
ENSG00000125245	GPR18
ENSG00000291555	RTP4
ENSG00000157601	MX1
ENSG00000183486	MX2
ENSG00000291814	PNP
ENSG00000122644	ARL4A
ENSG00000106100	NOD1
ENSG00000060491	OGFR

ENSG00000159110	IFNAR2
ENSG00000276561	IRF7
ENSG00000102900	NUP93
ENSG00000153064	BANK1
ENSG00000124201	ZNFX1
ENSG00000273686	B2M
ENSG00000139626	ITGB7
ENSG00000101347	SAMHD1
ENSG00000133639	BTG1
ENSG00000275214	IFI27
ENSG00000196396	PTPN1
ENSG00000131979	GCH1
ENSG00000145649	GZMA
ENSG00000113532	ST8SIA4
ENSG00000096968	JAK2
ENSG00000174600	CMKLR1
ENSG00000125148	MT2A
ENSG00000138035	PNPT1
ENSG00000100567	PSMA3
ENSG00000059378	PARP12
ENSG00000163412	EIF4E3

ENSG00000274233	CCL5
ENSG00000068079	IFI35
ENSG00000221963	APOL6
ENSG00000185745	IFIT1
ENSG00000100368	CSF2RB
ENSG00000164136	IL15
ENSG00000172331	BPGM
ENSG00000119917	IFIT3
ENSG00000124256	ZBP1
ENSG00000150347	ARID5B
ENSG00000101017	CD40
ENSG00000119922	IFIT2
ENSG00000173193	PARP14
ENSG00000121858	TNFSF10
ENSG00000125826	RBCK1
ENSG00000168062	BATF2
ENSG00000130589	HELZ2
ENSG00000141574	SECTM1
ENSG00000004468	CD38
ENSG00000126067	PSMB2
ENSG00000043462	LCP2

ENSG00000106588	PSMA2
ENSG00000100644	HIF1A
ENSG00000158987	RAPGEF6
ENSG00000090339	ICAM1
ENSG00000127951	FGL2
ENSG00000216490	IFI30
ENSG00000111679	PTPN6
ENSG00000165806	CASP7
ENSG00000204257	HLA-DMA
ENSG00000134470	IL15RA
ENSG00000278032	LY6E
ENSG00000276051	HLA-G
ENSG00000105835	NAMPT
ENSG00000026103	FAS
ENSG00000227816	TAP1
ENSG00000197329	PELI1
ENSG00000230669	PSMB8
ENSG00000137275	RIPK1
ENSG00000143184	XCL1
ENSG00000125347	IRF1
ENSG00000137200	CMTR1

ENSG00000137628	DDX60
ENSG00000229215	HLA-A
ENSG00000243958	PSMB9
ENSG00000228881	TRIM26
ENSG00000075142	SRI
ENSG00000184979	USP18
ENSG00000066583	ISOC1
ENSG00000067057	PFKP
ENSG00000155287	SLC25A28
ENSG00000228080	HLA-DRB1
ENSG00000116711	PLA2G4A
ENSG00000174175	SELP
ENSG00000236418	HLA-DQA1
ENSG00000156587	UBE2L6
ENSG00000228964	HLA-B
ENSG00000149131	SERPING1
ENSG00000241534	CFB
ENSG00000138755	CXCL9
ENSG00000169245	CXCL10
ENSG00000169248	CXCL11
ENSG00000121060	TRIM25

ENSG00000100385	IL2RB
ENSG00000112493	TAPBP
ENSG00000188313	PLSCR1
ENSG00000120217	CD274
ENSG00000136816	TOR1B
ENSG00000142089	IFITM3
ENSG00000138642	HERC6
ENSG00000130303	BST2
ENSG00000196116	TDRD7
ENSG00000106785	TRIM14
ENSG00000140464	PML
ENSG00000019582	CD74
ENSG00000145416	MARCHF1
ENSG00000100906	NFKBIA
ENSG00000168310	IRF2
ENSG00000185338	SOCS1
ENSG00000140105	WARS1
ENSG00000002549	LAP3
ENSG00000111331	OAS3
ENSG00000111335	OAS2
ENSG00000065911	MTHFD2

ENSG00000196954	CASP4
ENSG00000108679	LGALS3BP
ENSG00000136244	IL6
ENSG00000183696	UPP1
ENSG00000013364	MVP
ENSG00000109320	NFKB1
ENSG00000108771	DHX58
ENSG00000177409	SAMD9L
ENSG00000158321	AUTS2
ENSG00000164305	CASP3
ENSG00000128604	IRF5
ENSG00000140853	NLRC5
ENSG00000055332	EIF2AK2
ENSG00000134321	RSAD2
ENSG00000123609	NMI
ENSG00000123610	TNFAIP6
ENSG00000179583	CIITA
ENSG00000135148	TRAFD1
ENSG00000137193	PIM1
ENSG00000108688	CCL7
ENSG00000108691	CCL2

ENSG00000205220	PSMB10
ENSG00000137265	IRF4
ENSG00000140280	LYSMD2
ENSG00000185215	TNFAIP2
ENSG00000184557	SOCS3
ENSG00000170439	TMT1B
ENSG00000140968	IRF8
ENSG00000077238	IL4R
ENSG00000107201	RIGI
ENSG00000134326	CMPK2
ENSG00000110848	CD69
ENSG00000172183	ISG20
ENSG00000291237	SOD2
ENSG00000174944	P2RY14
ENSG00000170581	STAT2
ENSG00000185201	IFITM2
ENSG00000182326	C1S
ENSG00000162654	GBP4
ENSG00000137959	IFI44L
ENSG00000073756	PTGS2
ENSG00000187608	ISG15

ENSG00000137965	IFI44
ENSG00000171051	FPR1
ENSG00000114013	CD86
ENSG00000118503	TNFAIP3
ENSG00000138600	SPPL2A
ENSG00000150337	FCGR1A
ENSG00000131203	IDO1
ENSG00000137752	CASP1
ENSG00000118640	VAMP8
ENSG00000168899	VAMP5
ENSG00000173821	RNF213
ENSG00000110324	IL10RA
ENSG00000064012	CASP8
ENSG00000123096	SSPN
ENSG00000137496	IL18BP
ENSG00000115267	IFIH1
ENSG00000213809	KLRK1
ENSG00000135114	OASL
ENSG00000115525	ST3GAL5
ENSG00000104312	RIPK2
ENSG00000135899	SP110

ENSG00000115415	STAT1
ENSG00000168610	STAT3
ENSG00000132530	XAF1
ENSG00000265972	TXNIP
ENSG00000124762	CDKN1A
ENSG00000132109	TRIM21
ENSG00000104432	IL7
ENSG00000172936	MYD88
ENSG00000138378	STAT4
ENSG00000184588	PDE4B
ENSG00000000971	CFH
ENSG00000183347	GBP6
ENSG00000160710	ADAR
ENSG00000026751	SLAMF7
ENSG00000162692	VCAM1

Table S.19. Gene symbols and identifiers included in the TNFA_SIGNALING_VIA_NFKB gene set

Gene stable ID	Gene name
ENSG00000281165	SLC2A6
ENSG00000185950	IRS2
ENSG00000157557	ETS2
ENSG00000125845	BMP2
ENSG00000154640	BTG3
ENSG00000169508	GPR183
ENSG00000124225	PMEPA1
ENSG00000177426	TGIF1
ENSG00000158470	B4GALT5
ENSG00000128342	LIF
ENSG00000124145	SDC4
ENSG00000067082	KLF6
ENSG00000142178	SIK1
ENSG00000284099	SQSTM1
ENSG00000159128	IFNGR2
ENSG00000102804	TSC22D1
ENSG00000131669	NINJ1
ENSG00000173391	OLR1

ENSG00000160223	ICOSLG
ENSG00000274538	PHLDA2
ENSG00000159200	RCAN1
ENSG00000136826	KLF4
ENSG00000124875	CXCL6
ENSG00000168685	IL7R
ENSG00000179431	FJX1
ENSG00000277943	CCL4
ENSG00000133639	BTG1
ENSG00000106366	SERPINE1
ENSG00000164400	CSF2
ENSG00000122861	PLAU
ENSG00000131979	GCH1
ENSG00000056558	TRAF1
ENSG00000107968	MAP3K8
ENSG00000138166	DUSP5
ENSG00000197632	SERPINB2
ENSG00000166401	SERPINB8
ENSG00000145779	TNFAIP8
ENSG00000131459	GFPT2
ENSG00000163110	PDLIM5

ENSG00000274233	CCL5
ENSG00000121594	CD80
ENSG00000277443	MARCKS
ENSG00000171522	PTGER4
ENSG00000011422	PLAUR
ENSG00000125538	IL1B
ENSG00000171621	SPSB1
ENSG00000119922	IFIT2
ENSG00000184602	SNN
ENSG00000136048	DRAM1
ENSG00000176170	SPHK1
ENSG00000165029	ABCA1
ENSG00000110047	EHD1
ENSG00000185022	MAFF
ENSG00000125657	TNFSF9
ENSG00000125733	TRIP10
ENSG00000130066	SAT1
ENSG00000162407	PLPP3
ENSG00000136560	TANK
ENSG00000144476	ACKR3
ENSG00000122877	EGR2

ENSG00000175592	FOSL1
ENSG00000090339	ICAM1
ENSG00000122641	INHBA
ENSG00000163661	PTX3
ENSG00000163660	CCNL1
ENSG00000119138	KLF9
ENSG00000117525	F3
ENSG00000101384	JAG1
ENSG00000134470	IL15RA
ENSG00000228978	TNF
ENSG00000105835	NAMPT
ENSG00000184371	CSF1
ENSG00000227816	TAP1
ENSG00000170525	PFKFB3
ENSG00000172216	CEBPB
ENSG00000120738	EGR1
ENSG00000077150	NFKB2
ENSG00000125347	IRF1
ENSG00000134107	BHLHE40
ENSG00000175505	CLCF1
ENSG00000115009	CCL20

ENSG00000119508	NR4A3
ENSG00000235030	IER3
ENSG00000145632	PLK2
ENSG00000123358	NR4A1
ENSG00000132510	KDM6B
ENSG00000171223	JUNB
ENSG00000137267	TUBB2A
ENSG00000126561	STAT5A
ENSG00000196878	LAMB3
ENSG00000081041	CXCL2
ENSG00000169245	CXCL10
ENSG00000169248	CXCL11
ENSG00000163874	ZC3H12A
ENSG00000169242	EFNA1
ENSG00000164251	F2RL1
ENSG00000120129	DUSP1
ENSG00000163739	CXCL1
ENSG00000163734	CXCL3
ENSG00000184014	DENND5A
ENSG00000100906	NFKBIA
ENSG00000143878	RHOB

ENSG00000113302	IL12B
ENSG00000179388	EGR3
ENSG00000109321	AREG
ENSG00000132334	PTPRE
ENSG00000173812	EIF1
ENSG00000136244	IL6
ENSG00000168884	TNIP2
ENSG00000109320	NFKB1
ENSG00000041982	TNC
ENSG00000110944	IL23A
ENSG00000164949	GEM
ENSG00000108932	SLC16A6
ENSG00000134352	IL6ST
ENSG00000170345	FOS
ENSG00000142871	CCN1
ENSG00000123610	TNFAIP6
ENSG00000108691	CCL2
ENSG00000185215	TNFAIP2
ENSG00000184557	SOCS3
ENSG00000121797	CCRL2
ENSG00000107201	RIGI

ENSG00000140379	BCL2A1
ENSG00000113070	HBEGF
ENSG00000059728	MXD1
ENSG00000143384	MCL1
ENSG00000086062	B4GALT1
ENSG00000165030	NFIL3
ENSG00000110848	CD69
ENSG00000102908	NFAT5
ENSG00000291237	SOD2
ENSG00000026508	CD44
ENSG00000115738	ID2
ENSG00000110218	PANX1
ENSG00000116514	RNF19B
ENSG00000112715	VEGFA
ENSG00000145901	TNIP1
ENSG00000003402	CFLAR
ENSG00000073756	PTGS2
ENSG00000118515	SGK1
ENSG00000110092	CCND1
ENSG00000070961	ATP2B1
ENSG00000137462	TLR2

ENSG00000155090	KLF10
ENSG00000139289	PHLDA1
ENSG00000104856	RELB
ENSG00000118503	TNFAIP3
ENSG00000087074	PPP1R15A
ENSG00000196371	FUT4
ENSG00000189067	LITAF
ENSG00000173039	RELA
ENSG00000069399	BCL3
ENSG00000059804	SLC2A3
ENSG00000078401	EDN1
ENSG00000112149	CD83
ENSG00000162783	IER5
ENSG00000115267	IFIH1
ENSG00000115008	IL1A
ENSG00000115919	KYNU
ENSG00000104312	RIPK2
ENSG00000075426	FOSL2
ENSG00000221869	CEBPD
ENSG00000125740	FOSB
ENSG00000166949	SMAD3

ENSG00000162924	REL
ENSG00000130164	LDLR
ENSG00000116044	NFE2L2
ENSG00000034152	MAP2K3
ENSG00000128016	ZFP36
ENSG00000127528	KLF2
ENSG00000153234	NR4A2
ENSG00000179094	PER1
ENSG00000115956	PLEK
ENSG00000099860	GADD45B
ENSG00000146278	PNRC1
ENSG00000124762	CDKN1A
ENSG00000120875	DUSP4
ENSG00000178860	MSC
ENSG00000023445	BIRC3
ENSG00000136997	MYC
ENSG00000110330	BIRC2
ENSG00000146232	NFKBIE
ENSG00000205189	ZBTB10
ENSG00000113916	BCL6
ENSG00000160888	IER2

ENSG00000173334	TRIB1
ENSG00000184588	PDE4B
ENSG00000163659	TIPARP
ENSG00000114315	HES1
ENSG00000123689	G0S2
ENSG00000158050	DUSP2
ENSG00000150782	IL18
ENSG00000116717	GADD45A
ENSG00000049249	TNFRSF9
ENSG00000162616	DNAJB4
ENSG00000159388	BTG2
ENSG00000177606	JUN
ENSG00000196449	YRDC
ENSG00000162772	ATF3

Table S.20. Gene symbols and identifiers included in the INTERFERON_ALPHA_RESPONSE gene set

Gene stable ID	Gene name
ENSG00000284916	PSME1
ENSG00000284889	PSME2
ENSG00000285152	RNF31
ENSG00000285048	IRF9
ENSG00000133106	EPSTI1
ENSG00000139679	LPAR6
ENSG00000291555	RTP4
ENSG00000101000	PROCR
ENSG00000157601	MX1
ENSG00000060491	OGFR
ENSG00000276561	IRF7
ENSG00000273686	B2M
ENSG00000275214	IFI27
ENSG00000120690	ELF1
ENSG00000111912	NCOA7
ENSG00000112773	TENT5A
ENSG00000138035	PNPT1

ENSG00000100567	PSMA3
ENSG00000059378	PARP12
ENSG00000068079	IFI35
ENSG00000164136	IL15
ENSG00000119917	IFIT3
ENSG00000119922	IFIT2
ENSG00000173193	PARP14
ENSG00000168062	BATF2
ENSG00000130589	HELZ2
ENSG00000137198	GMPR
ENSG00000216490	IFI30
ENSG00000278032	LY6E
ENSG00000184371	CSF1
ENSG00000227816	TAP1
ENSG00000230669	PSMB8
ENSG00000013374	NUB1
ENSG00000125347	IRF1
ENSG00000137200	CMTR1
ENSG00000137628	DDX60
ENSG00000188404	SELL

ENSG00000243958	PSMB9
ENSG00000228881	TRIM26
ENSG00000184979	USP18
ENSG00000155287	SLC25A28
ENSG00000146859	TMEM140
ENSG00000206435	HLA-C
ENSG00000156587	UBE2L6
ENSG00000169245	CXCL10
ENSG00000169248	CXCL11
ENSG00000121060	TRIM25
ENSG00000188313	PLSCR1
ENSG00000185885	IFITM1
ENSG00000142089	IFITM3
ENSG00000138642	HERC6
ENSG00000130303	BST2
ENSG00000196116	TDRD7
ENSG00000106785	TRIM14
ENSG00000019582	CD74
ENSG00000168310	IRF2
ENSG00000140105	WARS1

ENSG00000002549	LAP3
ENSG00000089127	OAS1
ENSG00000108679	LGALS3BP
ENSG00000132256	TRIM5
ENSG00000173786	CNP
ENSG00000108771	DHX58
ENSG00000205413	SAMD9
ENSG00000177409	SAMD9L
ENSG00000055332	EIF2AK2
ENSG00000134321	RSAD2
ENSG00000123609	NMI
ENSG00000135148	TRAFD1
ENSG00000121797	CCRL2
ENSG00000077238	IL4R
ENSG00000134326	CMPK2
ENSG00000172183	ISG20
ENSG00000170581	STAT2
ENSG00000185201	IFITM2
ENSG00000182326	C1S
ENSG00000162645	GBP2

ENSG00000162654	GBP4
ENSG00000137959	IFI44L
ENSG00000187608	ISG15
ENSG00000137965	IFI44
ENSG00000182179	UBA7
ENSG00000138496	PARP9
ENSG00000137752	CASP1
ENSG00000064012	CASP8
ENSG00000115267	IFIH1
ENSG00000135114	OASL
ENSG00000104312	RIPK2
ENSG00000141971	MVB12A
ENSG00000135899	SP110
ENSG00000265972	TXNIP
ENSG00000196776	CD47
ENSG00000078081	LAMP3
ENSG00000132109	TRIM21
ENSG00000104432	IL7
ENSG00000155363	MOV10
ENSG00000160710	ADAR

Table S.21. Gene symbols and identifiers included in the INFLAMMATORY_RESPONSE gene set

Gene stable ID	Gene name
ENSG00000139514	SLC7A1
ENSG00000283085	TPBG
ENSG00000169508	GPR183
ENSG00000099985	OSM
ENSG00000128342	LIF
ENSG00000130706	ADRM1
ENSG00000067082	KLF6
ENSG00000291555	RTP4
ENSG00000124479	NDP
ENSG00000100739	BDKRB1
ENSG00000159128	IFNGR2
ENSG00000106546	AHR
ENSG00000105855	ITGB8
ENSG00000173391	OLR1
ENSG00000160223	ICOSLG
ENSG00000142166	IFNAR1
ENSG00000276561	IRF7

ENSG00000102265	TIMP1
ENSG00000102970	CCL17
ENSG00000102962	CCL22
ENSG00000169836	TACR3
ENSG00000124875	CXCL6
ENSG00000168685	IL7R
ENSG00000060558	GNA15
ENSG00000006210	CX3CL1
ENSG00000106366	SERPINE1
ENSG00000276336	SCARF1
ENSG00000131979	GCH1
ENSG00000174600	CMKLR1
ENSG00000136867	SLC31A2
ENSG00000274233	CCL5
ENSG00000115353	TACR1
ENSG00000164136	IL15
ENSG00000171522	PTGER4
ENSG00000011422	PLAUR
ENSG00000125538	IL1B
ENSG00000163421	PROK2

ENSG00000101017	CD40
ENSG00000258227	CLEC5A
ENSG00000146469	VIP
ENSG00000181634	TNFSF15
ENSG00000176170	SPHK1
ENSG00000197405	C5AR1
ENSG00000157227	MMP14
ENSG00000169403	PTAFR
ENSG00000121858	TNFSF10
ENSG00000165029	ABCA1
ENSG00000028137	TNFRSF1B
ENSG00000089041	P2RX7
ENSG00000125657	TNFSF9
ENSG00000137393	RNF144B
ENSG00000125726	CD70
ENSG00000175040	CHST2
ENSG00000064989	CALCRL
ENSG00000043462	LCP2
ENSG00000187764	SEMA4D
ENSG00000148926	ADM

ENSG00000100644	HIF1A
ENSG00000123700	KCNJ2
ENSG00000183484	GPR132
ENSG00000090339	ICAM1
ENSG00000105371	ICAM4
ENSG00000134070	IRAK2
ENSG00000147257	GPC3
ENSG00000167601	AXL
ENSG00000122641	INHBA
ENSG00000226979	LTA
ENSG00000117525	F3
ENSG00000136868	SLC31A1
ENSG00000134470	IL15RA
ENSG00000278032	LY6E
ENSG00000232569	GABBR1
ENSG00000105835	NAMPT
ENSG00000184371	CSF1
ENSG00000047936	ROS1
ENSG00000165168	CYBB
ENSG00000198121	LPAR1

ENSG00000171596	NMUR1
ENSG00000112818	MEP1A
ENSG00000125347	IRF1
ENSG00000007908	SELE
ENSG00000188404	SELL
ENSG00000017260	ATP2C1
ENSG00000075142	SRI
ENSG00000115009	CCL20
ENSG00000135503	ACVR1B
ENSG00000121989	ACVR2A
ENSG00000138755	CXCL9
ENSG00000169245	CXCL10
ENSG00000169248	CXCL11
ENSG00000090104	RGS1
ENSG00000056291	NPFFR2
ENSG00000100385	IL2RB
ENSG00000112493	TAPBP
ENSG00000106178	CCL24
ENSG00000164023	SGMS2
ENSG00000177105	RHOG

ENSG00000115594	IL1R1
ENSG00000175591	P2RY2
ENSG00000185885	IFITM1
ENSG00000130303	BST2
ENSG00000136754	ABI1
ENSG00000100906	NFKBIA
ENSG00000170458	CD14
ENSG00000169429	CXCL8
ENSG00000080815	PSEN1
ENSG00000113302	IL12B
ENSG00000124882	EREG
ENSG00000141506	PIK3R5
ENSG00000038945	MSR1
ENSG00000132334	PTPRE
ENSG00000105707	HPN
ENSG00000254087	LYN
ENSG00000136244	IL6
ENSG00000109320	NFKB1
ENSG00000169851	PCDH7
ENSG00000164342	TLR3

ENSG00000185245	GP1BA
ENSG00000172215	CXCR6
ENSG00000105711	SCN1B
ENSG00000110911	SLC11A2
ENSG00000125384	PTGER2
ENSG00000055332	EIF2AK2
ENSG00000174437	ATP2A2
ENSG00000123609	NMI
ENSG00000123610	TNFAIP6
ENSG00000137860	SLC28A2
ENSG00000108688	CCL7
ENSG00000108691	CCL2
ENSG00000259207	ITGB3
ENSG00000080493	SLC4A4
ENSG00000121797	CCRL2
ENSG00000170961	HAS2
ENSG00000103313	MEFV
ENSG00000077238	IL4R
ENSG00000115604	IL18R1
ENSG00000113070	HBEGF

ENSG00000059728	MXD1
ENSG00000167207	NOD2
ENSG00000105246	EBI3
ENSG00000110848	CD69
ENSG00000105976	MET
ENSG00000171860	C3AR1
ENSG00000145623	OSMR
ENSG00000134817	APLNR
ENSG00000197584	KCNMB2
ENSG00000171051	FPR1
ENSG00000070961	ATP2B1
ENSG00000196639	HRH1
ENSG00000137462	TLR2
ENSG00000057019	DCBLD2
ENSG00000170425	ADORA2B
ENSG00000172575	RASGRP1
ENSG00000115607	IL18RAP
ENSG00000110324	IL10RA
ENSG00000162493	PDPN
ENSG00000173039	RELA

ENSG00000073008	PVR
ENSG00000161638	ITGA5
ENSG00000078401	EDN1
ENSG00000174125	TLR1
ENSG00000115008	IL1A
ENSG00000132155	RAF1
ENSG00000174837	ADGRE1
ENSG00000104312	RIPK2
ENSG00000103569	AQP9
ENSG00000110436	SLC1A2
ENSG00000130164	LDLR
ENSG00000135124	P2RX4
ENSG00000108342	CSF3
ENSG00000126262	FFAR2
ENSG00000010327	STAB1
ENSG00000003989	SLC7A2
ENSG00000160013	PTGIR
ENSG00000078081	LAMP3
ENSG00000131871	SELENOS
ENSG00000124762	CDKN1A

ENSG00000082556	OPRK1
ENSG00000136997	MYC
ENSG00000126353	CCR7
ENSG00000142227	EMP3
ENSG00000085117	CD82
ENSG00000167995	BEST1
ENSG00000184588	PDE4B
ENSG00000143333	RGS16
ENSG00000019169	MARCO
ENSG00000163251	FZD5
ENSG00000136634	IL10
ENSG00000054523	KIF1B
ENSG00000150782	IL18
ENSG00000182866	LCK
ENSG00000177272	KCNA3
ENSG00000049249	TNFRSF9
ENSG00000196352	CD55
ENSG00000159388	BTG2
ENSG00000065135	GNAI3
ENSG00000119535	CSF3R

ENSG00000162711	NLRP3
ENSG00000117090	SLAMF1
ENSG00000117091	CD48

Table S.22. Gene symbols and identifiers included in the COMPLEMENT gene set

Gene stable ID	Gene name
ENSG00000288512	C1R
ENSG00000102780	DGKH
ENSG00000291527	USP16
ENSG00000100311	PDGFB
ENSG00000154655	L3MBTL4
ENSG00000187045	TMPRSS6
ENSG00000088387	DOCK9
ENSG00000057593	F7
ENSG00000126218	F10
ENSG00000128394	APOBEC3F
ENSG00000239713	APOBEC3G
ENSG00000100600	LGMN
ENSG00000291928	MSRB1
ENSG00000291971	SRC
ENSG00000173391	OLR1
ENSG00000185010	F8
ENSG00000101557	USP14
ENSG00000134871	COL4A2
ENSG00000276561	IRF7
ENSG00000102265	TIMP1
ENSG00000113088	GZMK
ENSG00000285132	CTSB
ENSG00000186472	PCLO
ENSG00000108039	XPNPEP1
ENSG00000108599	AKAP10
ENSG00000167083	GNGT2
ENSG00000214274	ANG
ENSG00000106366	SERPINE1
ENSG00000135655	USP15
ENSG00000065833	ME1
ENSG00000135218	CD36
ENSG00000277377	SERPINA1
ENSG00000145649	GZMA
ENSG00000114353	GNAI2
ENSG00000138166	DUSP5
ENSG00000096968	JAK2
ENSG00000118113	MMP8
ENSG00000197632	SERPINB2
ENSG00000103496	STX4
ENSG00000178226	PRSS36
ENSG00000111252	SH2B3
ENSG00000114423	CBLB

ENSG00000274233	CCL5
ENSG00000137714	FDX1
ENSG00000149923	PPP4C
ENSG00000011422	PLAUR
ENSG00000107798	LIPA
ENSG00000111144	LTA4H
ENSG00000087250	MT3
ENSG00000123384	LRP1
ENSG00000157227	MMP14
ENSG00000135047	CTSL
ENSG00000120049	KCNIP2
ENSG00000135678	CPM
ENSG00000110047	EHD1
ENSG00000148516	ZEB1
ENSG00000131981	LGALS3
ENSG00000160014	CALM3
ENSG00000185022	MAFF
ENSG00000102174	PHEX
ENSG00000164111	ANXA5
ENSG00000136943	CTSV
ENSG00000169896	ITGAM
ENSG00000044574	HSPA5
ENSG00000101076	HNF4A
ENSG00000167748	KLK1
ENSG00000043462	LCP2
ENSG00000058866	DGKG
ENSG00000127334	DYRK2
ENSG00000085377	PREP
ENSG00000010438	PRSS3
ENSG00000263238	CTSO
ENSG00000177556	ATOX1
ENSG00000172354	GNB2
ENSG00000165806	CASP7
ENSG00000117525	F3
ENSG00000177885	GRB2
ENSG00000204389	HSPA1A
ENSG00000100453	GZMB
ENSG00000102245	CD40LG
ENSG00000172216	CEBPB
ENSG00000114450	GNB4
ENSG00000125347	IRF1
ENSG00000096070	BRPF3
ENSG00000238196	NOTCH4
ENSG00000243958	PSMB9

ENSG00000231543	C2
ENSG00000015285	WAS
ENSG00000107485	GATA3
ENSG00000109861	CTSC
ENSG00000117984	CTSD
ENSG00000116711	PLA2G4A
ENSG00000198668	CALM1
ENSG00000149131	SERPING1
ENSG00000241534	CFB
ENSG00000186469	GNG2
ENSG00000146070	PLA2G7
ENSG00000188313	PLSCR1
ENSG00000158825	CDA
ENSG00000177105	RHOG
ENSG00000128512	DOCK4
ENSG00000137509	PRCP
ENSG00000197635	DPP4
ENSG00000282230	ADAM9
ENSG00000163739	CXCL1
ENSG00000168310	IRF2
ENSG00000005893	LAMP2
ENSG00000113600	C9
ENSG00000080815	PSEN1
ENSG00000141506	PIK3R5
ENSG00000002549	LAP3
ENSG00000196954	CASP4
ENSG00000137757	CASP5
ENSG00000130208	APOC1
ENSG00000254087	LYN
ENSG00000136244	IL6
ENSG00000115414	FN1
ENSG00000158869	FCER1G
ENSG00000197045	GMFB
ENSG00000107742	SPOCK2
ENSG00000105851	PIK3CG
ENSG00000169704	GP9
ENSG00000164951	PDP1
ENSG00000185245	GP1BA
ENSG00000164305	CASP3
ENSG00000110244	APOA4
ENSG00000085265	FCN1
ENSG00000104267	CA2
ENSG00000063978	RNF4
ENSG00000115271	GCA
ENSG00000140945	CDH13

ENSG00000138592	USP8
ENSG00000108518	PFN1
ENSG00000068796	KIF2A
ENSG00000137193	PIM1
ENSG00000121879	PIK3CA
ENSG00000164344	KLKB1
ENSG00000110851	PRDM4
ENSG00000077463	SIRT6
ENSG00000169946	ZFPM2
ENSG00000262406	MMP12
ENSG00000104112	SCG3
ENSG00000104324	CPQ
ENSG00000012223	LTF
ENSG00000102996	MMP15
ENSG00000103811	CTSH
ENSG00000116983	HPCAL4
ENSG00000137745	MMP13
ENSG00000175073	VCPIP1
ENSG00000125730	C3
ENSG00000105825	TFPI2
ENSG00000010810	FYN
ENSG00000182326	C1S
ENSG00000104368	PLAT
ENSG00000274286	ADRA2B
ENSG00000122194	PLG
ENSG00000163131	CTSS
ENSG00000104695	PPP2CB
ENSG00000117601	SERPINC1
ENSG00000176749	CDK5R1
ENSG00000118503	TNFAIP3
ENSG00000172575	RASGRP1
ENSG00000137752	CASP1
ENSG00000163932	PRKCD
ENSG00000035862	TIMP2
ENSG00000173653	RCE1
ENSG00000003400	CASP10
ENSG00000139318	DUSP6
ENSG00000132155	RAF1
ENSG00000115919	KYNU
ENSG00000115041	KCNIP3
ENSG00000135905	DOCK10
ENSG00000120885	CLU
ENSG00000085063	CD59
ENSG00000055957	ITIH1

ENSG00000164308	ERAP2
ENSG00000180210	F2
ENSG00000115956	PLEK
ENSG00000159176	CSRP1
ENSG00000047457	CP
ENSG00000198734	F5
ENSG00000115159	GPD2
ENSG00000183155	RABIF
ENSG00000173372	C1QA
ENSG00000159189	C1QC
ENSG00000117335	CD46
ENSG00000000971	CFH
ENSG00000182866	LCK
ENSG00000163220	S100A9
ENSG00000163221	S100A12
ENSG00000196352	CD55
ENSG00000117322	CR2
ENSG00000203710	CR1
ENSG00000131381	RBSN
ENSG00000077522	ACTN2
ENSG00000065135	GNAI3
ENSG00000169174	PCSK9
ENSG00000123843	C4BPB
ENSG00000132906	CASP9
ENSG00000189171	S100A13

Tabel S.23. GSEA detail for 16-gene signature of Zak et al, in control group respect to contacts

SYMBOL	RUNNING Enrichment Score	CORE ENRICHMENT
BATF2	0,01122894	No
SCARF1	0,025844958	No
FCGR1BP	0,02778495	No
GBP1	0,07491715	No
TAPBP	0,089978375	No
GBP4	0,0966631	No
APOL1	-0,062470734	No
TRAFD1	-0,157035	Yes
SERPING1	-0,14935344	Yes
GBP5	-0,11810053	Yes
STAT1	-0,10772818	Yes
ANKRD22	-0,0992964	Yes
GBP2	-0,049177557	Yes
SEPTIN4-AS1	-0,058199912	Yes
FCGR1A	-0,021695431	Yes
ETV7	0,12439683	Yes

Tabel S. 24. 450 Differentially Expressed (DE) genes between ATB and control from independent validation set

Ensemble ID	symbol	logFC	adj,P,Val
ENSG00000168062	BATF2	3,306010428	3,43413960904953e-17
ENSG00000150337	FCGR1A	2,674288657	3,43413960904953e-17
ENSG00000149131	SERPING1	3,212144611	8,24725359821702e-17
ENSG00000108861	DUSP3	1,423822796	1,51315357235232e-16
ENSG00000141574	SECTM1	1,472366188	1,93013366422085e-16
ENSG00000082014	SMARCD3	1,657456803	3,92830162482688e-16
ENSG00000162645	GBP2	1,277456168	6,60183299802915e-16
ENSG00000161955	TNFSF13	1,033774119	5,42508773452468e-15
ENSG00000074660	SCARF1	1,768850555	6,36302343681675e-15
ENSG00000177989	CIMAP1B	1,509351618	8,05244490424999e-15
ENSG00000154451	GBP5	2,105323719	8,2784804022476e-15
ENSG00000170909	OSCAR	1,112403705	1,14734757879027e-14
ENSG00000137752	CASP1	1,050835047	1,5351186261361e-14
ENSG00000075399	VPS9D1	1,322736424	2,55656680059388e-14
ENSG00000160883	HK3	1,119455918	2,63940311104661e-14
ENSG00000134243	SORT1	1,257465043	3,09286470278558e-14
ENSG00000137767	SQOR	1,037296192	3,69822714430423e-14
ENSG00000099860	GADD45B	1,010798443	4,41478330342292e-14
ENSG00000158869	FCER1G	1,29513075	4,60913424259708e-14
ENSG00000117228	GBP1	1,948588259	5,91199794017766e-14
ENSG00000025708	TYMP	1,265212294	5,91199794017766e-14
ENSG00000152229	PSTPIP2	1,545925464	9,87967110724816e-14
ENSG00000133106	EPSTI1	1,962749938	9,87967110724816e-14
ENSG00000148926	ADM	1,468057867	1,39782000238007e-13
ENSG00000183762	KREMEN1	1,98511866	1,55351475524657e-13
ENSG00000073737	DHRS9	1,509464438	2,10895077052503e-13
ENSG00000116663	FBXO6	1,656247053	2,1143652506229e-13
ENSG00000185885	IFITM1	1,31160873	2,35977311499063e-13
ENSG00000087237	CETP	1,431482844	3,23325079701511e-13
ENSG00000291135	FCGR1BP	2,245534338	4,40863431083564e-13
ENSG00000156587	UBE2L6	1,381672097	4,51976592637368e-13
ENSG00000142089	IFITM3	2,109481588	6,00558547101881e-13
ENSG00000064601	CTSA	1,024063999	6,73432766908577e-13
ENSG00000169026	SLC49A3	1,11625872	7,0709464887211e-13
ENSG00000145685	LHFPL2	1,725686025	7,0709464887211e-13
ENSG00000183621	ZNF438	1,296361109	8,70889614357745e-13
ENSG00000163220	S100A9	1,466631924	1,12730483700891e-12
ENSG00000187554	TLR5	1,508667535	1,40610949073872e-12
ENSG00000186818	LILRB4	1,296042303	1,51026166189786e-12
ENSG00000233461	LOC122526782	1,66301778	1,70399201205183e-12
ENSG00000090339	ICAM1	1,030124688	1,77593323814393e-12

ENSG00000106683	LIMK1	1,086621533	2,06565728038383e-12
ENSG00000100911	PSME2	1,0474357	2,18423658030404e-12
ENSG00000168394	TAP1	1,106055573	2,84800272531255e-12
ENSG00000120217	CD274	2,357115475	3,86183141476038e-12
ENSG00000138496	PARP9	1,08505665	4,41006203871662e-12
ENSG00000125900	SIRPD	1,138449831	6,18327349128035e-12
ENSG00000163754	GYG1	1,190272545	6,94299674550262e-12
ENSG00000119686	FLVCR2	1,53260216	9,96953579803279e-12
ENSG00000157551	KCNJ15	1,327522135	1,14828446957694e-11
ENSG00000167850	CD300C	1,020718353	1,25614440726579e-11
ENSG00000184060	ADAP2	1,029001839	1,42984614201201e-11
ENSG00000198216	CACNA1E	1,944725707	2,000505079392e-11
ENSG00000182541	LIMK2	1,041362788	2,62475798819483e-11
ENSG00000115415	STAT1	1,125803009	2,62994666707248e-11
ENSG00000125538	IL1B	1,081903583	2,64964956058884e-11
ENSG00000184557	SOCS3	1,69641492	3,15743622613231e-11
ENSG00000112062	MAPK14	1,098655077	3,20364403432642e-11
ENSG00000128203	ASPHD2	1,257059716	3,39082434266606e-11
ENSG00000100342	APOL1	1,34204471	3,59648422669798e-11
ENSG00000188313	PLSCR1	1,390091804	3,68340622798467e-11
ENSG00000204397	CARD16	1,412983262	3,91694291810075e-11
ENSG00000030582	GRN	1,004448037	4,23721547990497e-11
ENSG00000139832	RAB20	1,520374096	4,36986522485776e-11
ENSG00000068079	IFI35	1,292227981	4,49869024026395e-11
ENSG00000113368	LMNB1	1,180832968	4,8879071455525e-11
ENSG00000148450	MSRB2	1,152815736	5,15634700343824e-11
ENSG00000135636	DYSF	1,243171306	5,2553065906549e-11
ENSG00000166002	SMCO4	1,109327875	5,28826859294642e-11
ENSG00000152766	ANKRD22	3,365276345	6,42443929094445e-11
ENSG00000255398	HCAR3	1,48336463	6,94802697963244e-11
ENSG00000171236	LRG1	1,238083726	7,49431012674621e-11
ENSG00000178719	GRINA	1,024975496	7,61324029379329e-11
ENSG00000168899	VAMP5	1,651062788	8,58463876274507e-11
ENSG00000185482	STAC3	1,005237581	9,10244369948077e-11
ENSG00000187116	LILRA5	1,337650234	1,1236499401835e-10
ENSG00000126262	FFAR2	1,057444609	1,1456316668977e-10
ENSG00000112053	SLC26A8	1,983535717	1,28143899940854e-10
ENSG00000197903	H2BC12	1,325631012	1,37746458644464e-10
ENSG00000132274	TRIM22	1,072085644	2,07053985634323e-10
ENSG00000121858	TNFSF10	1,165790599	2,25173497730719e-10
ENSG00000221963	APOL6	1,094420081	2,36225580188734e-10
ENSG00000119457	SLC46A2	1,104733188	3,40099992743419e-10
ENSG00000091106	NLRC4	1,080490729	3,69605954954347e-10
ENSG00000180061	TMEM150B	1,486061319	5,22074497449941e-10

ENSG00000183087	GAS6	1,320739775	5,59561622887282e-10
ENSG00000010030	ETV7	2,568979266	5,75533449346477e-10
ENSG00000002549	LAP3	1,528591394	5,77773155143109e-10
ENSG00000184678	H2BC21	1,156509213	6,41435612400047e-10
ENSG00000134470	IL15RA	1,063406045	6,878517048238e-10
ENSG00000177409	SAMD9L	1,141886785	7,19939619843383e-10
ENSG00000233030	LOC124904411	2,3217529	7,81810222697705e-10
ENSG00000163568	AIM2	1,867605911	8,62261427547402e-10
ENSG00000119917	IFIT3	1,59394803	8,65761877588351e-10
ENSG00000161643	SIGLEC16	1,228914138	9,02424401697827e-10
ENSG00000140105	WARS1	1,416576934	9,05377314253377e-10
ENSG00000183347	GBP6	3,067156112	9,47660872029253e-10
ENSG00000079385	CEACAM1	1,275145179	1,0218767956977e-09
ENSG00000110080	ST3GAL4	1,067132197	1,24823313517813e-09
ENSG00000197272	IL27	1,877781453	1,34664009490352e-09
ENSG00000130222	GADD45G	1,243350074	1,51024680266946e-09
ENSG00000161944	ASGR2	1,360088196	2,2192593468672e-09
ENSG00000162747	FCGR3B	1,033600733	2,53400740592488e-09
ENSG00000137757	CASP5	1,873833808	2,7568329989304e-09
ENSG00000145365	TIFA	1,387965733	3,22216610669211e-09
ENSG00000233029	LOC100996318	2,128756994	4,49716775767757e-09
ENSG00000166523	CLEC4E	1,085129955	4,57467129625788e-09
ENSG00000170525	PFKFB3	1,099220503	5,05293061912262e-09
ENSG00000198814	GK	1,131287888	5,05548032755761e-09
ENSG00000163221	S100A12	1,637444378	5,57648064415753e-09
ENSG00000146859	TMEM140	1,001263118	5,8456344185338e-09
ENSG00000187775	DNAH17	1,085205489	7,21900664921823e-09
ENSG00000265531	FCGR1CP	3,210392517	9,65214495938922e-09
ENSG00000162512	SDC3	2,010200891	1,18781490525366e-08
ENSG00000185507	IRF7	1,225601478	1,21601211028294e-08
ENSG00000103196	CRISPLD2	1,026843078	1,25650984929506e-08
ENSG00000151726	ACSL1	1,026400275	1,29771923570358e-08
ENSG00000120885	CLU	1,408403067	1,5338171784044e-08
ENSG00000136514	RTP4	1,442567462	1,54056747766034e-08
ENSG00000182782	HCAR2	1,116717815	1,66968469244699e-08
ENSG00000163251	FZD5	1,342752657	1,82523296990358e-08
ENSG00000162433	AK4	1,774803249	2,12810874884544e-08
ENSG00000019169	MARCO	2,094615025	2,22735210407416e-08
ENSG00000166278	C2	2,343426996	2,246071095135e-08
ENSG00000162654	GBP4	1,164710792	2,2860309914844e-08
ENSG00000173369	C1QB	3,126732226	2,35586763900226e-08
ENSG00000214872	SMTNL1	2,619568582	2,48134387877329e-08
ENSG00000111199	TRPV4	1,598172897	2,505982658562e-08
ENSG00000115607	IL18RAP	1,384821688	2,53023031826169e-08
ENSG00000230539	AOAH-IT1	1,241517942	2,62519779288993e-08

ENSG00000284194	SCO2	1,395591182	2,72686441834327e-08
ENSG00000174944	P2RY14	1,796853044	3,10621175870296e-08
ENSG00000123342	MMP19	1,522097751	3,15197184754361e-08
ENSG00000134571	MYBPC3	1,005220781	3,60824093479605e-08
ENSG00000185897	FFAR3	2,067213176	4,29371365428923e-08
ENSG00000099985	OSM	1,329926202	4,33161431452842e-08
ENSG00000185339	TCN2	1,553152942	5,01359811476589e-08
ENSG00000141497	ZMYND15	1,028989951	5,14663403379675e-08
ENSG00000225131	PSME2P2	1,094460199	5,36291578205575e-08
ENSG00000158089	GALNT14	1,950237972	5,77542028042122e-08
ENSG00000173372	C1QA	2,628603465	6,07445796587668e-08
ENSG00000102796	DHRS12	1,04849236	6,12743519673153e-08
ENSG00000023171	GRAMD1B	1,113404889	6,21397472759267e-08
ENSG00000143546	S100A8	1,531180299	8,42932723397786e-08
ENSG00000204767	INSYN2B	1,119901948	9,53817840524031e-08
ENSG00000238000	PSME2P1	1,173541272	1,03122724650003e-07
ENSG00000136231	IGF2BP3	1,585141319	1,0465439538333e-07
ENSG00000126709	IFI6	1,405197648	1,05131266104153e-07
ENSG00000162772	ATF3	1,986419131	1,0691650880122e-07
ENSG00000138119	MYOF	1,507766348	1,1743320228461e-07
ENSG00000138772	ANXA3	1,548978501	1,23633025554822e-07
ENSG00000121797	CCRL2	1,081804646	1,25602527684057e-07
ENSG00000134809	TIMM10	1,513294177	1,31420691126126e-07
ENSG00000108387	SEPTIN4	3,104168647	1,50932503373813e-07
ENSG00000079215	SLC1A3	1,434401549	1,51739841805712e-07
ENSG00000183019	MCEMP1	1,722012831	1,57553616040657e-07
ENSG00000237476	LINC01637	1,002535133	1,59326977770873e-07
ENSG00000178814	OPLAH	1,306690655	1,62094004108029e-07
ENSG00000164125	GASK1B	1,013738194	1,62971929798439e-07
ENSG00000116991	SIPA1L2	1,027881645	1,68652127911388e-07
ENSG00000172159	FRMD3	1,317628507	1,69167831524325e-07
ENSG00000113763	UNC5A	1,155238761	1,87689116419109e-07
ENSG00000005961	ITGA2B	1,62149019	1,96833844694699e-07
ENSG00000179044	EXOC3L1	1,506060289	2,24630263255326e-07
ENSG00000139572	GPR84	1,800231889	2,91145979762112e-07
ENSG00000188820	CALHM6	1,996292094	2,95230958996038e-07
ENSG00000171860	C3AR1	1,142408495	2,98878655567442e-07
ENSG00000108950	FAM20A	3,308134781	3,13072445715075e-07
ENSG00000132530	XAF1	1,228964211	3,2539954144743e-07
ENSG00000162551	ALPL	1,145529691	3,41092899705896e-07
ENSG00000198785	GRIN3A	1,133291108	3,70277797513292e-07
ENSG00000197646	PDCD1LG2	2,983133147	4,12571925763535e-07
ENSG00000125148	MT2A	1,12448491	4,18424048866511e-07
ENSG00000167434	CA4	1,132049626	4,36277974569632e-07

ENSG00000123610	TNFAIP6	1,549563893	4,53798990056403e-07
ENSG00000203666	EFCAB2	1,632982644	6,06417135560475e-07
ENSG00000180712	LINC02363	1,001925337	6,18642367984312e-07
ENSG00000183307	TMEM121B	1,015955295	7,55397292576685e-07
ENSG00000159189	C1QC	3,596593803	7,71100336556077e-07
ENSG00000157227	MMP14	1,131602036	7,72902893786838e-07
ENSG00000008438	PGLYRP1	1,082396434	7,84098406117032e-07
ENSG00000100985	MMP9	1,260048181	7,89988624742247e-07
ENSG00000143545	RAB13	1,124820365	1,06419741550766e-06
ENSG00000100336	APOL4	2,863142157	1,06584406789695e-06
ENSG00000110079	MS4A4A	1,366013953	1,07796876515374e-06
ENSG00000232450	RPS2P14	1,018916993	1,09239208892268e-06
ENSG00000249437	NAIP	1,135983454	1,10504491242035e-06
ENSG00000135424	ITGA7	1,919175933	1,15870014320807e-06
ENSG00000128918	ALDH1A2	1,061315446	1,37662213987691e-06
ENSG00000287131	LOC105377156	1,382756785	1,39143204623147e-06
ENSG00000102010	BMX	1,422750236	1,48404849102708e-06
ENSG00000112299	VNN1	1,377259284	1,53146942330417e-06
ENSG00000230257	NFE4	1,105629074	1,82644897660611e-06
ENSG00000119915	ELOVL3	1,207866983	2,01650527744748e-06
ENSG00000137965	IFI44	1,605671915	2,08030917074832e-06
ENSG00000119922	IFIT2	1,010522289	2,33420839431364e-06
ENSG00000231528	FAM225A	1,8415027	2,35295246680657e-06
ENSG00000181982	CCDC149	1,151575869	2,50172575980842e-06
ENSG00000159871	LYPD5	1,951502558	2,71129869415416e-06
ENSG00000162745	OLFML2B	1,219050468	2,80597710384602e-06
ENSG00000101412	E2F1	1,052237526	2,96117888586099e-06
ENSG00000150760	DOCK1	1,318781603	2,96117888586099e-06
ENSG00000089127	OAS1	1,179433766	3,38812223944364e-06
ENSG00000101335	MYL9	1,595549696	3,70508466013011e-06
ENSG00000161911	TREML1	1,278673365	3,75277501365563e-06
ENSG00000251230	MIR3945HG	1,21828074	3,89318925674339e-06
ENSG00000005381	MPO	1,379451704	4,40447412918862e-06
ENSG00000231233	CFAP58-DT	1,097262748	4,52082992905765e-06
ENSG00000167676	PLIN4	1,003649792	4,70272753038585e-06
ENSG00000134321	RSAD2	1,755060784	4,82827986513518e-06
ENSG00000185499	MUC1	1,001410906	5,33855534634977e-06
ENSG00000158714	SLAMF8	1,267701124	5,61273437974138e-06
ENSG00000174175	SELP	1,003539812	6,73074597630191e-06
ENSG00000163814	CDCP1	1,607708631	6,8946275566872e-06
ENSG00000188282	RUFY4	1,284995106	7,47444019919468e-06
ENSG00000164403	SHROOM1	1,10660773	7,70248772488356e-06
ENSG00000029153	BMAL2	1,051003089	8,01883149760032e-06
ENSG00000175643	RMI2	1,204347803	8,16298297386044e-06
ENSG00000198829	SUCNR1	1,227659841	8,27028972844451e-06

ENSG00000203814	H2BC18	1,193638311	1,06381903372658e-05
ENSG00000156414	TDRD9	1,152426919	1,06381903372658e-05
ENSG00000277157	H4C4	1,146479745	1,08709539359069e-05
ENSG00000285906	LOC102723663	1,187141071	1,14658782508201e-05
ENSG00000113140	SPARC	1,064569642	1,23694597899928e-05
ENSG00000158373	H2BC5	1,178495463	1,2857507949523e-05
ENSG00000143595	AQP10	1,540847617	1,32381272453959e-05
ENSG00000121236	TRIM6	1,242367024	1,47476779543339e-05
ENSG00000243273	LOC124909446	1,821687744	1,50192902452797e-05
ENSG00000004809	SLC22A16	1,213908783	1,58638042215241e-05
ENSG00000248429	GASK1B-AS1	1,03725114	1,60502960187422e-05
ENSG00000185338	SOCS1	1,183499038	1,70959804201515e-05
ENSG00000112290	WASF1	1,042476375	1,74122330843718e-05
ENSG00000157168	NRG1	1,355296235	1,79871204914347e-05
ENSG00000137959	IFI44L	1,605778349	1,8752613330659e-05
ENSG00000184500	PROS1	1,283970635	1,89112772689426e-05
ENSG00000038945	MSR1	1,478825974	1,91902235646509e-05
ENSG00000109272	PF4V1	1,408915415	2,12306420691369e-05
ENSG00000290525	GBP1P1	3,178961519	2,23393612167932e-05
ENSG00000203999	LINC01270	1,04059663	2,23497931137446e-05
ENSG00000170439	TMT1B	3,815550334	2,24076744220778e-05
ENSG00000135114	OASL	1,087307296	2,35672976886299e-05
ENSG00000258227	CLEC5A	1,057820429	2,57334750865669e-05
ENSG00000223552	CCR5AS	1,321555482	2,57645822016928e-05
ENSG00000259704	LOC105370969	1,049441142	2,63304035628917e-05
ENSG00000257017	HP	2,244035376	2,89993354794736e-05
ENSG00000101425	BPI	1,493152467	2,90105146295853e-05
ENSG00000166527	CLEC4D	1,201610531	2,9799356764415e-05
ENSG00000248385	TARM1	1,994687586	3,12834108751867e-05
ENSG00000129673	AANAT	1,299538071	3,17181953780366e-05
ENSG00000289548	LOC105376995	1,439528342	3,83744494466114e-05
ENSG00000162949	CAPN13	1,627195005	3,96289643644632e-05
ENSG00000187608	ISG15	1,399600151	4,20163982481387e-05
ENSG00000237781	ADAMTSL4-AS2	1,067543699	4,2568684525825e-05
ENSG00000078399	HOXA9	1,178658085	4,27372655532761e-05
ENSG00000134326	CMPK2	1,270666031	4,28224646753137e-05
ENSG00000138798	EGF	1,203555776	4,36664785980348e-05
ENSG00000065618	COL17A1	2,009730961	4,54390262769427e-05
ENSG00000224203	RPS23P10	1,105809633	4,81758439805033e-05
ENSG00000257743	MGAM2	1,135693787	5,27605970117585e-05
ENSG00000124107	SLPI	1,241083671	5,69998076623568e-05
ENSG00000124657	OR2B6	1,086109524	5,96356397792041e-05
ENSG00000163958	ZDHHC19	1,657281293	6,76033857641095e-05

ENSG00000111331	OAS3	1,182934967	6,9059649491124e-05
ENSG00000249173	LINC01093	2,067973941	7,24879503042408e-05
ENSG00000290563	TREML3P	1,063740377	7,59013546423077e-05
ENSG00000212864	RNF208	1,04170616	7,72352878803425e-05
ENSG00000217275	RPS10P1	1,056284735	9,74069524821183e-05
ENSG00000124785	NRN1	2,218430683	9,82542209575853e-05
ENSG00000204420	MPIG6B	1,019163374	0,000103
ENSG00000099834	CDHR5	1,254074417	0,000114
ENSG00000215196	BASP1-AS1	1,096820077	0,000117
ENSG00000273802	H2BC8	1,050515252	0,000117
ENSG00000197561	ELANE	1,713076296	0,000125
ENSG00000166091	CMTM5	1,168354492	0,000127
ENSG00000123689	G0S2	1,124526911	0,000128
ENSG00000158352	SHROOM4	1,170196712	0,000129
ENSG00000161640	SIGLEC11	1,307631633	0,000162
ENSG00000259207	ITGB3	1,134570919	0,000165
ENSG00000155659	VSIG4	1,252338632	0,000168
ENSG00000138646	HERC5	1,033174087	0,00017
ENSG00000129682	FGF13	1,640365141	0,000177
ENSG00000102230	PCYT1B	1,110246271	0,000193
ENSG00000196415	PRTN3	1,8720832	0,000196
ENSG00000105048	TNNT1	1,36493767	0,000215
ENSG00000173210	ABLIM3	1,052081724	0,000299
ENSG00000115884	SDC1	1,769186616	0,000338
ENSG00000148346	LCN2	1,48565478	0,000364
ENSG00000185745	IFIT1	1,106755741	0,000365
ENSG00000249992	TMEM158	1,051454405	0,000416
ENSG00000160932	LY6E	1,002631684	0,000455
ENSG00000198753	PLXNB3	1,243479896	0,000566
ENSG00000198959	TGM2	1,101290004	0,00063
ENSG00000158578	ALAS2	1,162410451	0,000706
ENSG00000079393	DUSP13B	1,165317517	0,000714
ENSG00000106853	PTGR1	1,253410341	0,000714
ENSG00000170955	CAVIN3	1,247542775	0,000742
ENSG00000186583	SPATC1	1,409570462	0,000801
ENSG00000156113	KCNMA1	1,582346356	0,00085
ENSG00000168528	SERINC2	1,123520045	0,001063
ENSG00000164047	CAMP	1,080351701	0,001065
ENSG00000111181	SLC6A12	1,471663091	0,001187
ENSG00000012223	LTF	1,558596155	0,001235
ENSG00000086548	CEACAM6	1,434429488	0,001246
ENSG00000169245	CXCL10	1,331230415	0,001304
ENSG00000249502	MED28-DT	1,10702673	0,00131
ENSG00000172232	AZU1	1,238741948	0,001357
ENSG00000123838	C4BPA	1,9458628	0,001989

ENSG00000167100	SAMD14	1,271943357	0,002411
ENSG00000118113	MMP8	1,943255436	0,002638
ENSG00000164850	GPER1	1,069738561	0,002689
ENSG00000226004	SIMALR	1,947961619	0,002751
ENSG00000145555	MYO10	1,164120501	0,002755
ENSG00000204936	CD177	2,747941498	0,002892
ENSG00000167105	TMEM92	1,057815818	0,003247
ENSG00000164821	DEFA4	1,517985624	0,003546
ENSG00000229644	NAMPTP1	1,067860147	0,00364
ENSG00000088827	SIGLEC1	1,190787181	0,00453
ENSG00000250138	LOC728488	1,129185933	0,005694
ENSG00000211669	IGLV3-10	1,233815423	0,007218
ENSG00000096006	CRISP3	1,160930759	0,008173
ENSG00000102837	OLFM4	1,615038285	0,010076
ENSG00000283646	LINC02009	1,326272565	0,010323
ENSG00000076864	RAP1GAP	1,828808913	0,010716
ENSG00000129538	RNASE1	1,308134595	0,011624
ENSG00000100448	CTSG	1,217644135	0,012962
ENSG00000124469	CEACAM8	1,311954653	0,014395
ENSG00000290878	HLA-DRB6	1,823167348	0,019543
ENSG00000134201	GSTM5	1,067767731	0,020458
ENSG00000275063	LOC102723407	1,293654188	0,022283
ENSG00000178752	ERFE	1,480963422	0,025672
ENSG00000211639	IGLV4-60	1,05041389	0,026232
ENSG00000106565	TMEM176B	1,037436529	0,029755
ENSG00000103723	AP3B2	1,109440349	0,033515
ENSG00000164237	CMBL	1,038580115	0,035941
ENSG00000118473	SGIP1	1,031070983	0,039536
ENSG00000183844	FAM3B	1,535081702	0,047665
ENSG00000144645	OSBPL10	-1,340845523	5,91199794017766e-14
ENSG00000105492	SIGLEC6	-1,346450631	1,34507819917436e-13
ENSG00000124171	PARD6B	-1,090667893	2,3728363247476e-13
ENSG00000126016	AMOT	-1,012205129	3,73615567206235e-13
ENSG00000144824	PHLDB2	-1,234805661	3,91605375400508e-13
ENSG00000110318	CEP126	-1,139578865	8,70889614357745e-13
ENSG00000162545	CAMK2N1	-1,134903167	1,50917262351134e-12
ENSG00000169031	COL4A3	-1,536928556	4,46206171249897e-12
ENSG00000153064	BANK1	-1,189345127	7,1344801063607e-12
ENSG00000164330	EBF1	-1,394822419	7,98913193732076e-12
ENSG00000081052	COL4A4	-1,057398965	9,46999444334431e-12
ENSG00000116106	EPHA4	-1,200261334	9,57331635566387e-12
ENSG00000120278	PLEKHG1	-1,104226619	1,05442408342206e-11
ENSG00000082438	COBLL1	-1,108631198	2,82781297652597e-11
ENSG00000078589	P2RY10	-1,129449502	3,73994855501955e-11

ENSG00000196172	ZNF681	-1,218872991	3,75833333996092e-11
ENSG00000169508	GPR183	-1,082373579	4,36854829840776e-11
ENSG00000182389	CACNB4	-1,591773809	4,98162074120253e-11
ENSG00000176928	GCNT4	-1,104650606	6,5182201020395e-11
ENSG00000237940	LINC01238	-1,07858994	7,10754100375176e-11
ENSG00000133424	LARGE1	-1,190758536	7,91097052780442e-11
ENSG00000030419	IKZF2	-1,174339999	8,73466863502222e-11
ENSG00000144218	AFF3	-1,11016971	1,150741709741e-10
ENSG00000173258	ZNF483	-1,048021353	1,46937890030889e-10
ENSG00000188848	BEND4	-1,181271134	2,1238353733659e-10
ENSG00000100473	COCH	-1,312679293	2,8331433677604e-10
ENSG00000132185	FCRLA	-1,245184781	2,86515231920863e-10
ENSG00000069667	RORA	-1,088169756	3,29640610431265e-10
ENSG00000152894	PTPRK	-1,316244092	4,1732297261472e-10
ENSG00000143869	GDF7	-1,50746329	5,10831663182489e-10
ENSG00000214595	EML6	-1,095970415	5,26339915352494e-10
ENSG00000184226	PCDH9	-1,518418568	7,53414285234938e-10
ENSG00000132704	FCRL2	-1,245223387	9,27658609974487e-10
ENSG00000033867	SLC4A7	-1,010850069	9,81634536605981e-10
ENSG00000106537	TSPAN13	-1,015940593	1,38405472128197e-09
ENSG00000171992	SYNPO	-1,159797868	1,78978854864988e-09
ENSG00000101230	ISM1	-1,384961727	2,08852924081132e-09
ENSG00000196092	PAX5	-1,27300488	2,4453904392805e-09
ENSG00000165905	LARGE2	-1,012948962	2,49210467979155e-09
ENSG00000234965	SHISA8	-1,365316702	3,4789138104698e-09
ENSG00000206077	ZDHHC11B	-1,353376415	3,66213097593379e-09
ENSG00000160683	CXCR5	-1,031949372	4,04545680217639e-09
ENSG00000108924	HLF	-1,175195774	5,32463119017128e-09
ENSG00000213809	KLRK1	-1,022391909	7,31924041352734e-09
ENSG00000197978	GOLGA6L9	-1,184746901	7,66643501116619e-09
ENSG00000235621	LINC00494	-1,209644757	1,02358977430882e-08
ENSG00000116983	HPCAL4	-1,05967791	1,10515174321259e-08
ENSG00000185697	MYBL1	-1,142004961	1,23810096677876e-08
ENSG00000154764	WNT7A	-1,008729447	1,31106296156663e-08
ENSG00000159231	CBR3	-1,074193804	1,40926429636618e-08
ENSG00000269404	SPIB	-1,013250686	1,53069527717947e-08
ENSG00000196911	KPNA5	-1,020592391	1,53730479690398e-08
ENSG00000159958	TNFRSF13C	-1,198375884	1,6586965564496e-08
ENSG00000109654	TRIM2	-1,1540775	1,66968469244699e-08
ENSG00000164114	MAP9	-1,02662978	1,72846082047946e-08
ENSG00000157404	KIT	-1,067962994	2,17347362102499e-08
ENSG00000002587	HS3ST1	-1,143073488	3,15197184754361e-08
ENSG00000275395	FCGBP	-1,089387265	3,29967766610448e-08
ENSG00000156738	MS4A1	-1,390729417	3,55045296764784e-08
ENSG00000233806	LINC01237	-1,014175678	3,56915905440746e-08

ENSG00000235532	LINC00402	-1,13952174	4,00120679317415e-08
ENSG00000134709	HOOK1	-1,074503814	4,03322538238473e-08
ENSG00000153253	SCN3A	-1,447466253	4,37934655426739e-08
ENSG00000123219	CENPK	-1,242800118	5,82285030453268e-08
ENSG00000171368	TPPP	-1,004666605	6,54501353034353e-08
ENSG00000136573	BLK	-1,043686496	6,56227537254419e-08
ENSG00000172264	MACROD2	-1,586425827	6,88265421104379e-08
ENSG00000227218	LOC124902280	-1,130590645	6,88265421104379e-08
ENSG00000183960	KCNH8	-1,488820913	7,23829595948243e-08
ENSG00000179088	C12orf42	-1,041458044	9,22906702552703e-08
ENSG00000138311	ZNF365	-1,22991715	9,54111276212338e-08
ENSG00000181690	PLAG1	-1,020233097	9,93255784792471e-08
ENSG00000165810	BTNL9	-1,707359366	1,24706794926833e-07
ENSG00000286330	LOC105370259	-1,120479327	1,31685877988293e-07
ENSG00000170128	GPR25	-1,095002505	1,41709091824494e-07
ENSG00000091129	NRCAM	-1,703677367	1,67688833149938e-07
ENSG00000178947	SMIM10L2A	-1,049083737	1,81614206733546e-07
ENSG00000249667	LINC01259	-1,108135125	3,03919478622804e-07
ENSG00000178075	GRAMD1C	-1,003344719	3,04951279543557e-07
ENSG00000135525	MAP7	-1,347933403	3,37238921204868e-07
ENSG00000112394	SLC16A10	-1,03889122	6,01665588185582e-07
ENSG00000163520	FBLN2	-1,205651568	1,06514638940002e-06
ENSG00000163534	FCRL1	-1,135045833	1,06594058362428e-06
ENSG00000128218	VPREB3	-1,057838088	1,08227854981693e-06
ENSG00000167483	NIBAN3	-1,073421667	1,26641803565908e-06
ENSG00000162630	B3GALT2	-1,065484244	1,27088401546784e-06
ENSG00000082293	COL19A1	-1,102899582	1,3322162212954e-06
ENSG00000277586	NEFL	-1,040891907	1,37560789418579e-06
ENSG00000126838	PZP	-1,022809567	1,38513980636772e-06
ENSG00000247982	LINC00926	-1,096722029	1,43500213873815e-06
ENSG00000211689	TRGC1	-1,153462938	2,00945658660846e-06
ENSG00000105369	CD79A	-1,081260099	2,30045647952653e-06
ENSG00000250696	LOC105377267	-1,667499738	3,46606419501816e-06
ENSG00000050165	DKK3	-1,397233596	4,59443105620543e-06
ENSG00000135116	HRK	-1,474547656	5,11713598160903e-06
ENSG00000005471	ABCB4	-1,069156145	9,02098568386007e-06
ENSG00000120057	SFRP5	-1,391765413	1,44318973719918e-05
ENSG00000054179	ENTPD2	-1,490208135	3,0837562512711e-05
ENSG00000103647	CORO2B	-1,078087095	5,15022633870432e-05
ENSG00000096696	DSP	-2,29915713	6,86152501378977e-05
ENSG00000122733	PHF24	-1,341973495	0,000105
ENSG00000130300	PLVAP	-1,057911584	0,000146
ENSG00000174469	CNTNAP2	-1,426826701	0,000157
ENSG00000211821	TRDV2	-1,18858647	0,000201

ENSG00000175894	TSPEAR	-1,571559329	0,000287
ENSG00000066468	FGFR2	-1,127891694	0,00031
ENSG00000215252	GOLGA8B	-1,021427793	0,000347
ENSG00000169507	SLC38A11	-1,00379452	0,001941
ENSG00000118785	SPP1	-1,109727324	0,002807
ENSG00000158748	HTR6	-1,085381806	0,003322
ENSG00000161905	ALOX15	-1,029404546	0,005453
ENSG00000106714	CNTNAP3	-1,139067765	0,028458
ENSG00000291037	ZNF890P	-1,118651143	0,028976
ENSG00000230734	RPL10P3	-1,198368743	0,032858

Tabel S.**25.** Coefficient of variant for 99 genes among

Ensemple ID	Gene Symbol	Coeff Variation
ENSG00000214548	MEG3	1,54728719
ENSG00000214652	ZNF727	1,08678
ENSG00000187244	BCAM	0,998970731
ENSG00000101883	RHOXF1	0,86918612
ENSG00000206177	HBM	0,72323489
ENSG00000174469	CNTNAP2	0,712234663
ENSG00000151320	AKAP6	0,673635596
ENSG00000175894	TSPEAR	0,62848022
ENSG00000143416	SELENBP1	0,574080033
ENSG00000223609	HBD	0,554578297
ENSG00000088340	FER1L4	0,529829217
ENSG00000186081	KRT5	0,525981965
ENSG00000213557	RPL31P43	0,498761842
ENSG00000234184	LINC01781	0,491288785
ENSG00000278897	Novel transcript	0,489314429
ENSG00000172264	MACROD2	0,489057293
ENSG00000115884	SDC1	0,485211972
ENSG00000197520	FAM177B	0,471710358
ENSG00000255733	IFNG-AS1	0,457062158
ENSG00000135116	HRK	0,456656196
ENSG00000248385	TARM1	0,454005474
ENSG00000183960	KCNH8	0,447867632
ENSG00000158578	ALAS2	0,438116241
ENSG00000289278	Novel transcript	0,435255792
ENSG00000112486	CCR6	0,432241761
ENSG00000258732	Novel transcript	0,399433174
ENSG00000206077	ZDHHC11B	0,394681954
ENSG00000169031	COL4A3	0,388136259
ENSG00000112394	SLC16A10	0,386487612
ENSG00000197353	LYPD2	0,377490078
ENSG00000215630	GUSBP9	0,374801655
ENSG00000128536	CDHR3	0,36562969
ENSG00000152894	PTPRK	0,344517306
ENSG00000124785	NRN1	0,33983593
ENSG00000133424	LARGE1	0,338986469
ENSG00000100095	SEZ6L	0,325479378
ENSG00000198753	PLXNB3	0,324345307
ENSG00000164330	EBF1	0,309410564
ENSG00000173585	CCR9	0,301945314
ENSG00000144290	SLC4A10	0,300147258
ENSG00000211821	TRDV2	0,296162502

ENSG00000211806	TRAV25	0,293952301
ENSG00000224940	PRRT4	0,289032663
ENSG00000173114	LRRN3	0,272350861
ENSG00000258511	Novel transcript	0,268264434
ENSG00000242550	SERPINB10	0,267204759
ENSG00000163814	CDCP1	0,266651927
ENSG00000196415	PRTN3	0,252304439
ENSG00000163520	FBLN2	0,250898885
ENSG00000275395	FCGBP	0,247948226
ENSG00000185897	FFAR3	0,247558675
ENSG00000255221	Novel transcript	0,244511311
ENSG00000169397	RNASE3	0,23162495
ENSG00000154027	AK5	0,229958871
ENSG00000167680	SEMA6B	0,222065842
ENSG00000122861	PLAU	0,221569747
ENSG00000284194	SCO2	0,209376067
ENSG00000204936	CD177	0,197983622
ENSG00000134545	KLRC1	0,195236982
ENSG00000157168	NRG1	0,195138012
ENSG00000172232	AZU1	0,194723777
ENSG00000108950	FAM20A	0,194686996
ENSG00000183347	GBP6	0,190617044
ENSG00000239839	DEFA3	0,190185085
ENSG00000235532	LINC00402	0,187869511
ENSG00000132185	FCRLA	0,186946246
ENSG00000197561	ELANE	0,181232609
ENSG00000198959	TGM2	0,179802055
ENSG00000173369	C1QB	0,179699539
ENSG00000156738	MS4A1	0,177715727
ENSG00000168528	SERINC2	0,174004384
ENSG00000118113	MMP8	0,17334171
ENSG00000233030	LOC124904411	0,169088203
ENSG00000163958	ZDHHC19	0,167630408
ENSG00000184613	NELL2	0,16664859
ENSG00000133063	CHIT1	0,164140666
ENSG00000087237	CETP	0,154141986
ENSG00000113088	GZMK	0,153929859
ENSG00000257017	HP	0,151032259
ENSG00000134321	RSAD2	0,149884149
ENSG00000249992	TMEM158	0,145119773
ENSG00000291135	FCGR1BP	0,145083099
ENSG00000130202	NECTIN2	0,132140623
ENSG00000168062	BATF2	0,129620519
ENSG00000188820	CALHM6	0,125065433
ENSG00000120217	CD274	0,119250561

ENSG00000145685	LHFPL2	0,103138812
ENSG00000106070	GRB10	0,098258355
ENSG00000185909	KLHDC8B	0,091826467
ENSG00000074660	SCARF1	0,086133122
ENSG00000177989	ODF3B	0,082344142
ENSG00000152229	PSTPIP2	0,077980157
ENSG00000075399	VPS9D1	0,076931284
ENSG00000142089	IFITM3	0,072130853
ENSG00000082014	SMARCD3	0,06682765
ENSG00000008438	PGLYRP1	0,061979932
ENSG00000168899	VAMP5	0,060674162
ENSG00000138772	ANXA3	0,054732812
ENSG00000143546	S100A8	0,040476522
